# Supplementary material for: Integrative Proteomic and Phosphoproteomic Analyses Revealed Complex Mechanisms Underlying Reproductive Diapause in Bombus terrestris Queens
Source: Insects. 2022 Sep 23;13(10):862. doi: 10.3390/insects13100862 (PMC9604461; doi:10.3390/insects13100862)

Supplementary Figure S1: Volcano plots from different group comparisons in proteome (A) and phosphoproteome (B), red dots represent fold change >1.3 and p<0.05, green dots represent fold change<0.77 and p<0.05, grey dots represent unsignificantly changed proteins or phosphorylation sites.

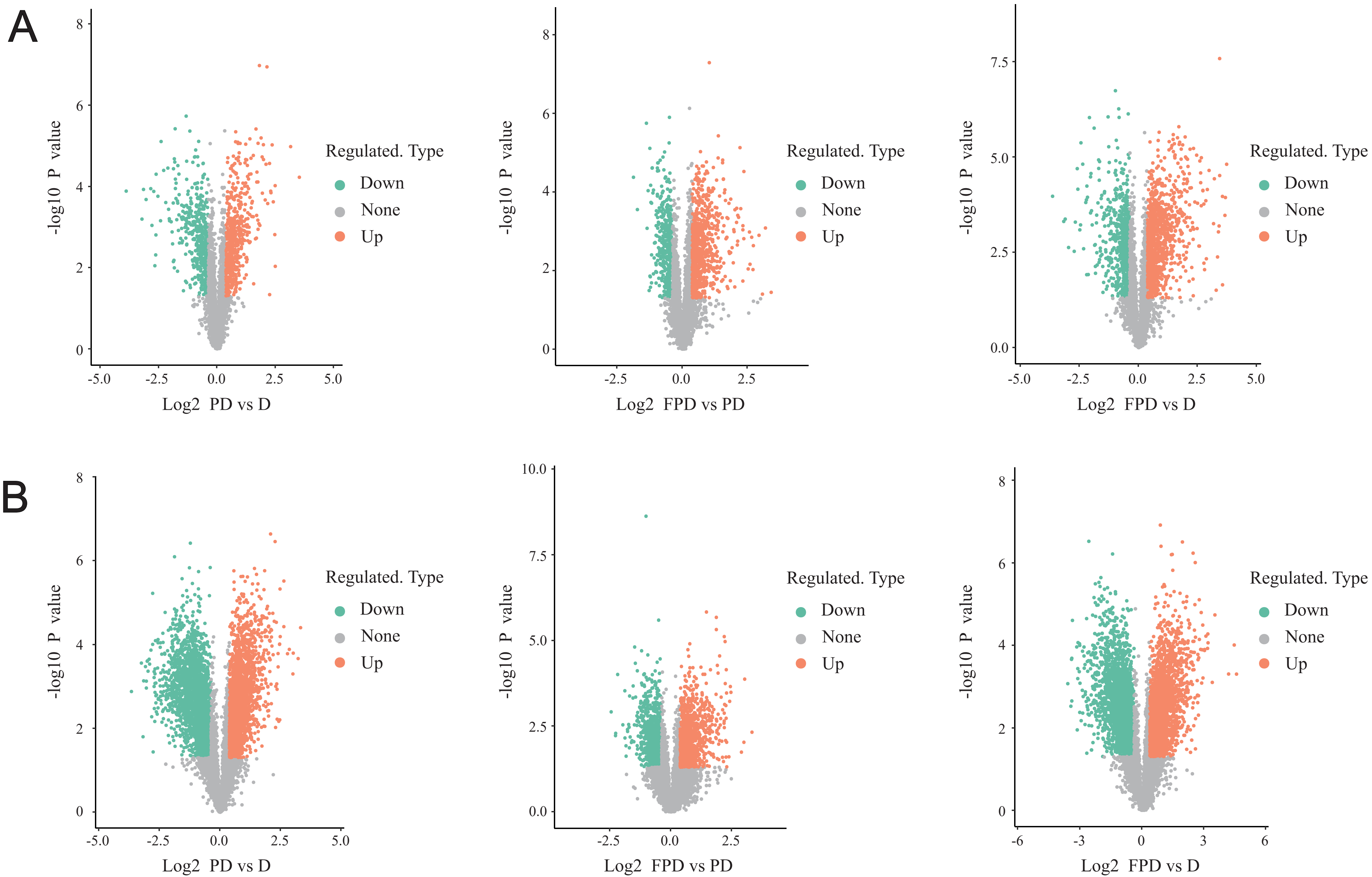

Supplement: Supplementary file 1 [file insects-13-00862-s001.zip › insects-1876268-supplementary/insects-1876268-proofed-supplementary/Figure S1.pdf]
