# Supplementary material for: Integrative Proteomic and Phosphoproteomic Analyses Revealed Complex Mechanisms Underlying Reproductive Diapause in Bombus terrestris Queens
Source: Insects. 2022 Sep 23;13(10):862. doi: 10.3390/insects13100862 (PMC9604461; doi:10.3390/insects13100862)
Supplement: Supplementary file 1 [file insects-13-00862-s001.zip › insects-1876268-supplementary/insects-1876268-proofed-supplementary/Figure S10.pdf]

Supplementary Figure S10: GO analysis of DEPPs in each comparable group.

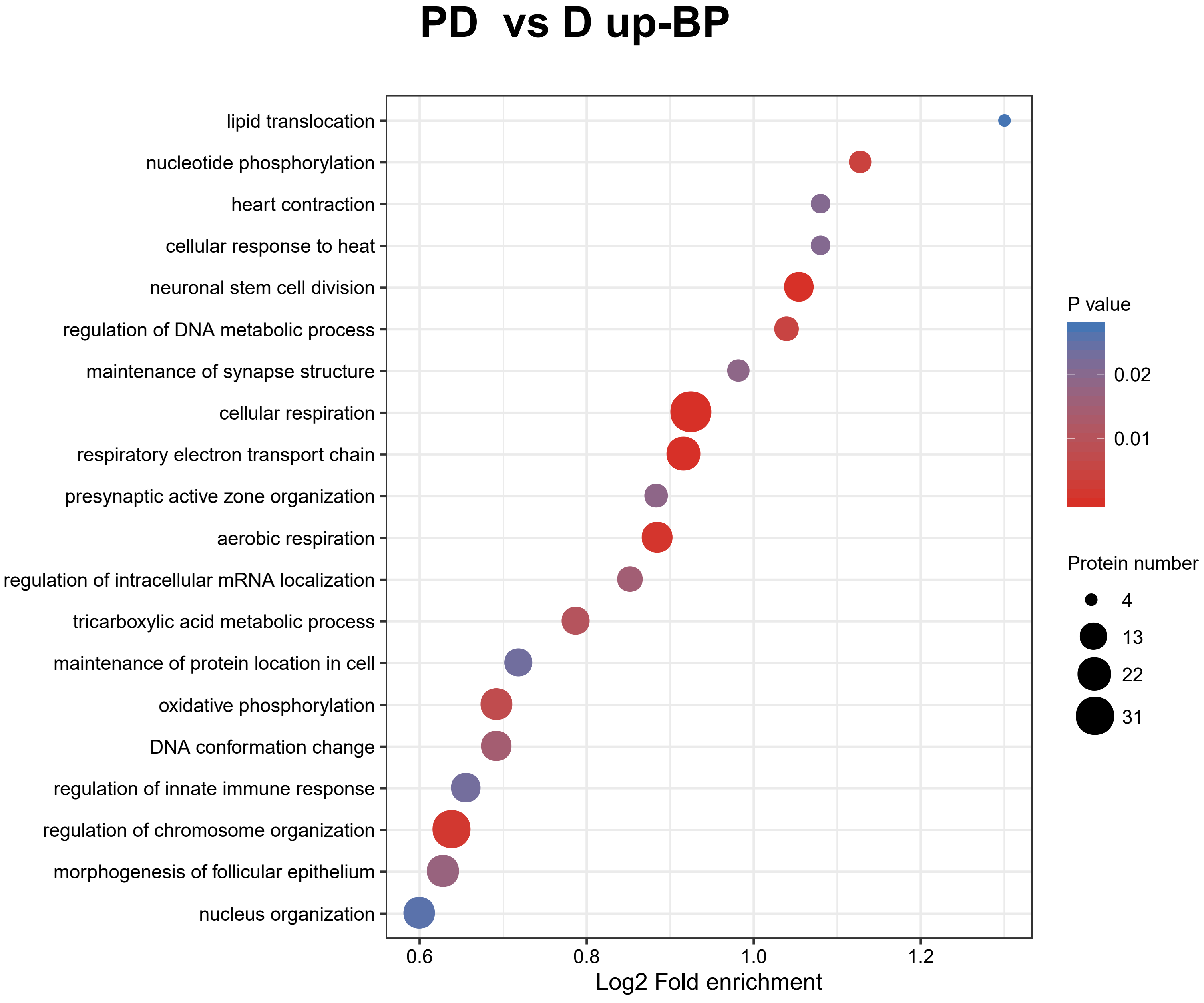

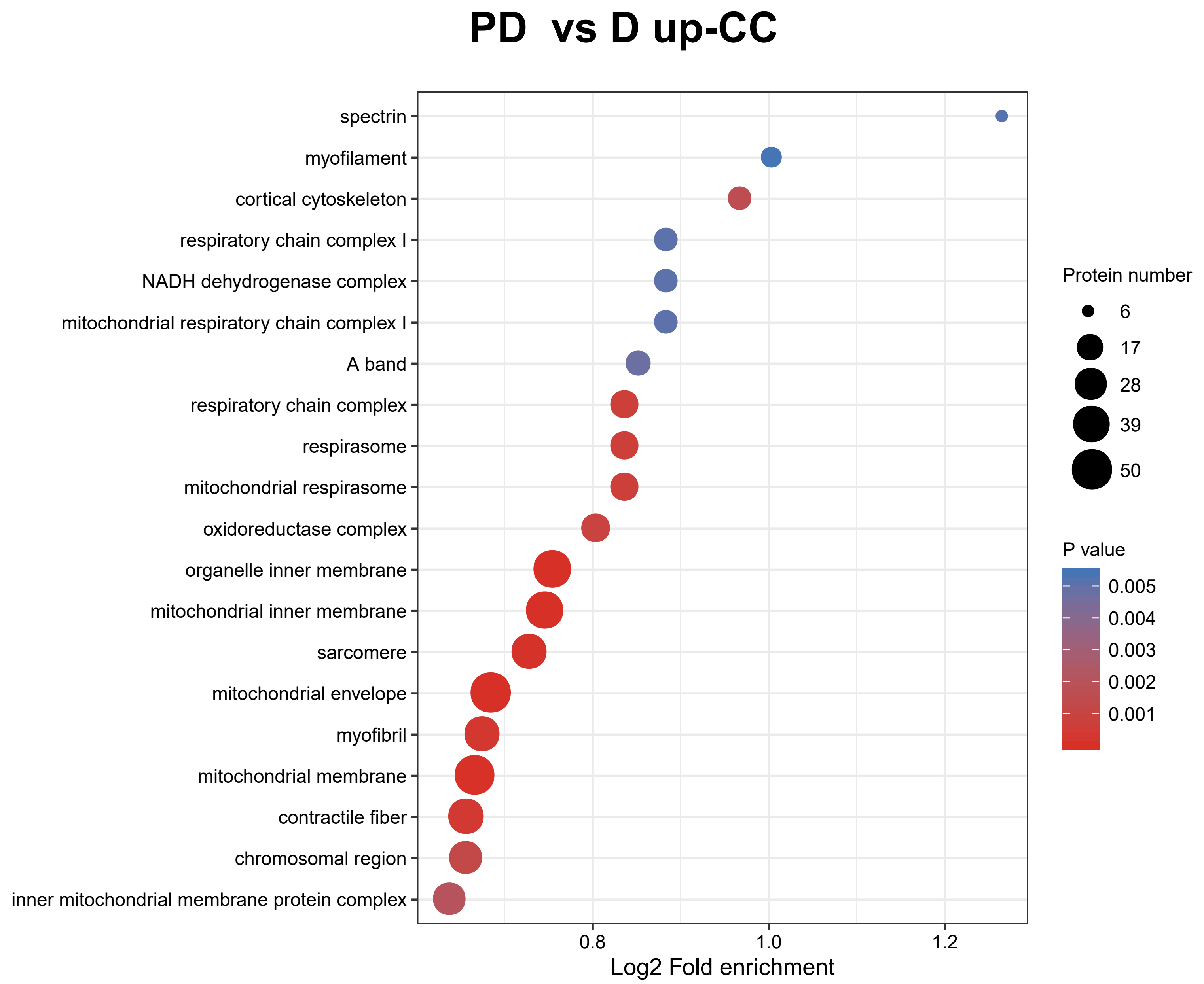

PD vs D up-CC
spectrin
myofilament
cortical cytoskeleton
respiratory chain complex I
NADH dehydrogenase complex
mitochondrial respiratory chain complex I
A band
respiratory chain complex
respirasome
mitochondrial respirasome
oxidoreductase complex
organelle inner membrane
mitochondrial inner membrane
sarcomere
mitochondrial envelope
myofibril
mitochondrial membrane
contractile fiber
chromosomal region
inner mitochondrial membrane protein complex
0.8
1.0
1.2
Log2 Fold enrichment
Protein number
6
17
28
39
50
P value
0.005
0.004
0.003
0.002
0.001

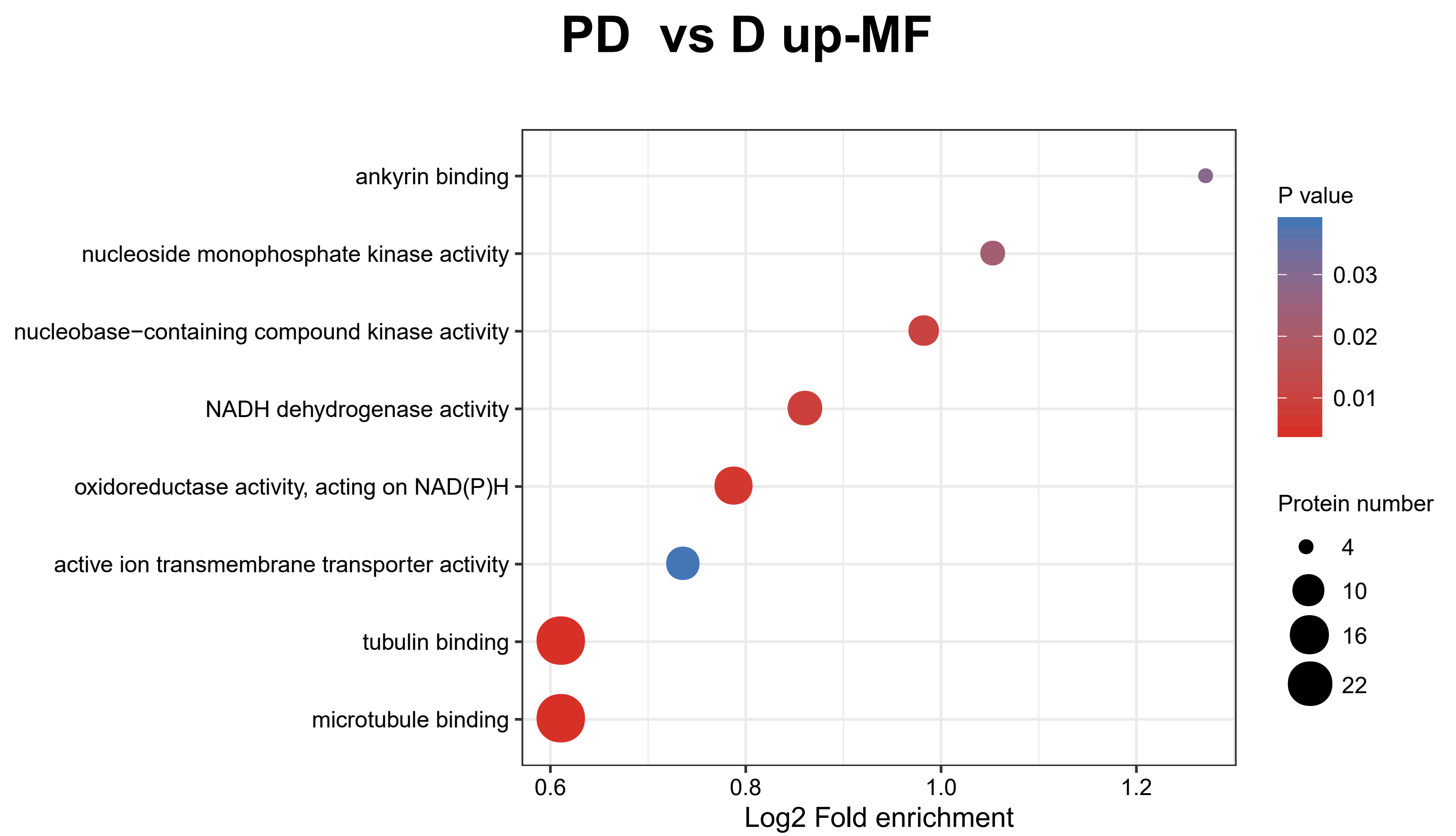
PD vs D up-MF
ankyrin binding
nucleoside monophosphate kinase activity
nucleobase−containing compound kinase activity
NADH dehydrogenase activity
oxidoreductase activity, acting on NAD(P)H
active ion transmembrane transporter activity
tubulin binding
microtubule binding
0.6
0.8
1.0
1.2
Log2 Fold enrichment
P value
0.03
0.02
0.01
Protein number
4
10
16
22

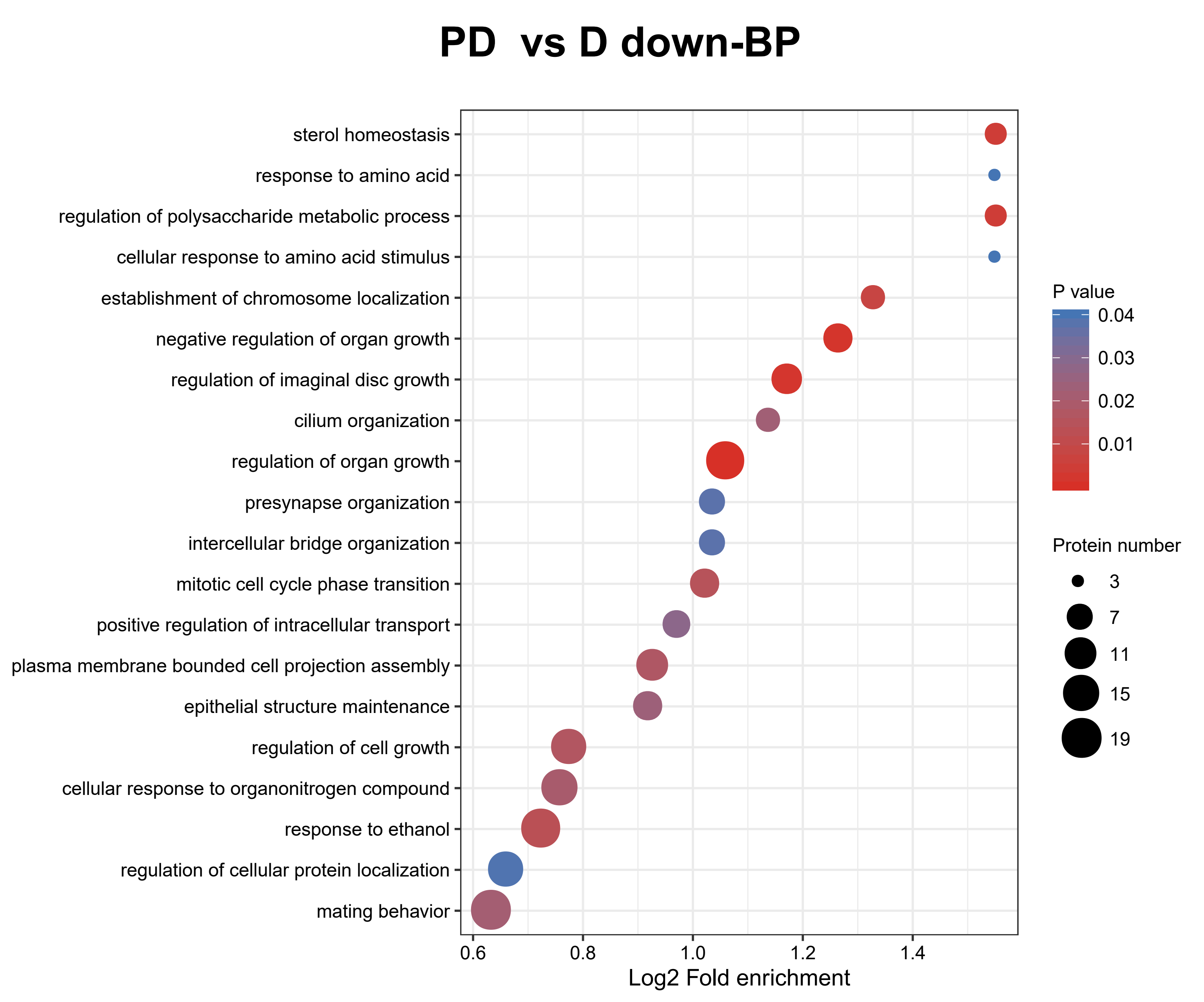

PD vs D down-BP
sterol homeostasis
response to amino acid
regulation of polysaccharide metabolic process
cellular response to amino acid stimulus
establishment of chromosome localization
negative regulation of organ growth
regulation of imaginal disc growth
cilium organization
regulation of organ growth
presynapse organization
intercellular bridge organization
mitotic cell cycle phase transition
positive regulation of intracellular transport
plasma membrane bounded cell projection assembly
epithelial structure maintenance
regulation of cell growth
cellular response to organonitrogen compound
response to ethanol
regulation of cellular protein localization
mating behavior
0.6
0.8
1.0
1.2
1.4
Log2 Fold enrichment
P value
0.04
0.03
0.02
0.01
Protein number
3
7
11
15
19

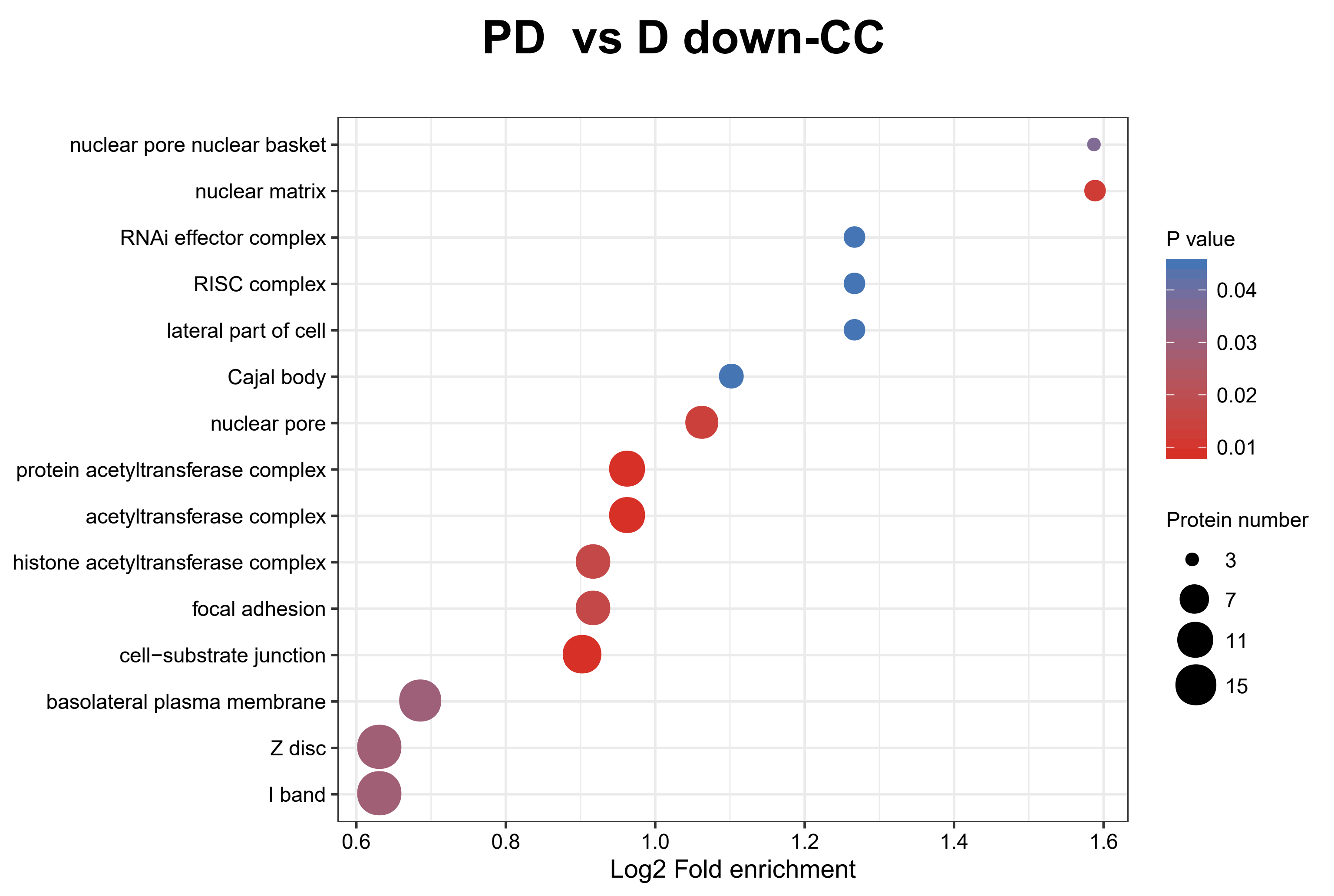

PD vs D down-CC
nuclear pore nuclear basket
nuclear matrix
RNAi effector complex
RISC complex
lateral part of cell
Cajal body
nuclear pore
protein acetyltransferase complex
acetyltransferase complex
histone acetyltransferase complex
focal adhesion
cell−substrate junction
basolateral plasma membrane
Z disc
I band
0.6
0.8
1.0
1.2
1.4
1.6
Log2 Fold enrichment
P value
0.04
0.03
0.02
0.01
Protein number
3
7
11
15

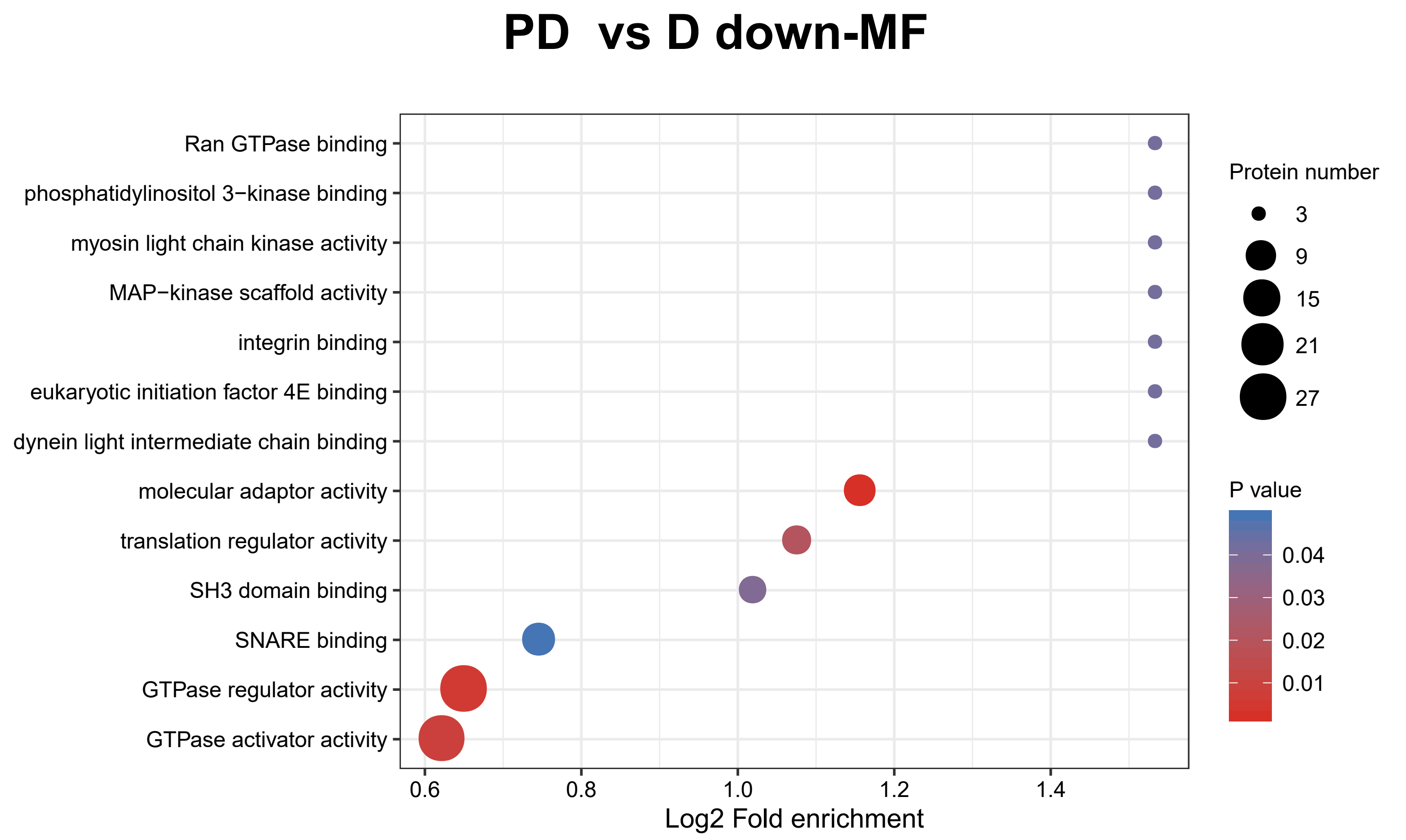
PD vs D down-MF
Ran GTPase binding
phosphatidylinositol 3−kinase binding
myosin light chain kinase activity
MAP−kinase scaffold activity
integrin binding
eukaryotic initiation factor 4E binding
dynein light intermediate chain binding
molecular adaptor activity
translation regulator activity
SH3 domain binding
SNARE binding
GTPase regulator activity
GTPase activator activity
0.6
0.8
1.0
1.2
1.4
Log2 Fold enrichment
Protein number
3
9
15
21
27
P value
0.04
0.03
0.02
0.01

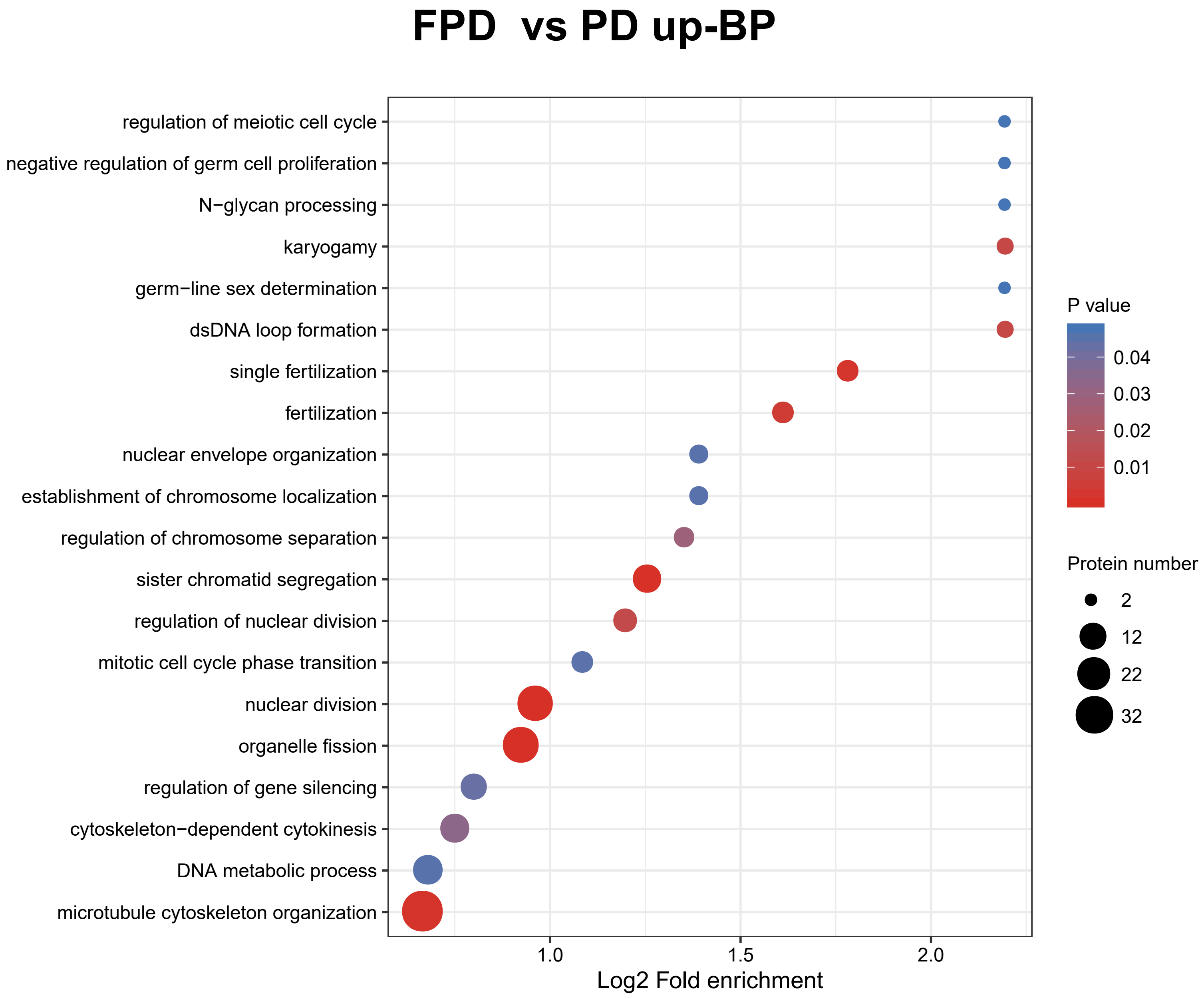
FPD vs PD up-BP
regulation of meiotic cell cycle
negative regulation of germ cell proliferation
N−glycan processing
karyogamy
germ−line sex determination
dsDNA loop formation
single fertilization
fertilization
nuclear envelope organization
establishment of chromosome localization
regulation of chromosome separation
sister chromatid segregation
regulation of nuclear division
mitotic cell cycle phase transition
nuclear division
organelle fission
regulation of gene silencing
cytoskeleton−dependent cytokinesis
DNA metabolic process
microtubule cytoskeleton organization
1.0
1.5
2.0
Log2 Fold enrichment
P value
0.04
0.03
0.02
0.01
Protein number
2
12
22
32

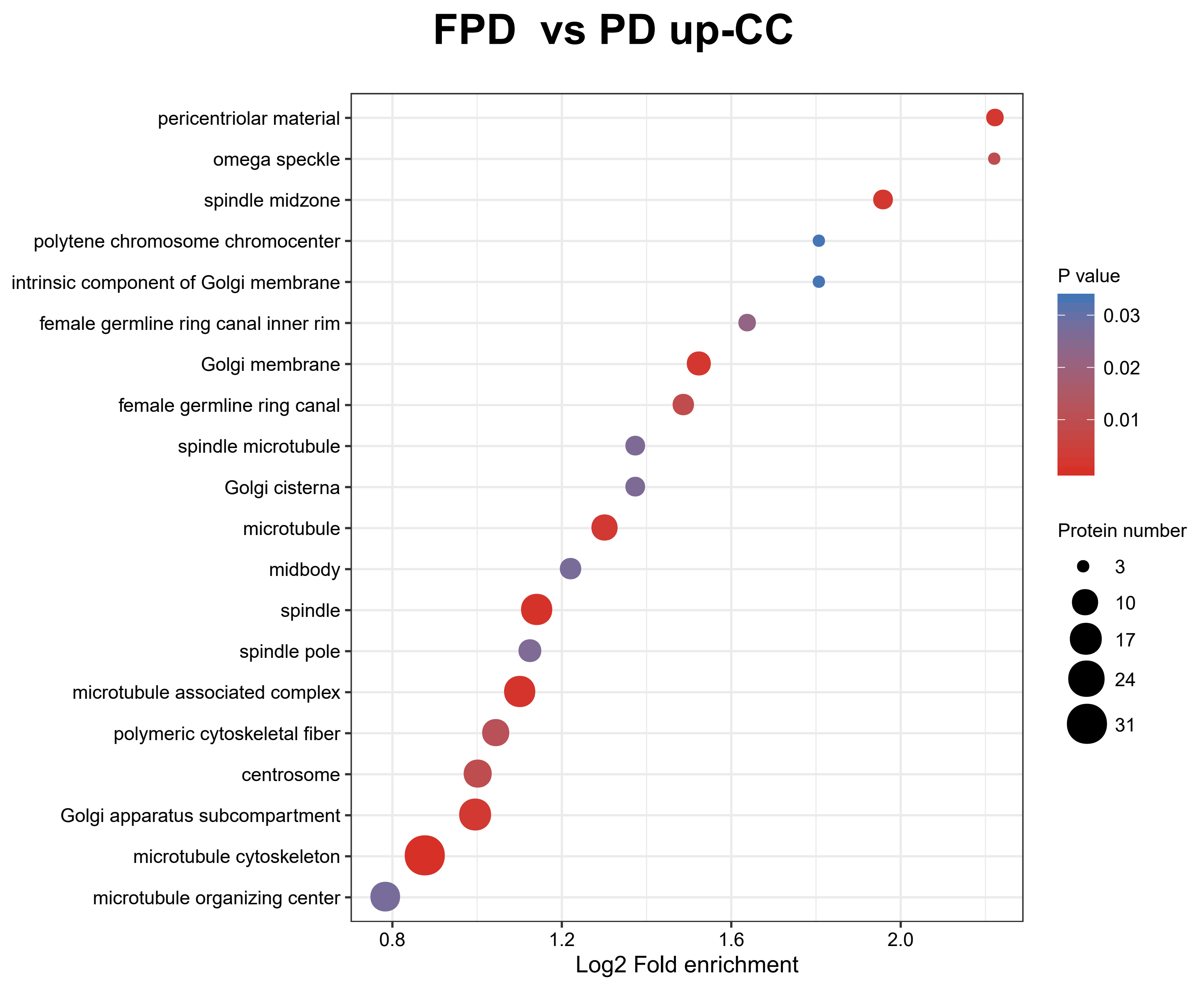
FPD vs PD up-CC
pericentriolar material
omega speckle
spindle midzone
polytene chromosome chromocenter
intrinsic component of Golgi membrane
female germline ring canal inner rim
Golgi membrane
female germline ring canal
spindle microtubule
Golgi cisterna
microtubule
midbody
spindle
spindle pole
microtubule associated complex
polymeric cytoskeletal fiber
centrosome
Golgi apparatus subcompartment
microtubule cytoskeleton
microtubule organizing center
0.8
1.2
1.6
2.0
Log2 Fold enrichment
P value
0.03
0.02
0.01
Protein number
3
10
17
24
31

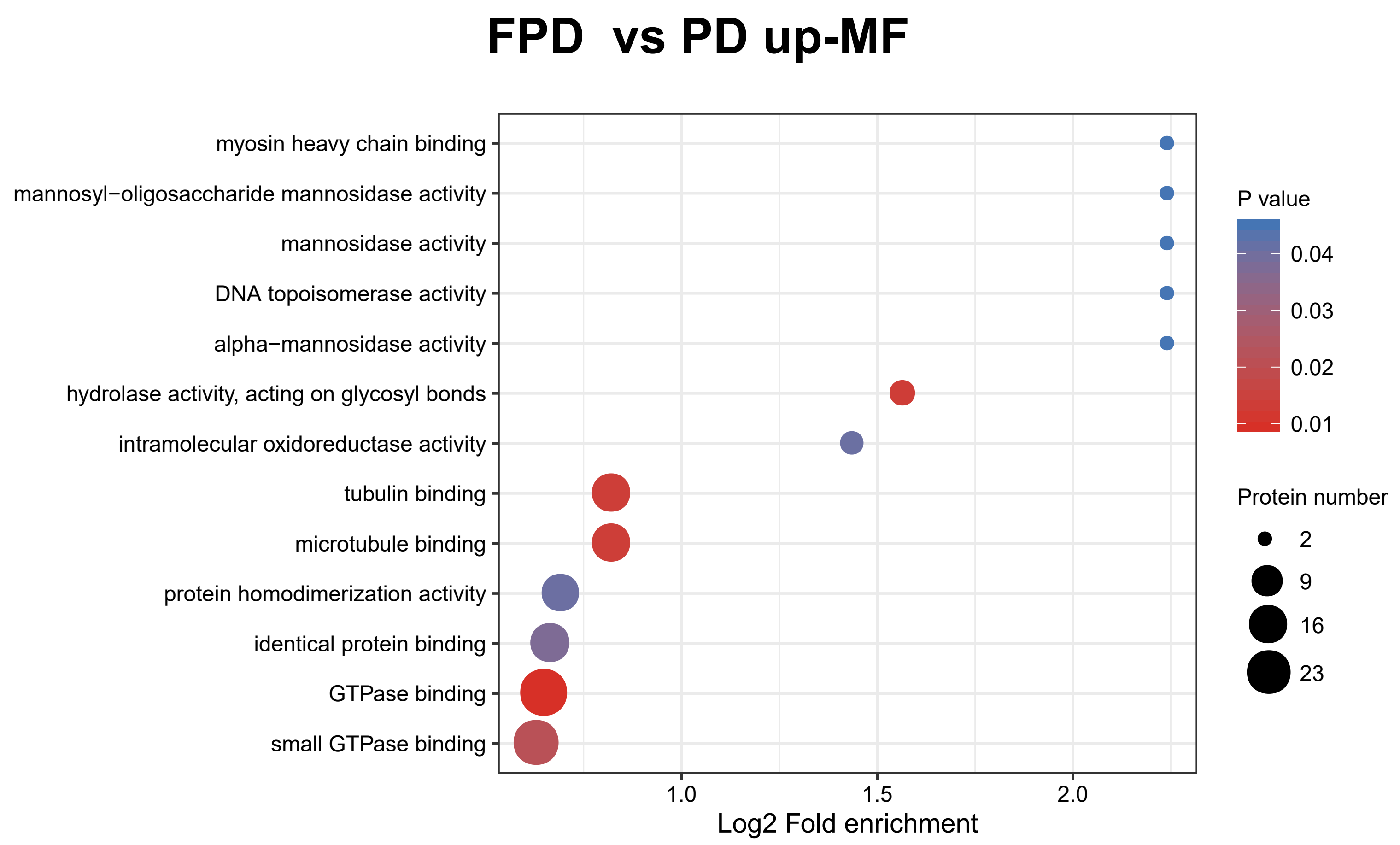

FPD vs PD up-MF
myosin heavy chain binding
mannosyl−oligosaccharide mannosidase activity
mannosidase activity
DNA topoisomerase activity
alpha−mannosidase activity
hydrolase activity, acting on glycosyl bonds
intramolecular oxidoreductase activity
tubulin binding
microtubule binding
protein homodimerization activity
identical protein binding
GTPase binding
small GTPase binding
1.0
1.5
2.0
Log2 Fold enrichment
P value
0.04
0.03
0.02
0.01
Protein number
2
9
16
23

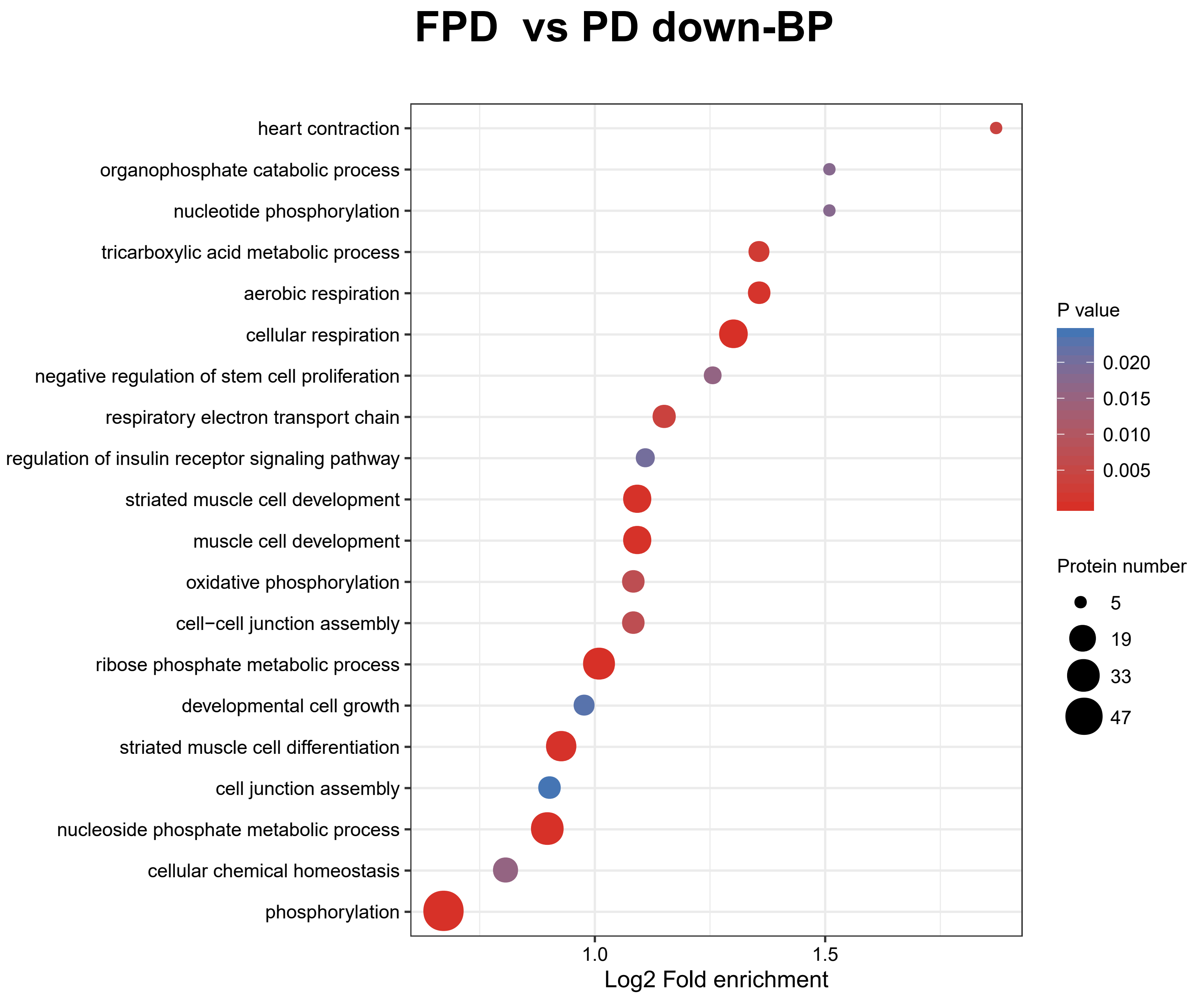
FPD vs PD down-BP
heart contraction
organophosphate catabolic process
nucleotide phosphorylation
tricarboxylic acid metabolic process
aerobic respiration
cellular respiration
negative regulation of stem cell proliferation
respiratory electron transport chain
regulation of insulin receptor signaling pathway
striated muscle cell development
muscle cell development
oxidative phosphorylation
cell−cell junction assembly
ribose phosphate metabolic process
developmental cell growth
striated muscle cell differentiation
cell junction assembly
nucleoside phosphate metabolic process
cellular chemical homeostasis
phosphorylation
1.0
1.5
Log2 Fold enrichment
P value
0.020
0.015
0.010
0.005
Protein number
5
19
33
47

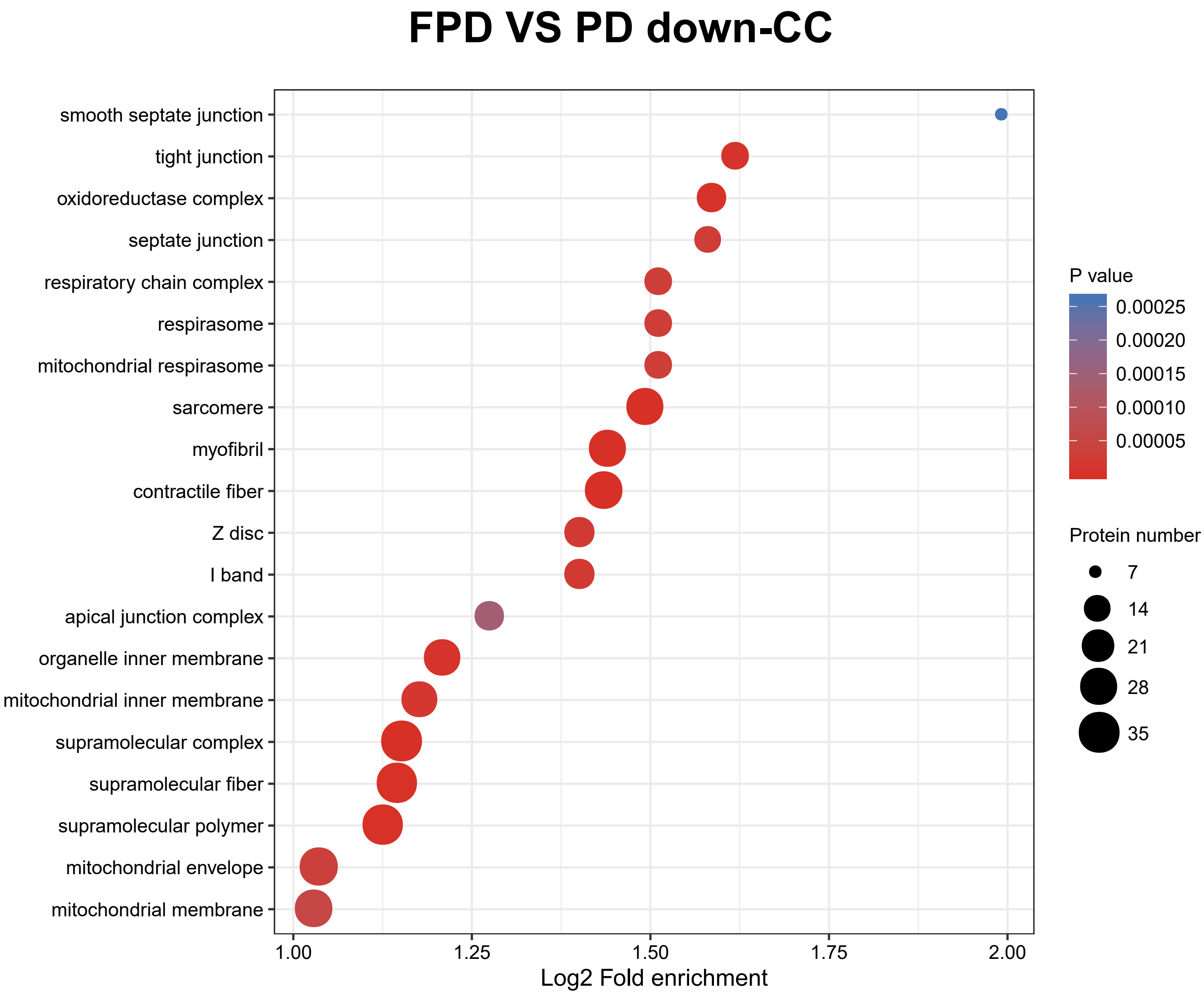

FPD VS PD down-CC
smooth septate junction
tight junction
oxidoreductase complex
septate junction
respiratory chain complex
respirasome
mitochondrial respirasome
sarcomere
myofibril
contractile fiber
Z disc
I band
apical junction complex
organelle inner membrane
mitochondrial inner membrane
supramolecular complex
supramolecular fiber
supramolecular polymer
mitochondrial envelope
mitochondrial membrane
1.00
1.25
1.50
1.75
2.00
Log2 Fold enrichment
P value
0.00025
0.00020
0.00015
0.00010
0.00005
Protein number
7
14
21
28
35

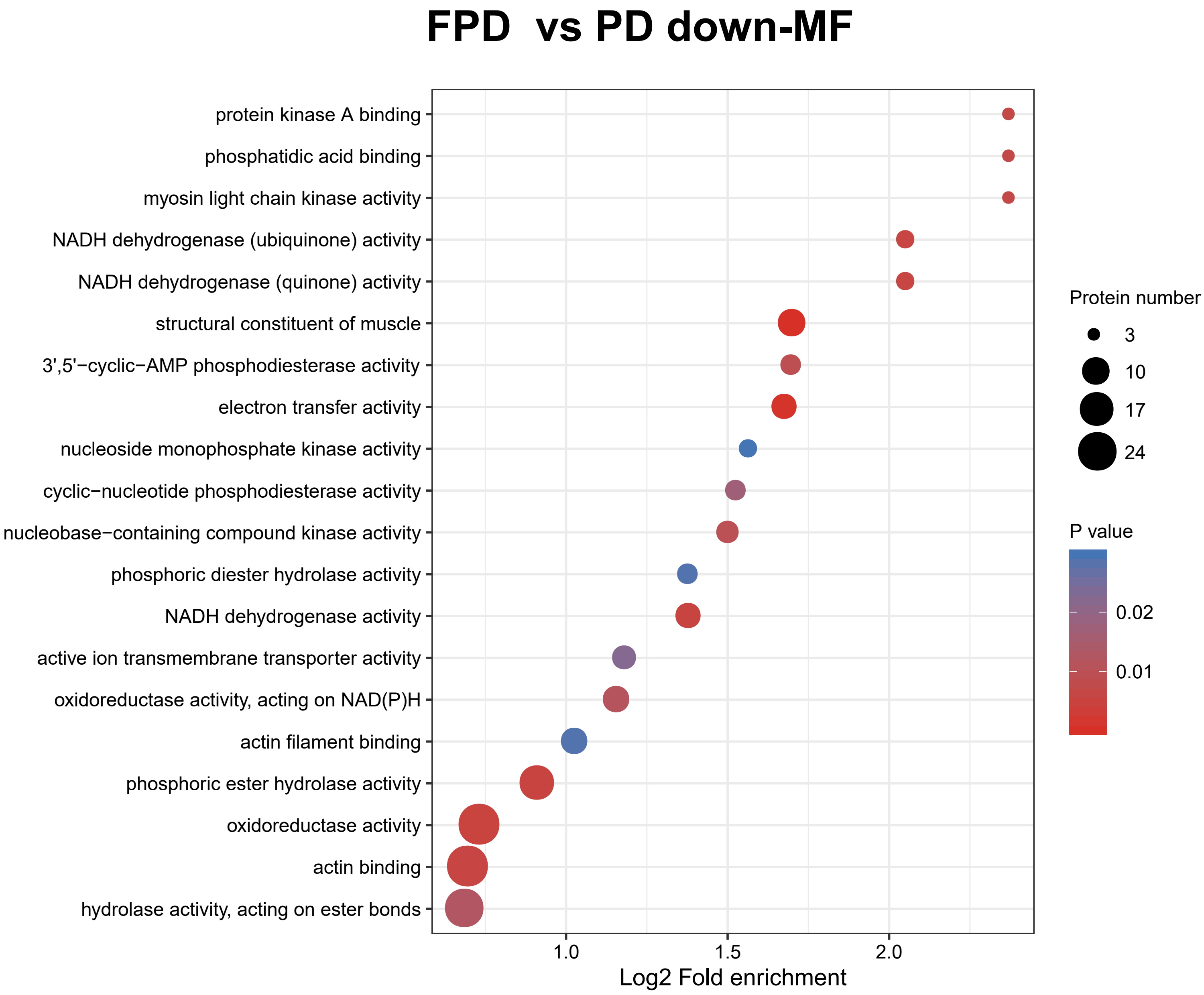

FPD vs PD down-MF
protein kinase A binding
phosphatidic acid binding
myosin light chain kinase activity
NADH dehydrogenase (ubiquinone) activity
NADH dehydrogenase (quinone) activity
structural constituent of muscle
3',5'−cyclic−AMP phosphodiesterase activity
electron transfer activity
nucleoside monophosphate kinase activity
cyclic−nucleotide phosphodiesterase activity
nucleobase−containing compound kinase activity
phosphoric diester hydrolase activity
NADH dehydrogenase activity
active ion transmembrane transporter activity
oxidoreductase activity, acting on NAD(P)H
actin filament binding
phosphoric ester hydrolase activity
oxidoreductase activity
actin binding
hydrolase activity, acting on ester bonds
1.0
1.5
2.0
Log2 Fold enrichment
Protein number
3
10
17
24
P value
0.02
0.01

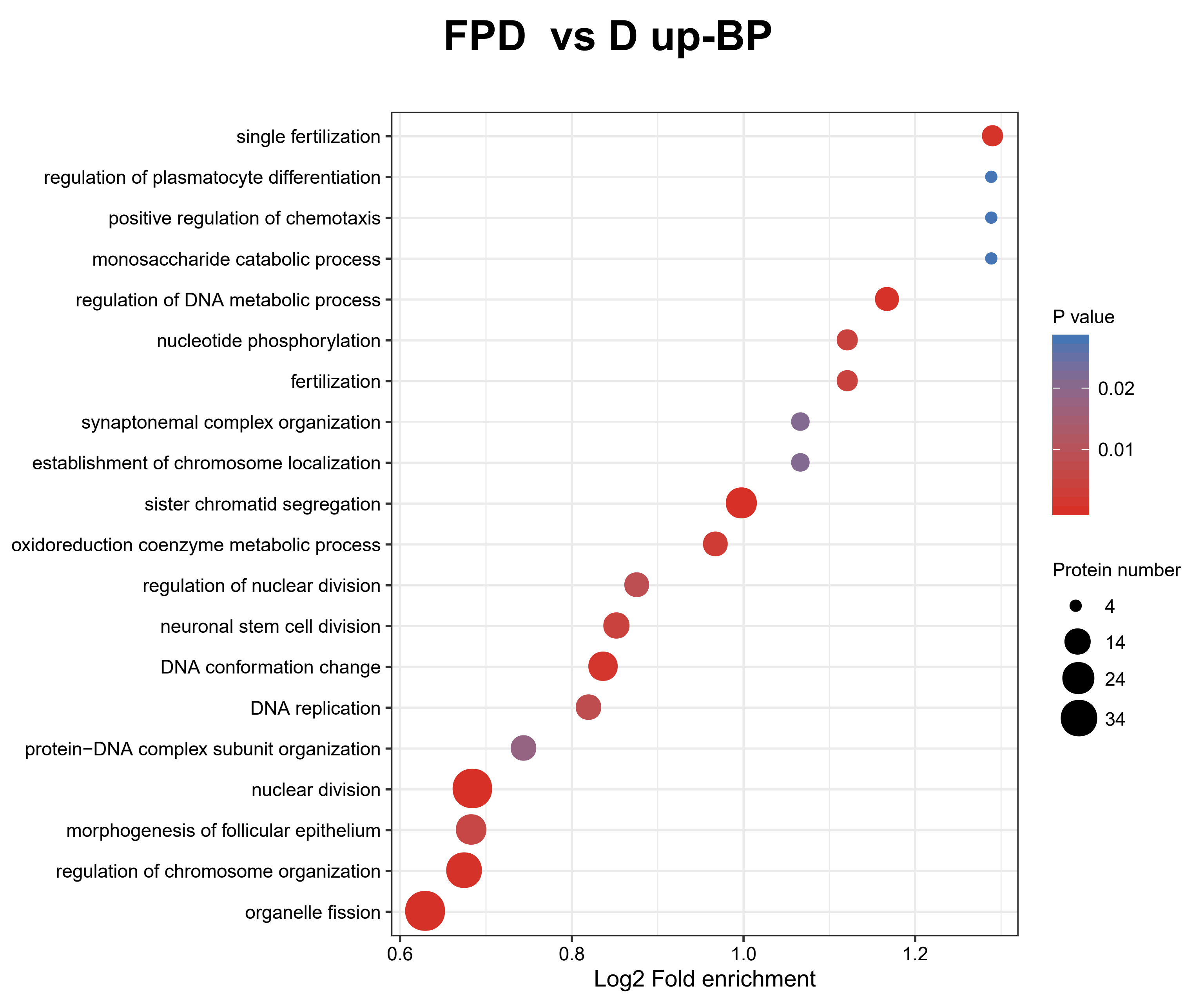
FPD vs D up-BP
single fertilization
regulation of plasmatocyte differentiation
positive regulation of chemotaxis
monosaccharide catabolic process
regulation of DNA metabolic process
nucleotide phosphorylation
fertilization
synaptonemal complex organization
establishment of chromosome localization
sister chromatid segregation
oxidoreduction coenzyme metabolic process
regulation of nuclear division
neuronal stem cell division
DNA conformation change
DNA replication
protein−DNA complex subunit organization
nuclear division
morphogenesis of follicular epithelium
regulation of chromosome organization
organelle fission
0.6
0.8
1.0
1.2
Log2 Fold enrichment
P value
0.02
0.01
Protein number
4
14
24
34

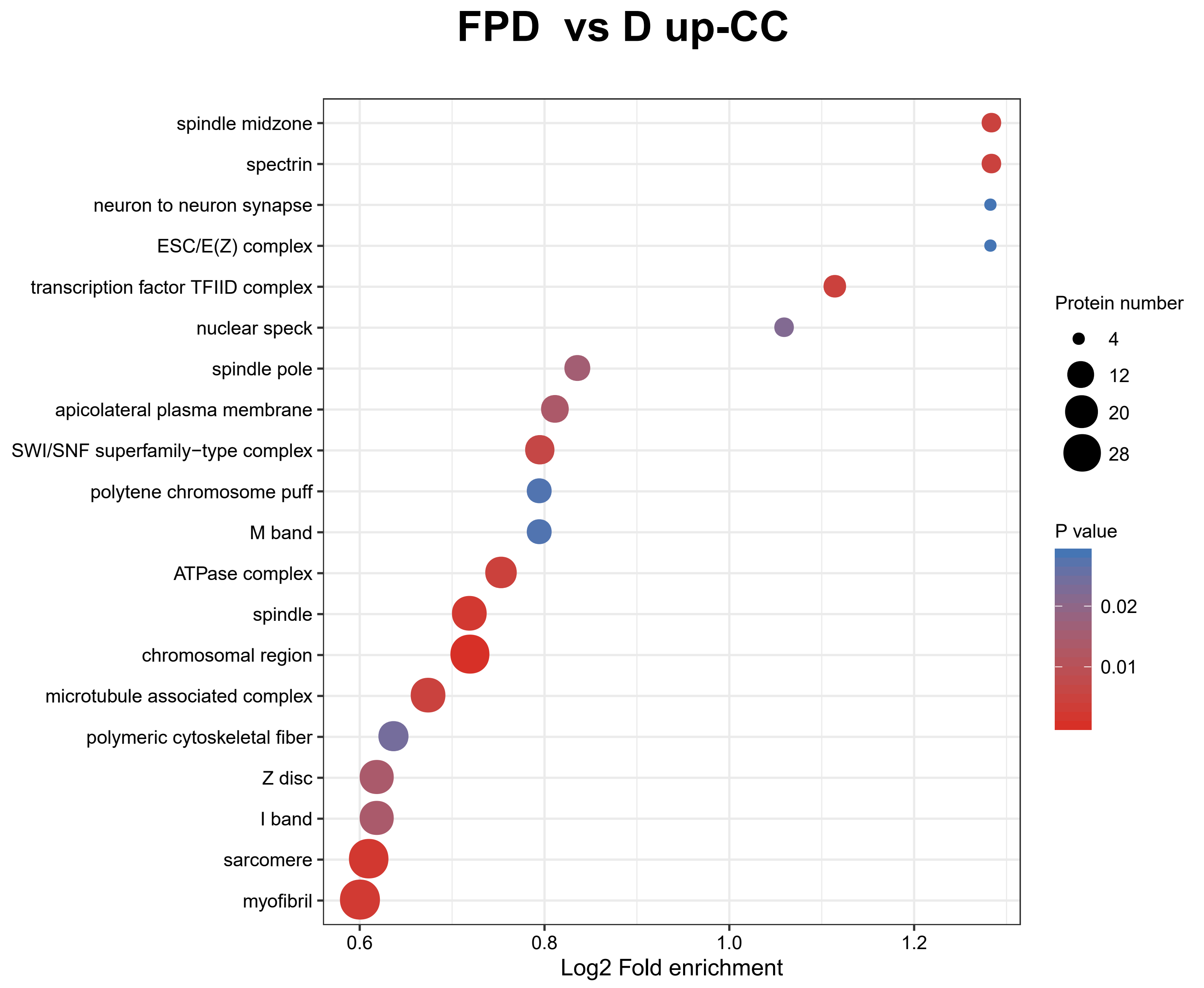
FPD vs D up-CC
spindle midzone
spectrin
neuron to neuron synapse
ESC/E(Z) complex
transcription factor TFIID complex
nuclear speck
spindle pole
apicolateral plasma membrane
SWI/SNF superfamily−type complex
polytene chromosome puff
M band
ATPase complex
spindle
chromosomal region
microtubule associated complex
polymeric cytoskeletal fiber
Z disc
I band
sarcomere
myofibril
0.6
0.8
1.0
1.2
Log2 Fold enrichment
Protein number
4
12
20
28
P value
0.02
0.01

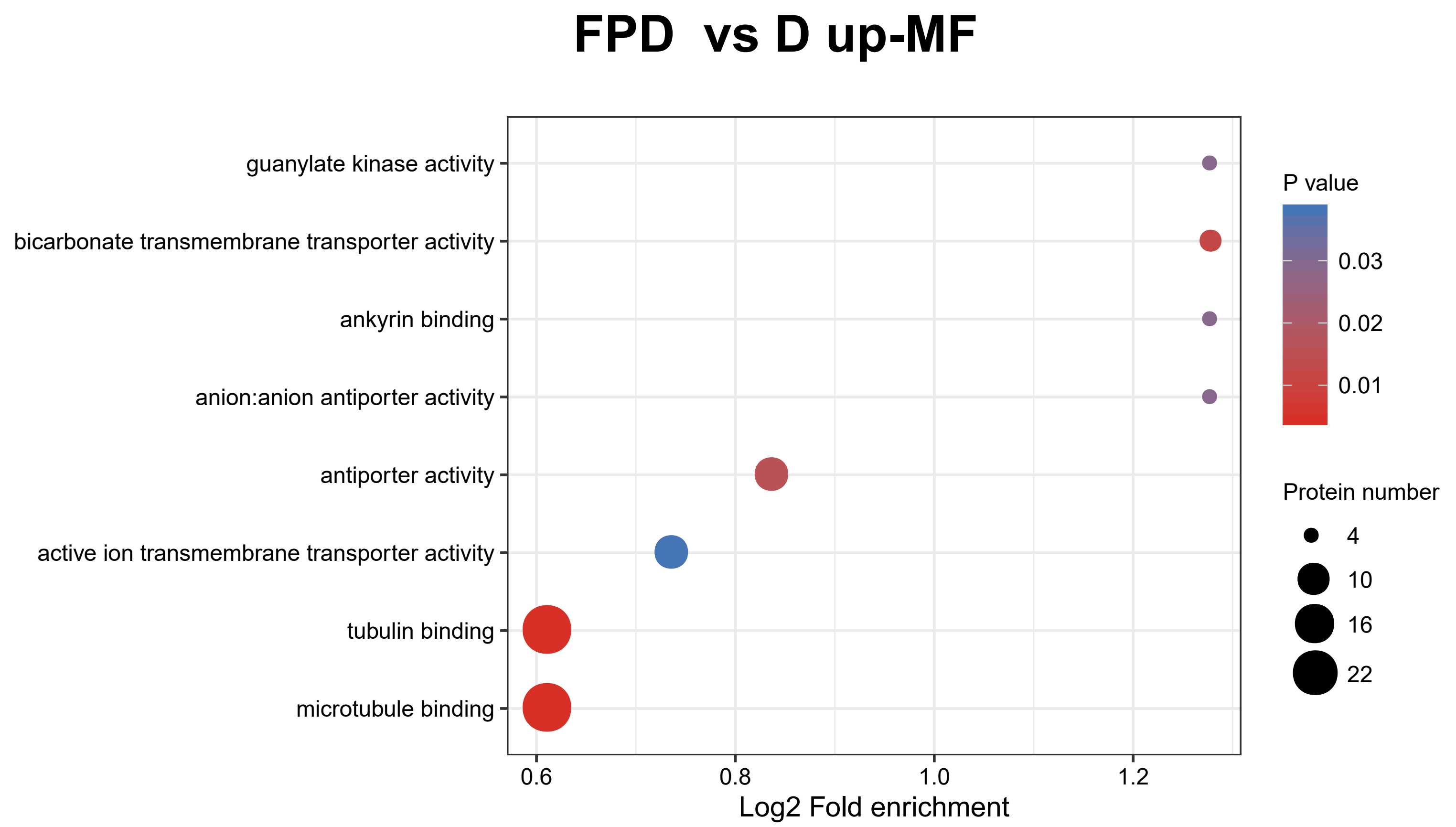
FPD vs D up-MF
guanylate kinase activity
bicarbonate transmembrane transporter activity
ankyrin binding
anion:anion antiporter activity
antiporter activity
active ion transmembrane transporter activity
tubulin binding
microtubule binding
0.6
0.8
1.0
1.2
Log2 Fold enrichment
P value
0.03
0.02
0.01
Protein number
4
10
16
22

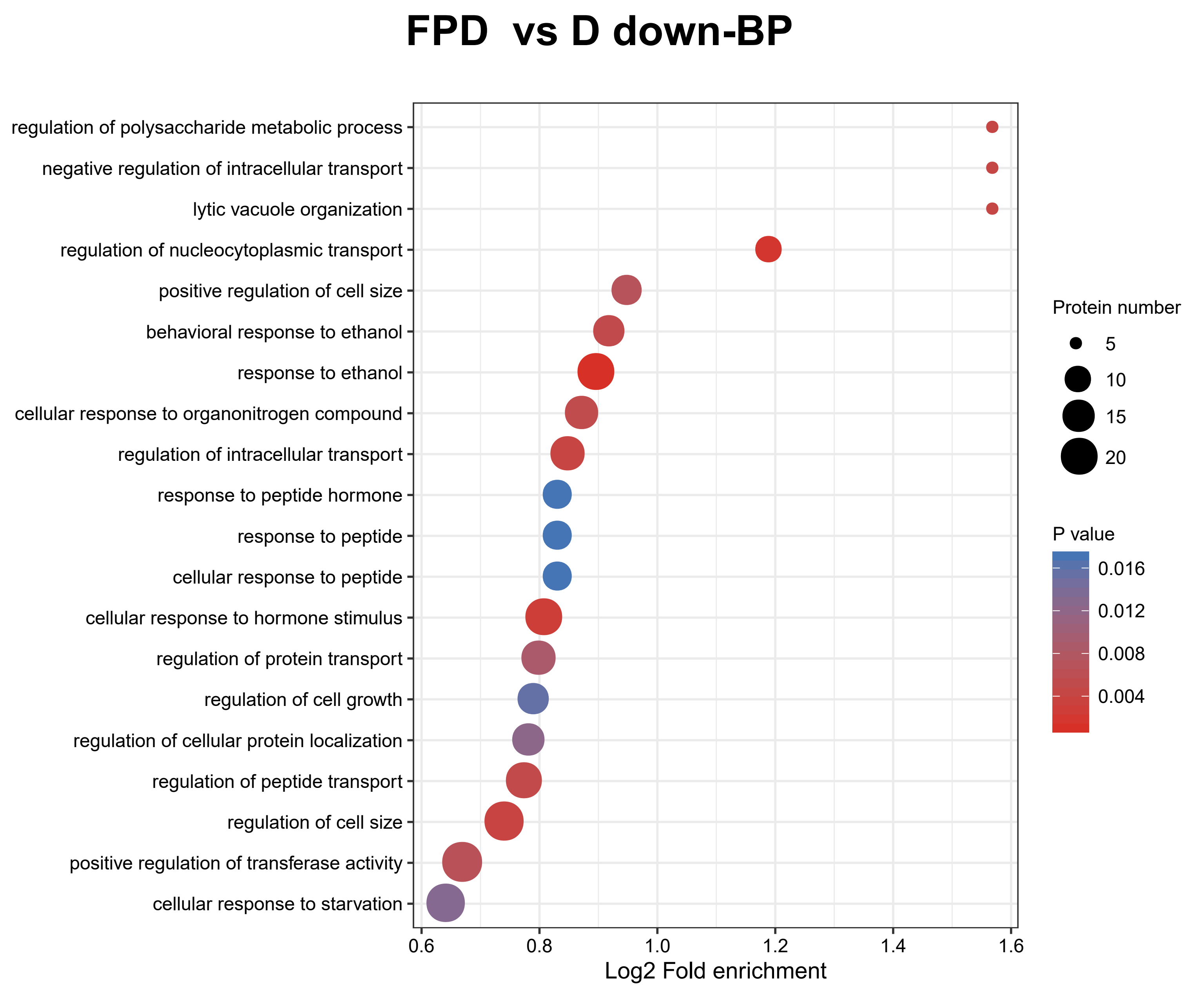
FPD vs D down-BP
regulation of polysaccharide metabolic process
negative regulation of intracellular transport
lytic vacuole organization
regulation of nucleocytoplasmic transport
positive regulation of cell size
behavioral response to ethanol
response to ethanol
cellular response to organonitrogen compound
regulation of intracellular transport
response to peptide hormone
response to peptide
cellular response to peptide
cellular response to hormone stimulus
regulation of protein transport
regulation of cell growth
regulation of cellular protein localization
regulation of peptide transport
regulation of cell size
positive regulation of transferase activity
cellular response to starvation
0.6
0.8
1.0
1.2
1.4
1.6
Log2 Fold enrichment
Protein number
5
10
15
20
P value
0.016
0.012
0.008
0.004

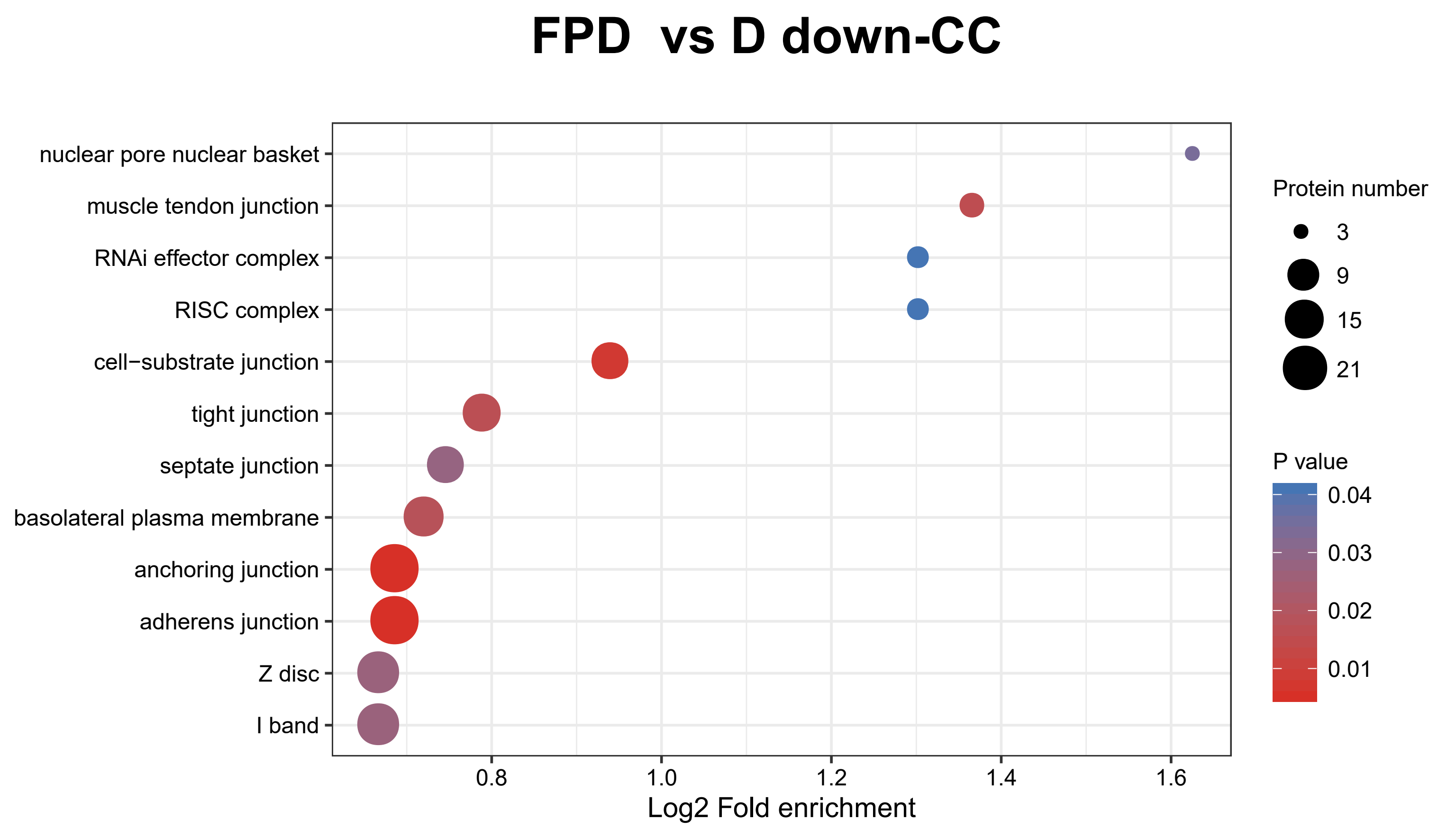
FPD vs D down-CC
nuclear pore nuclear basket
muscle tendon junction
RNAi effector complex
RISC complex
cell−substrate junction
tight junction
septate junction
basolateral plasma membrane
anchoring junction
adherens junction
Z disc
I band
0.8
1.0
1.2
1.4
1.6
Log2 Fold enrichment
Protein number
3
9
15
21
P value
0.04
0.03
0.02
0.01

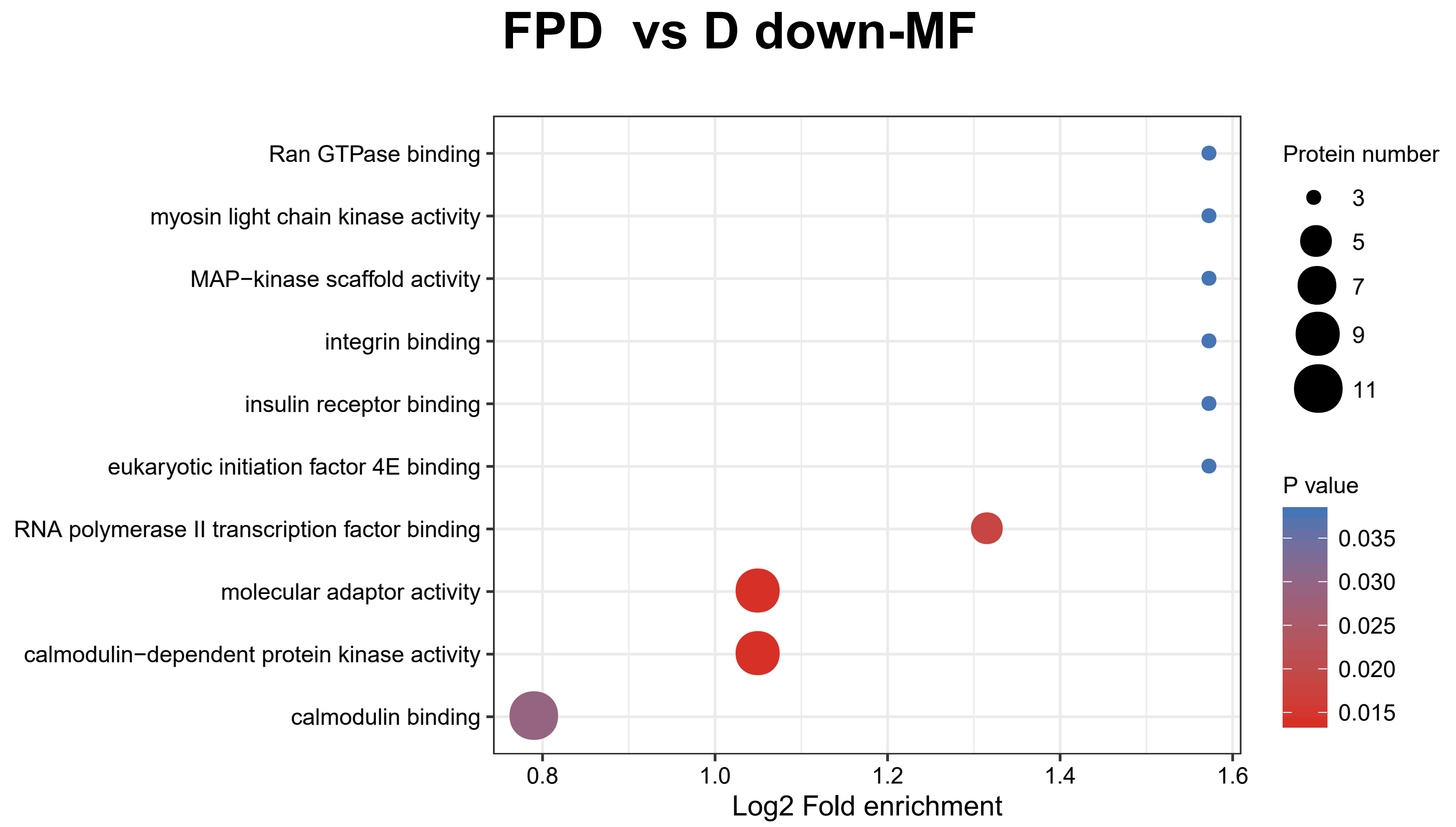
FPD vs D down-MF
Ran GTPase binding
myosin light chain kinase activity
MAP−kinase scaffold activity
integrin binding
insulin receptor binding
eukaryotic initiation factor 4E binding
RNA polymerase II transcription factor binding
molecular adaptor activity
calmodulin−dependent protein kinase activity
calmodulin binding
0.8
1.0
1.2
1.4
1.6
Log2 Fold enrichment
Protein number
3
5
7
9
11
P value
0.035
0.030
0.025
0.020
0.015
