# Supplementary material for: Integrative Proteomic and Phosphoproteomic Analyses Revealed Complex Mechanisms Underlying Reproductive Diapause in Bombus terrestris Queens
Source: Insects. 2022 Sep 23;13(10):862. doi: 10.3390/insects13100862 (PMC9604461; doi:10.3390/insects13100862)

Supplementary Figure S11: KEGG/GO/Domain analyses of six clusters in phosphoproteomic Mfuzz analysis. (A) KEGG enrichment analysis. (B-D) Heat maps obtained from biological process, cellular component and molecular function analyses of six clusters. (E) Protein domain analysis.

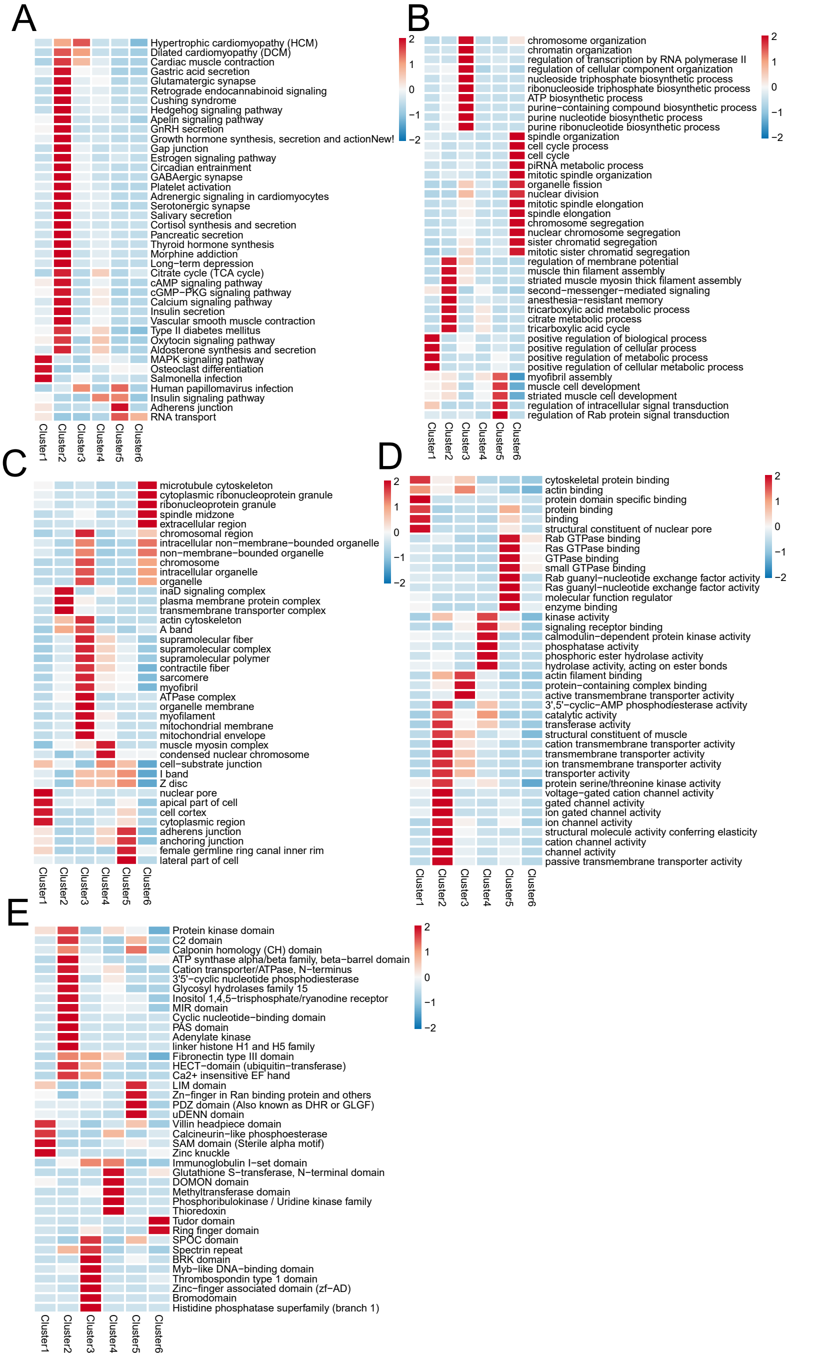

Supplement: Supplementary file 1 [file insects-13-00862-s001.zip › insects-1876268-supplementary/insects-1876268-proofed-supplementary/Figure S11.pdf]
