# Supplementary material for: Integrative Proteomic and Phosphoproteomic Analyses Revealed Complex Mechanisms Underlying Reproductive Diapause in Bombus terrestris Queens
Source: Insects. 2022 Sep 23;13(10):862. doi: 10.3390/insects13100862 (PMC9604461; doi:10.3390/insects13100862)
Supplement: Supplementary file 1 [file insects-13-00862-s001.zip › insects-1876268-supplementary/insects-1876268-proofed-supplementary/Figure S12.pdf]

Supplementary Figure S12: Phosphokinase activity in each group and comparable group.

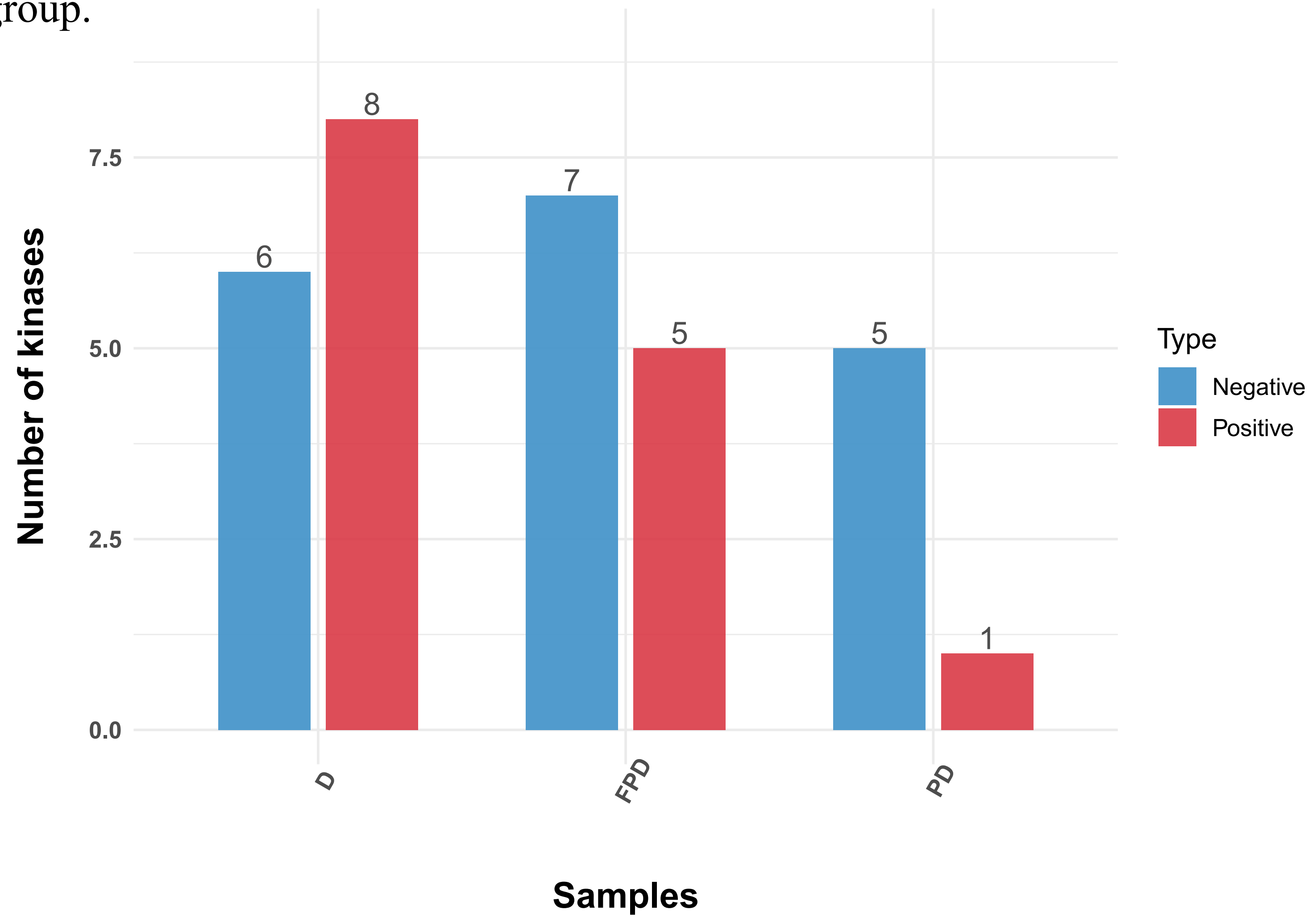

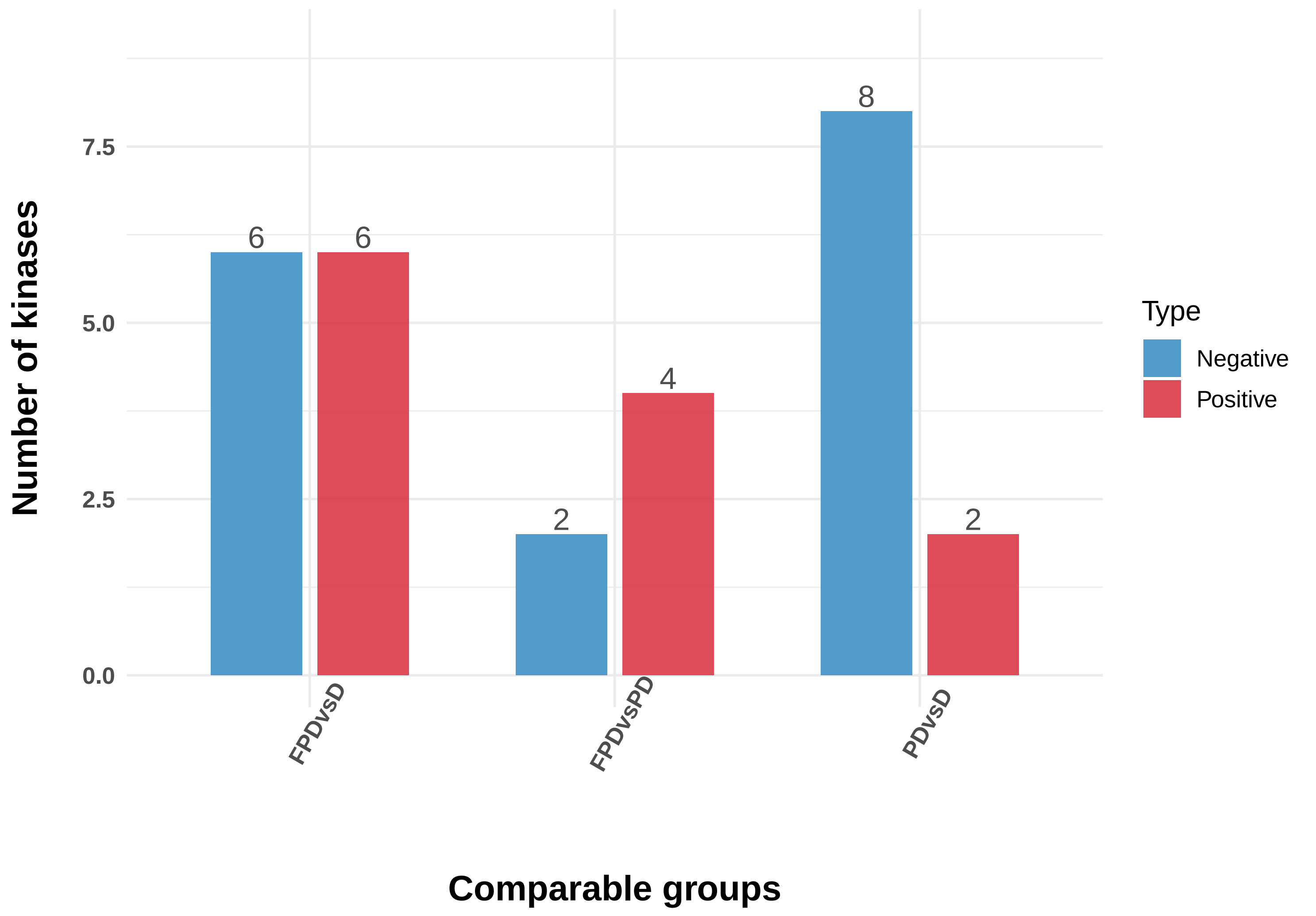

Number of kinases
7.5
5.0
2.5
0.0
6
6
2
4
8
2
FPDvsD
FPDvsPD
PDvsD
Comparable groups
Type
Negative
Positive
