# Supplementary material for: Integrative Proteomic and Phosphoproteomic Analyses Revealed Complex Mechanisms Underlying Reproductive Diapause in Bombus terrestris Queens
Source: Insects. 2022 Sep 23;13(10):862. doi: 10.3390/insects13100862 (PMC9604461; doi:10.3390/insects13100862)

Supplementary Figure S13: Motif enrichment heat map of phosphoserine (S) and phosphothreonine (T) upstream and downstream of the identified phosphorylation modification sites.

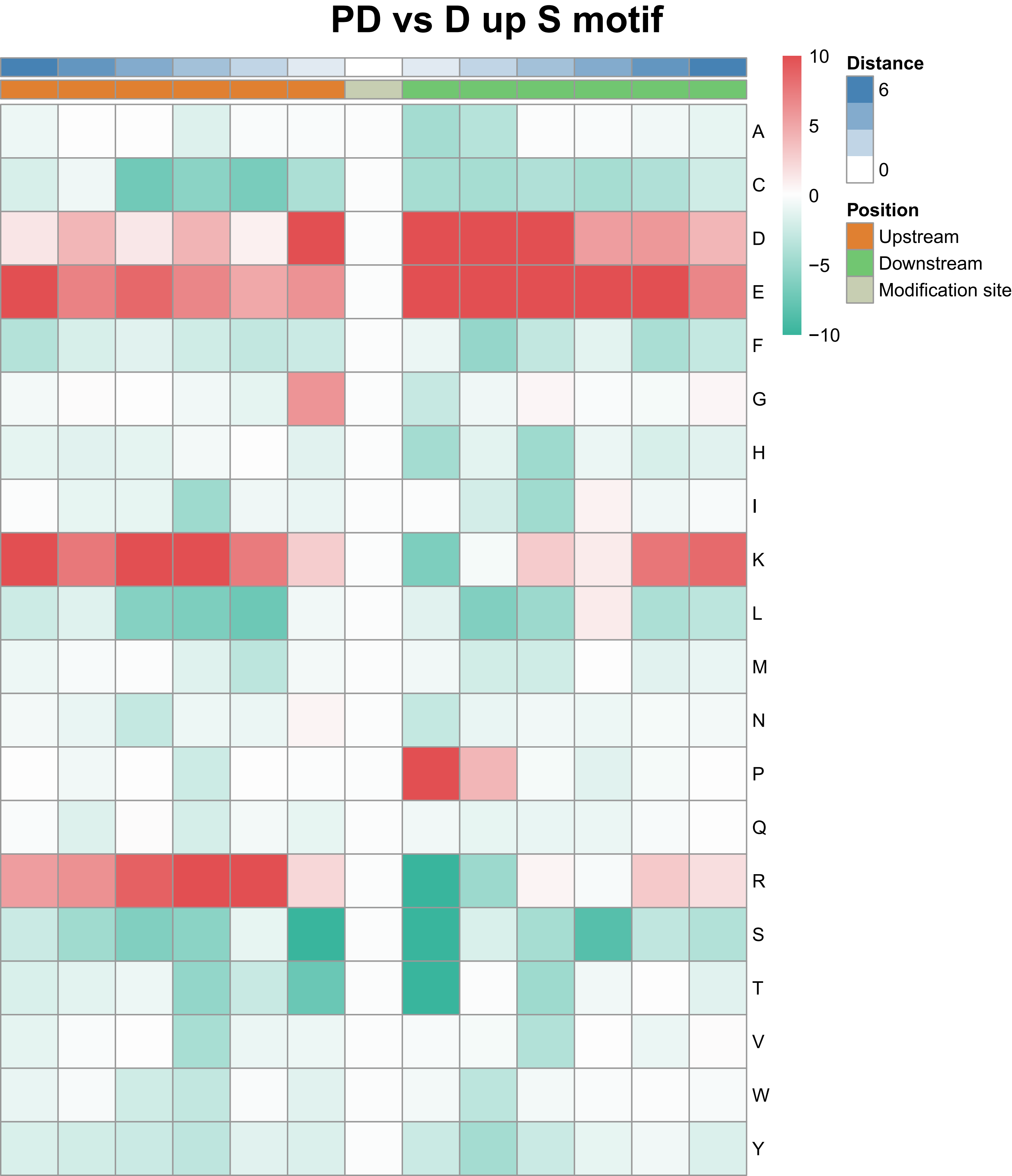

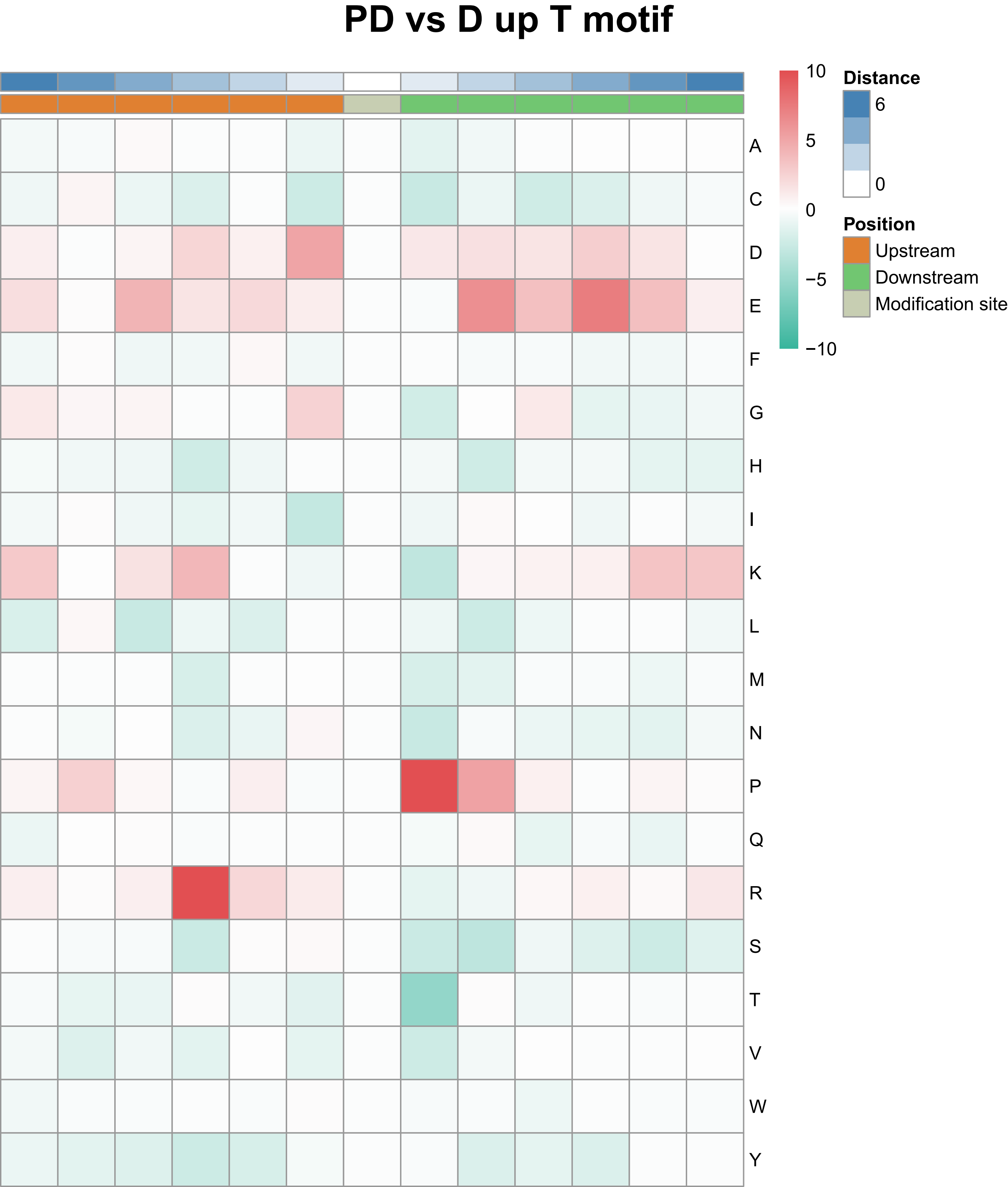
PD vs D up T motif
A
C
D
E
F
G
H
I
K
L
M
N
P
Q
R
S
T
V
W
Y
10
5
0
−5
−10
Distance
6
0
Position
Upstream
Downstream
Modification site

PD vs D down S motif

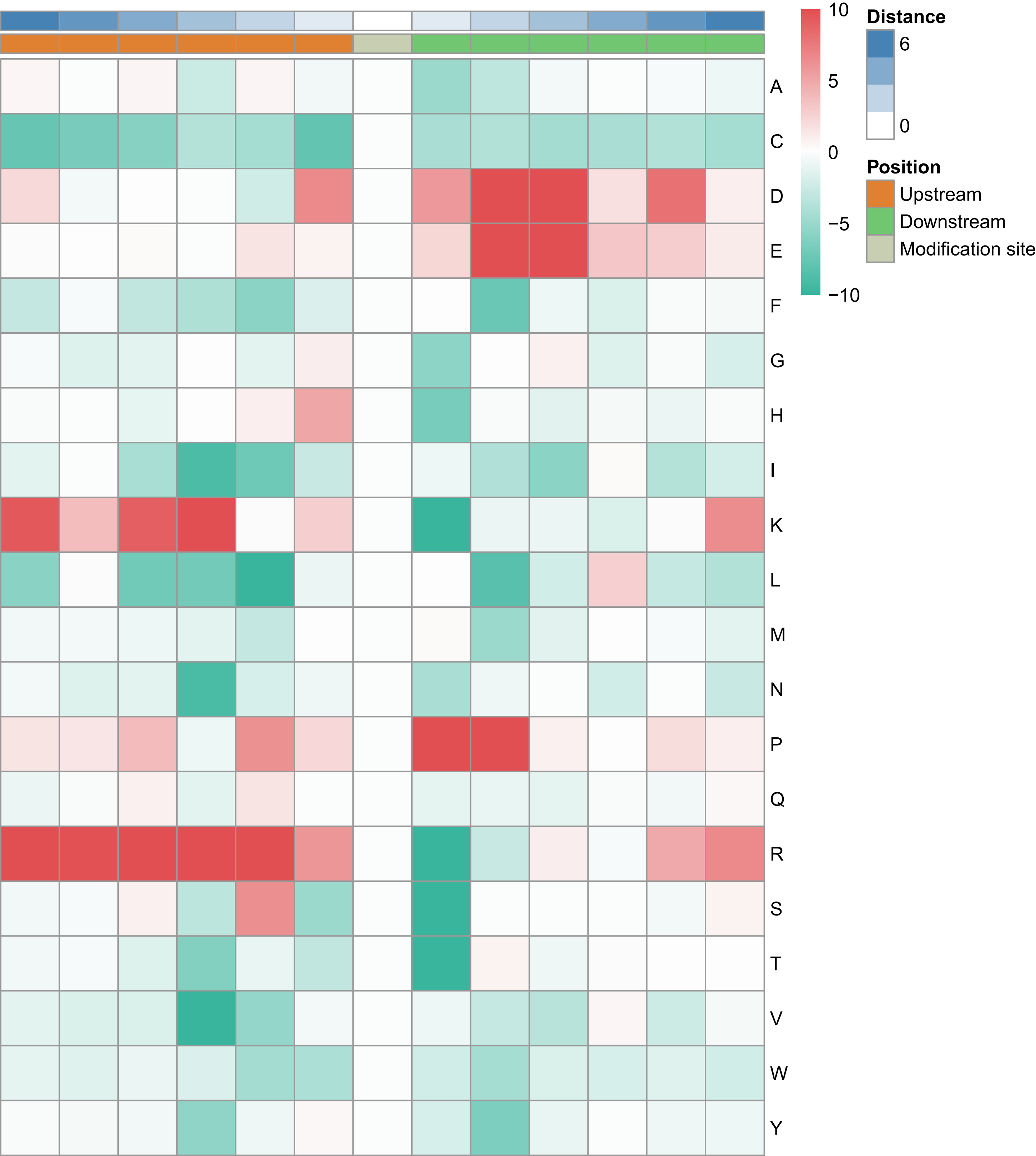

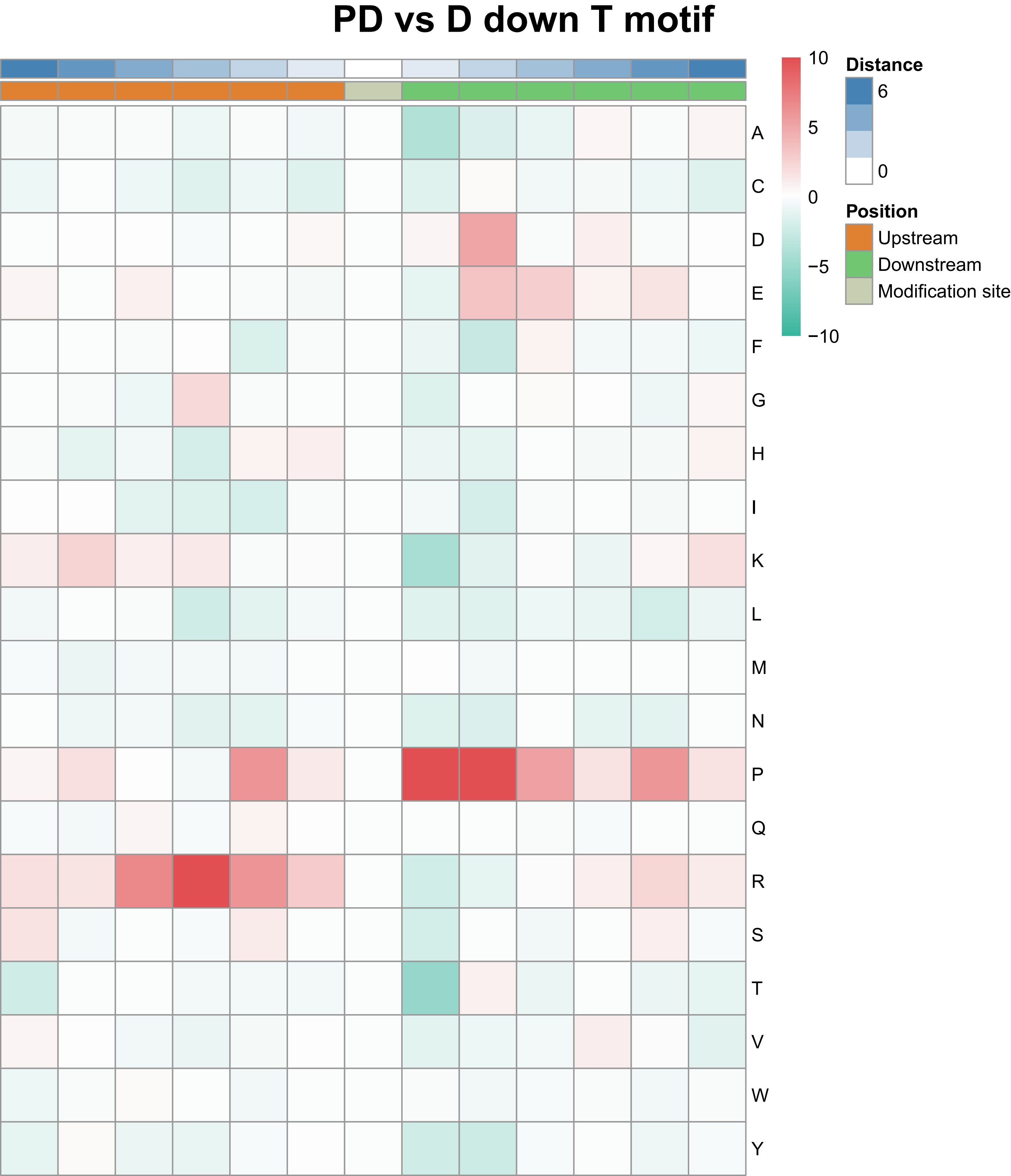

PD vs D down T motif
A
C
D
E
F
G
H
I
K
L
M
N
P
Q
R
S
T
V
W
Y
10
5
0
−5
−10
Distance
6
0
Position
Upstream
Downstream
Modification site

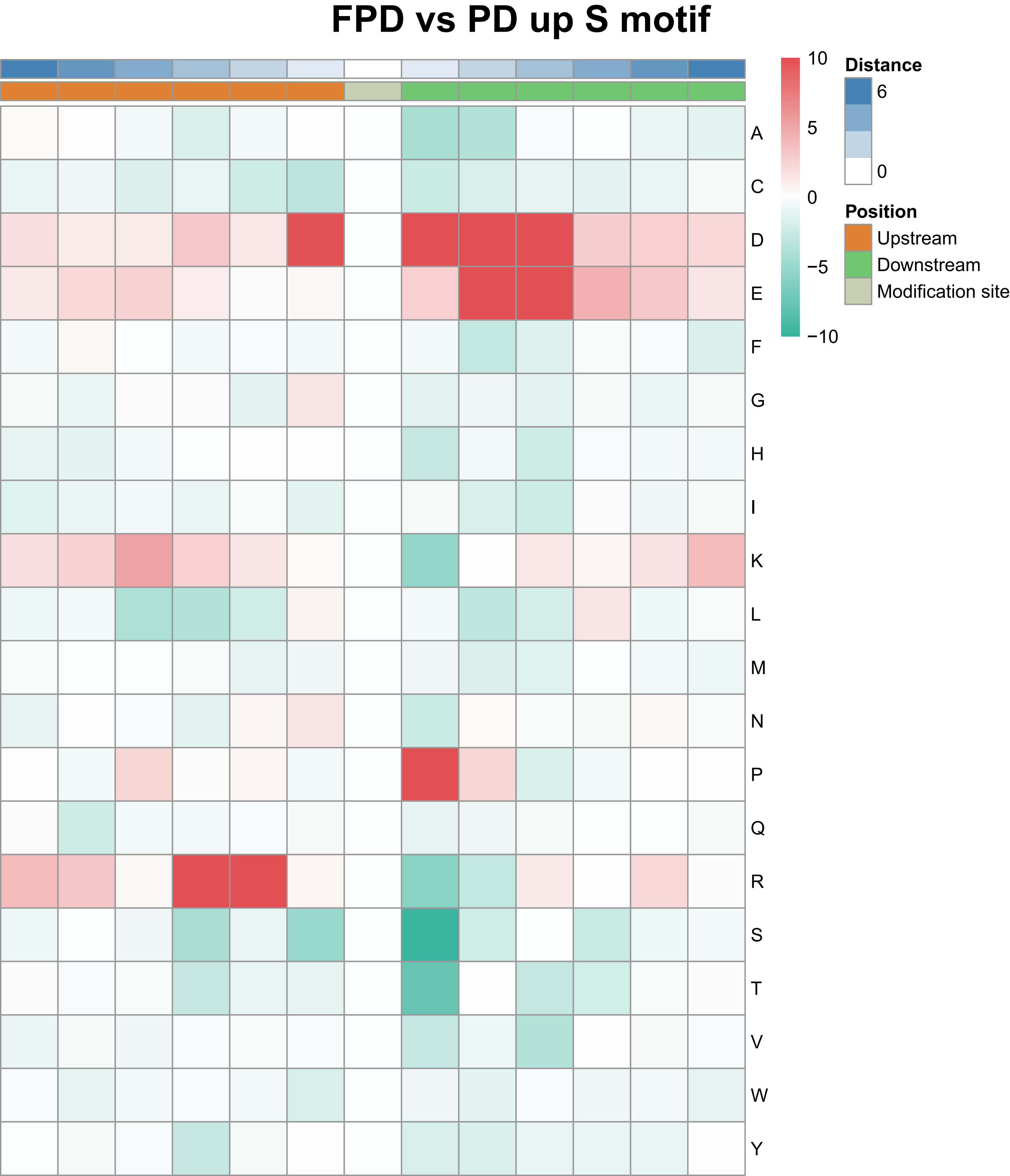
FPD vs PD up S motif
A
C
D
E
F
G
H
I
K
L
M
N
P
Q
R
S
T
V
W
Y
10
5
0
−5
−10
Distance
6
0
Position
Upstream
Downstream
Modification site

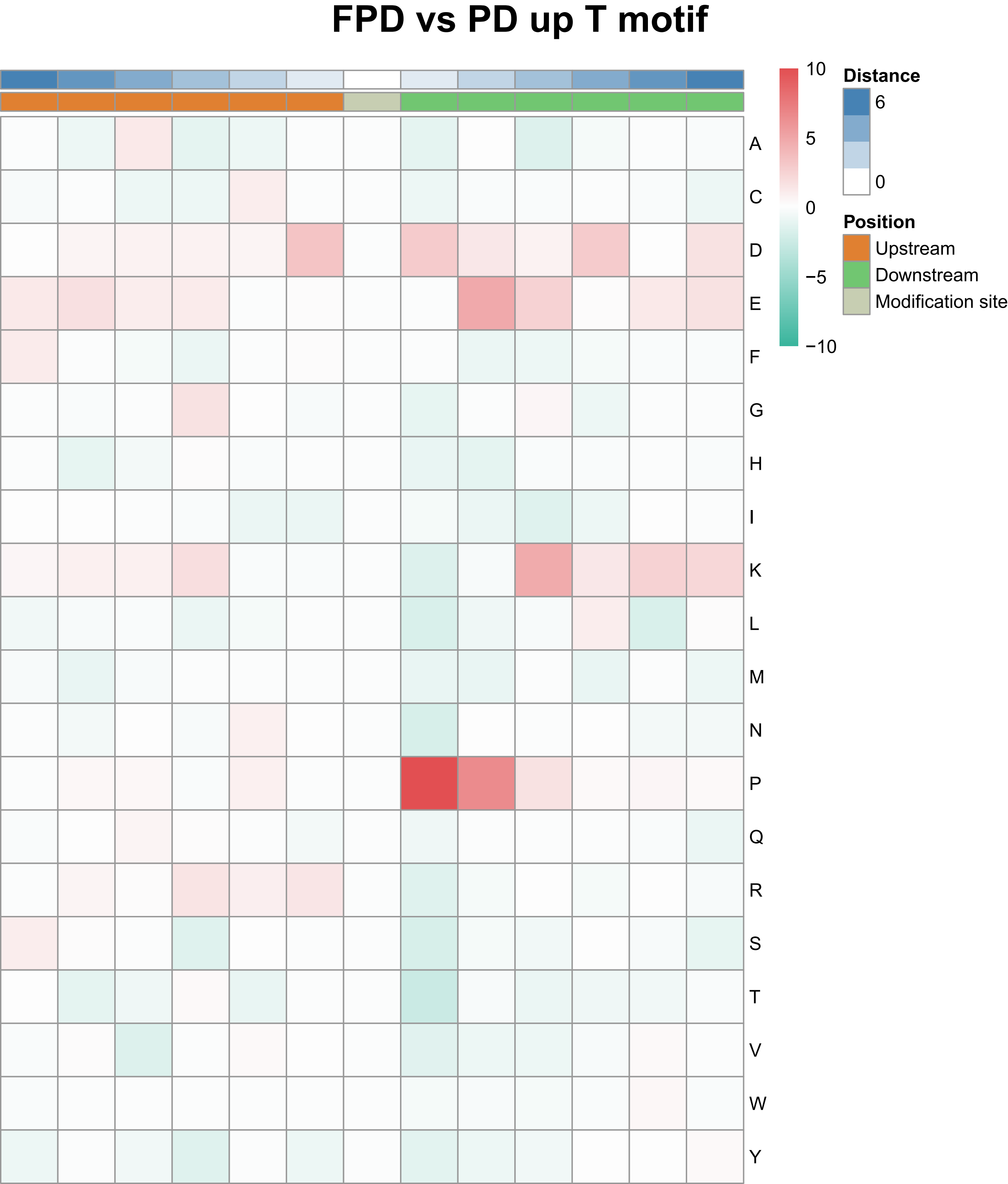
FPD vs PD up T motif
10
5
0
−5
−10
Distance
6
0
Position
Upstream
Downstream
Modification site
A
C
D
E
F
G
H
I
K
L
M
N
P
Q
R
S
T
V
W
Y

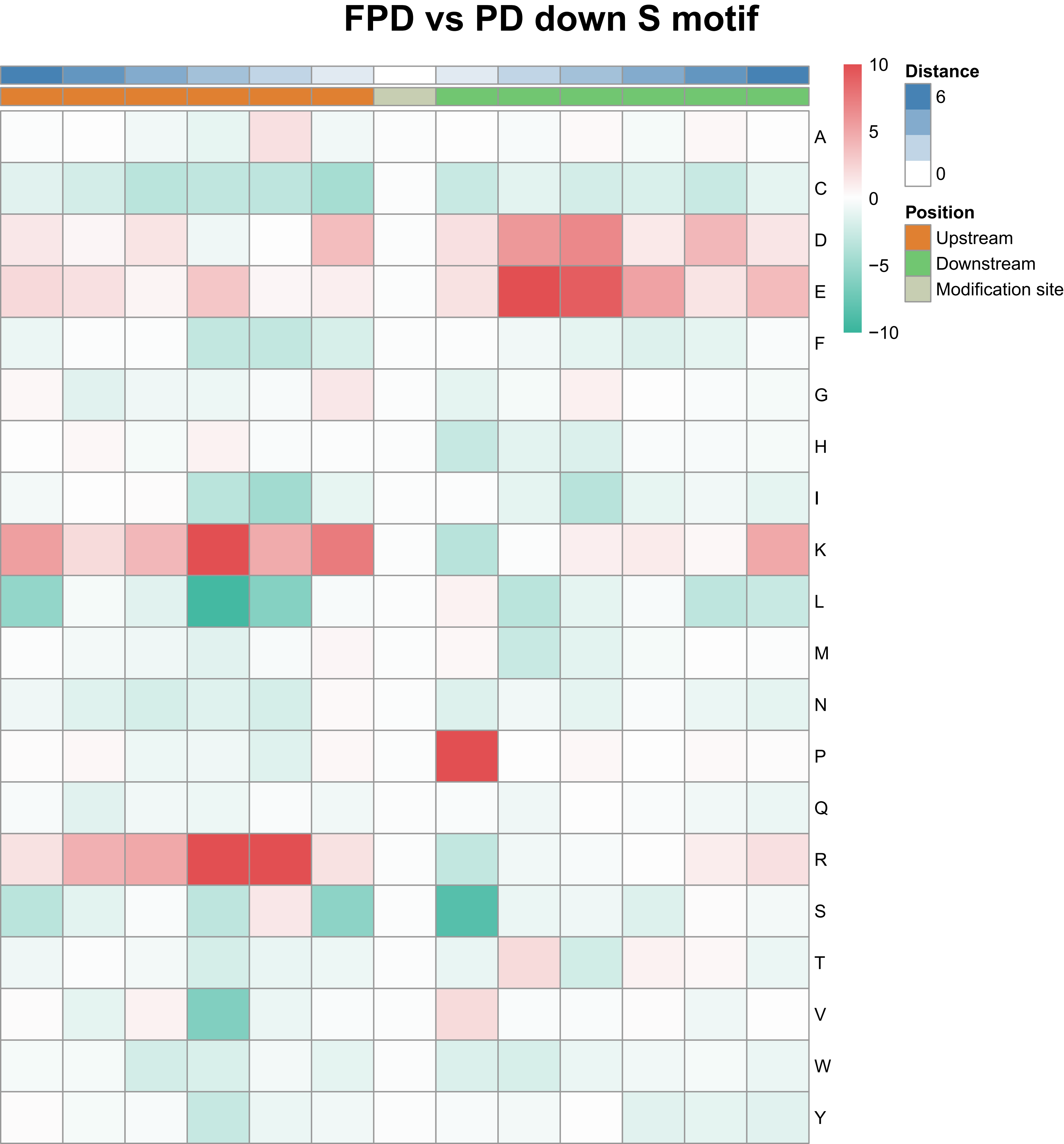
FPD vs PD down S motif
Distance
6
0
Position
Upstream
Downstream
Modification site
10
5
0
−5
−10
A
C
D
E
F
G
H
I
K
L
M
N
P
Q
R
S
T
V
W
Y

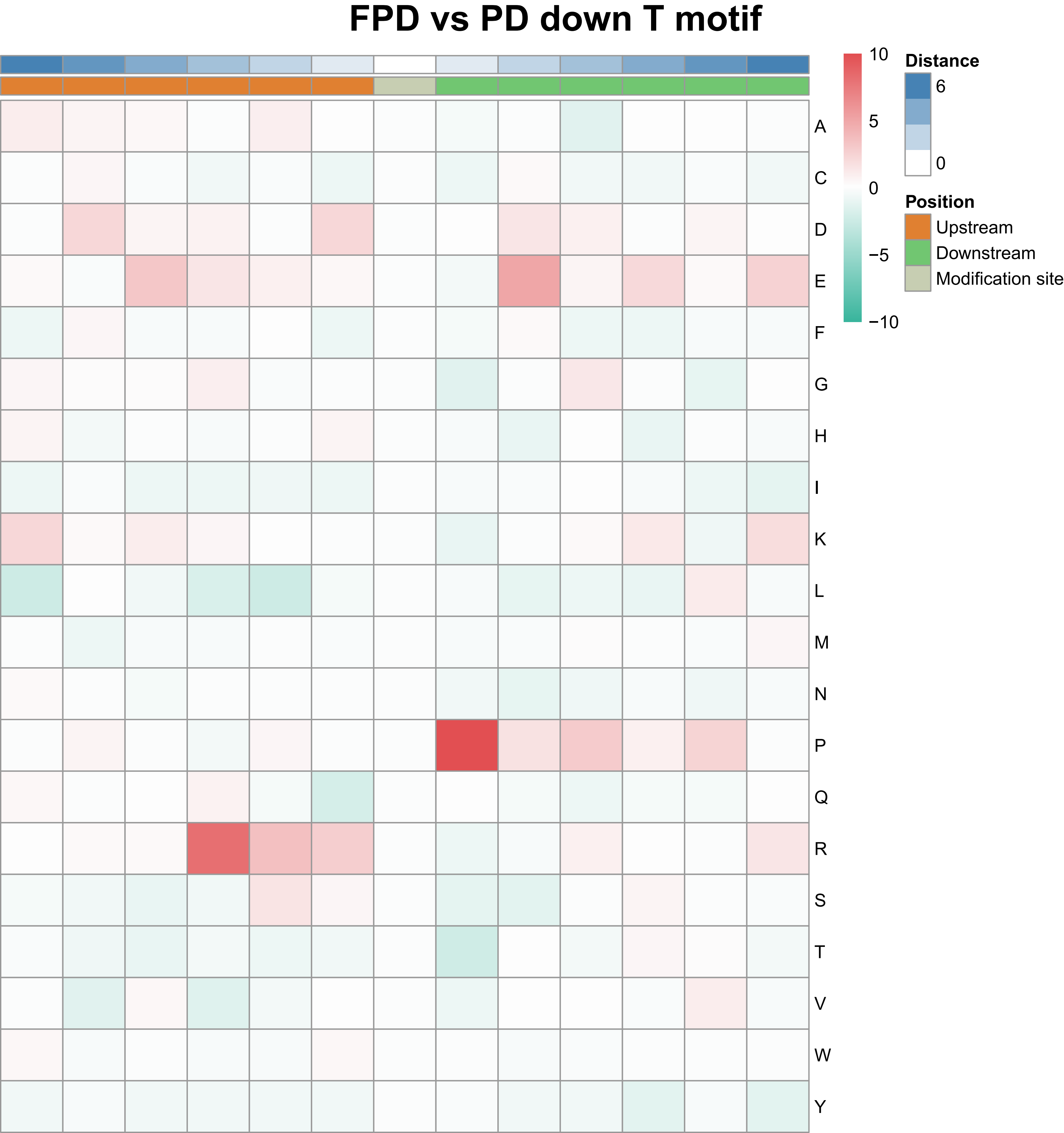

FPD vs PD down T motif
A
C
D
E
F
G
H
I
K
L
M
N
P
Q
R
S
T
V
W
Y
10
5
0
−5
−10
Distance
6
0
Position
Upstream
Downstream
Modification site

FPD vs D up S motif

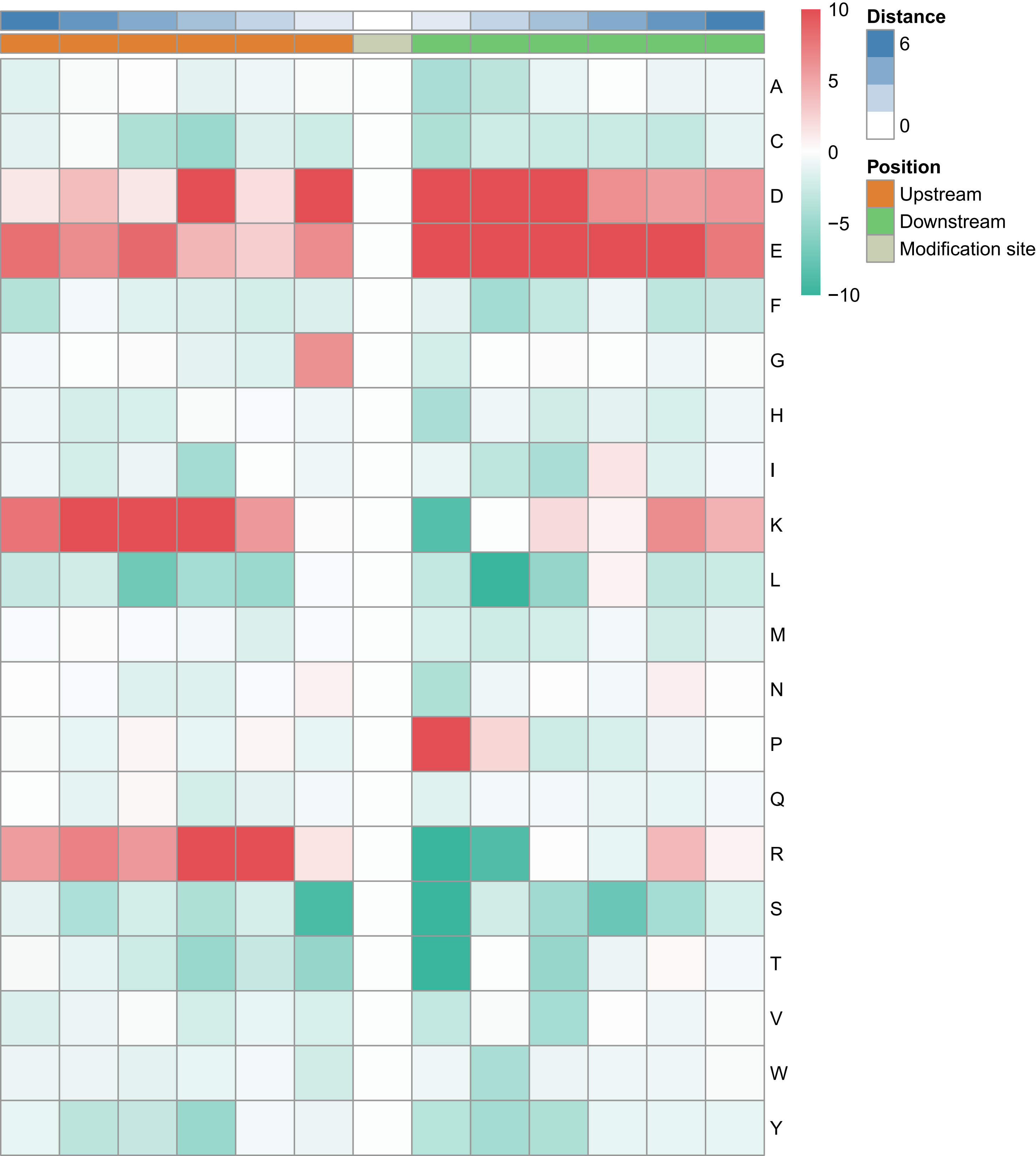

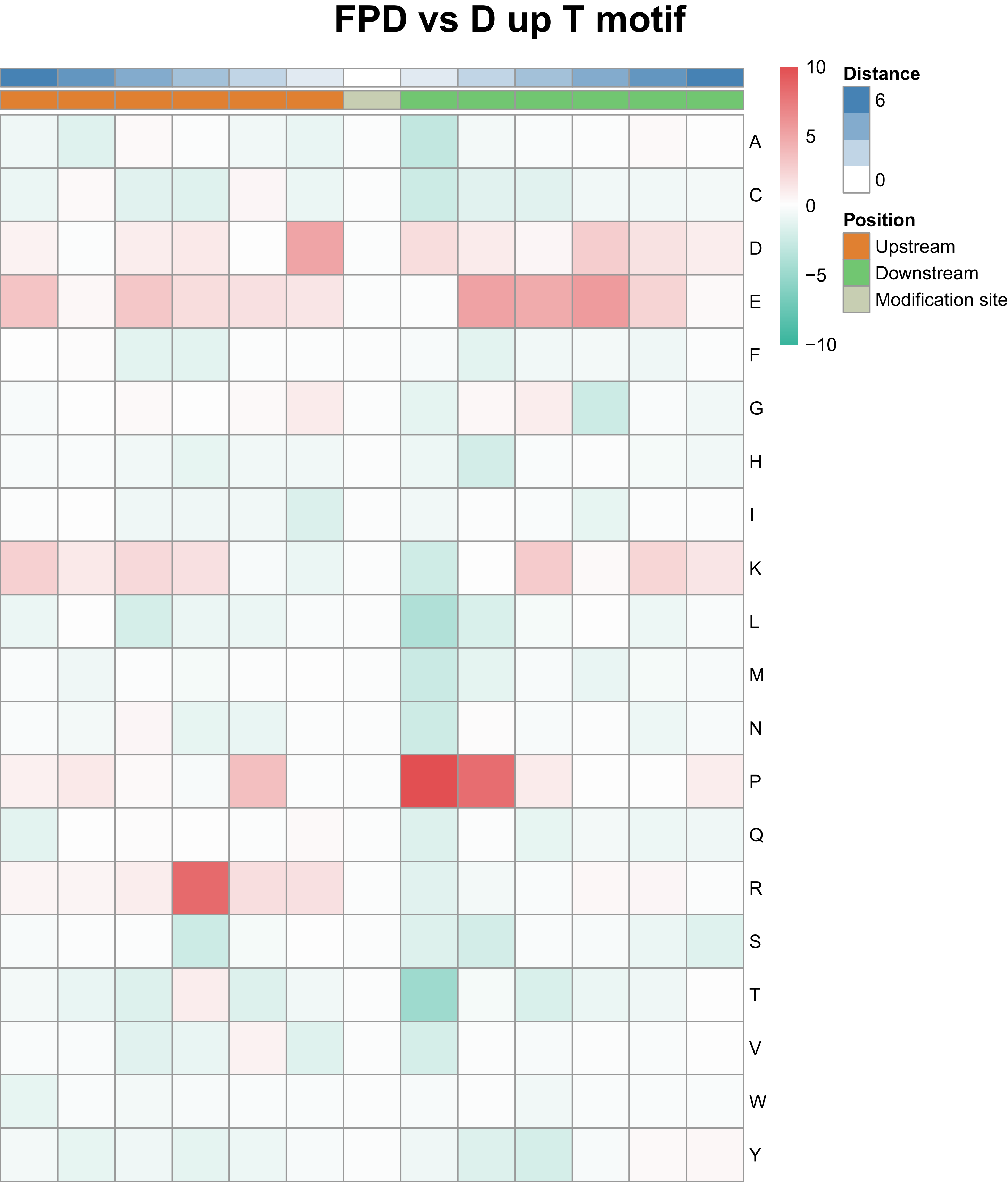
FPD vs D up T motif
A
C
D
E
F
G
H
I
K
L
M
N
P
Q
R
S
T
V
W
Y
10
5
0
−5
−10
Distance
6
0
Position
Upstream
Downstream
Modification site

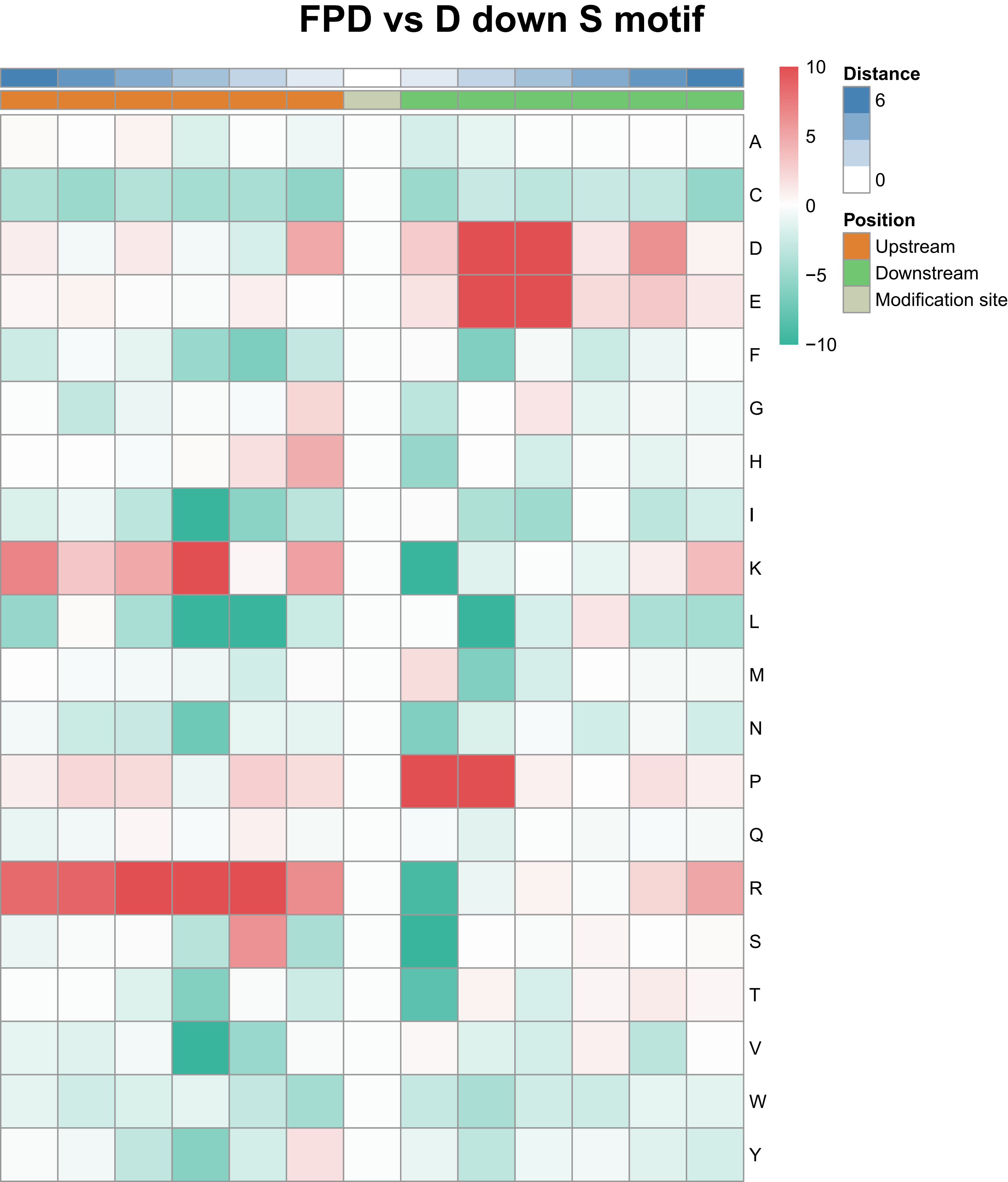
FPD vs D down S motif
A
C
D
E
F
G
H
I
K
L
M
N
P
Q
R
S
T
V
W
Y
10
5
0
−5
−10
Distance
6
0
Position
Upstream
Downstream
Modification site

FPD vs D down T motif

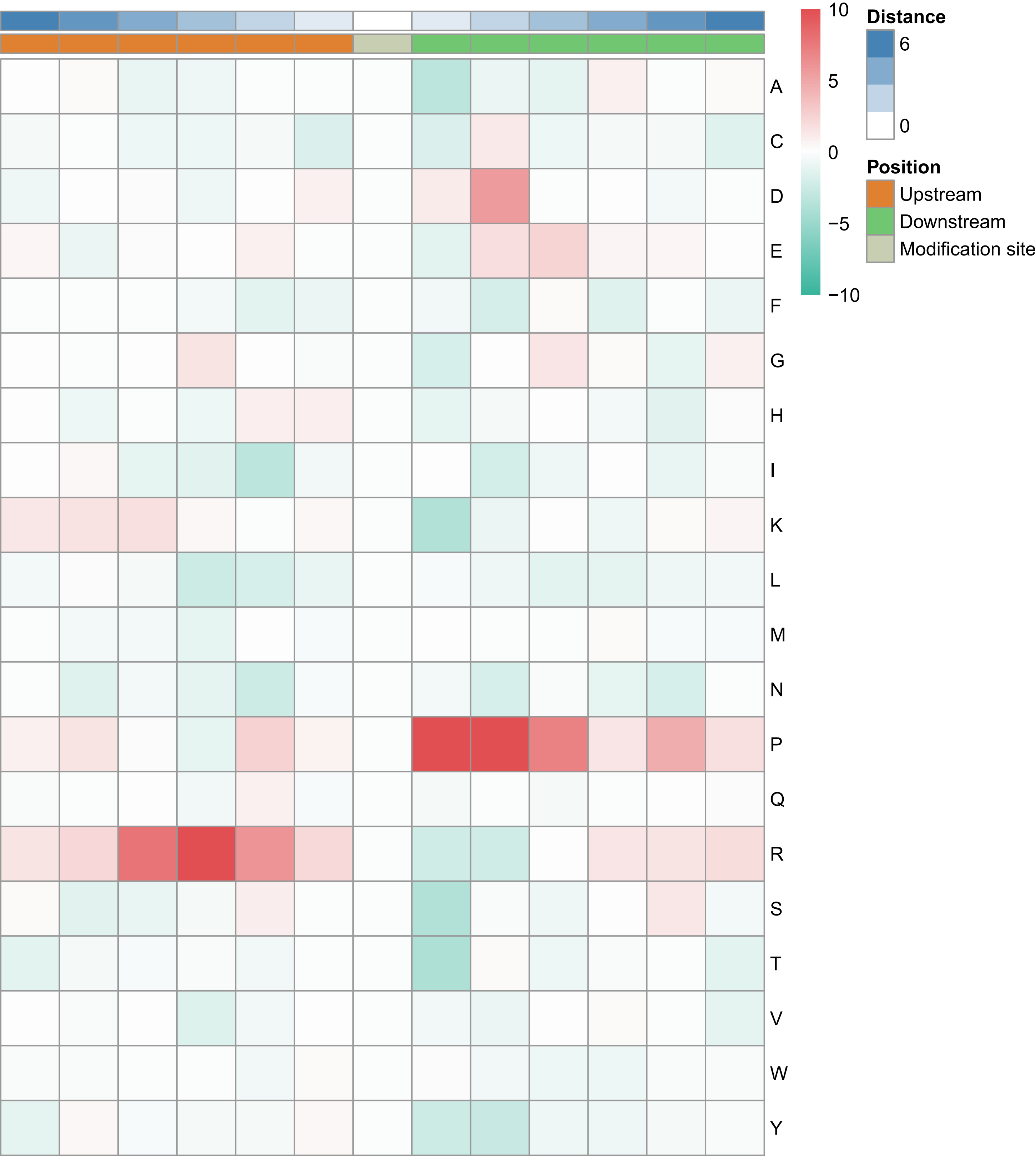

Supplement: Supplementary file 1 [file insects-13-00862-s001.zip › insects-1876268-supplementary/insects-1876268-proofed-supplementary/Figure S13.pdf]
