# Supplementary material for: Integrative Proteomic and Phosphoproteomic Analyses Revealed Complex Mechanisms Underlying Reproductive Diapause in Bombus terrestris Queens
Source: Insects. 2022 Sep 23;13(10):862. doi: 10.3390/insects13100862 (PMC9604461; doi:10.3390/insects13100862)

Supplementary Figure S14: Analyses of the protein-protein interaction networks for differentially expressed proteins (A, B) and differentially expressed phosphorylated proteins (C, D) between FPD/PD and FPD/D, respectively.

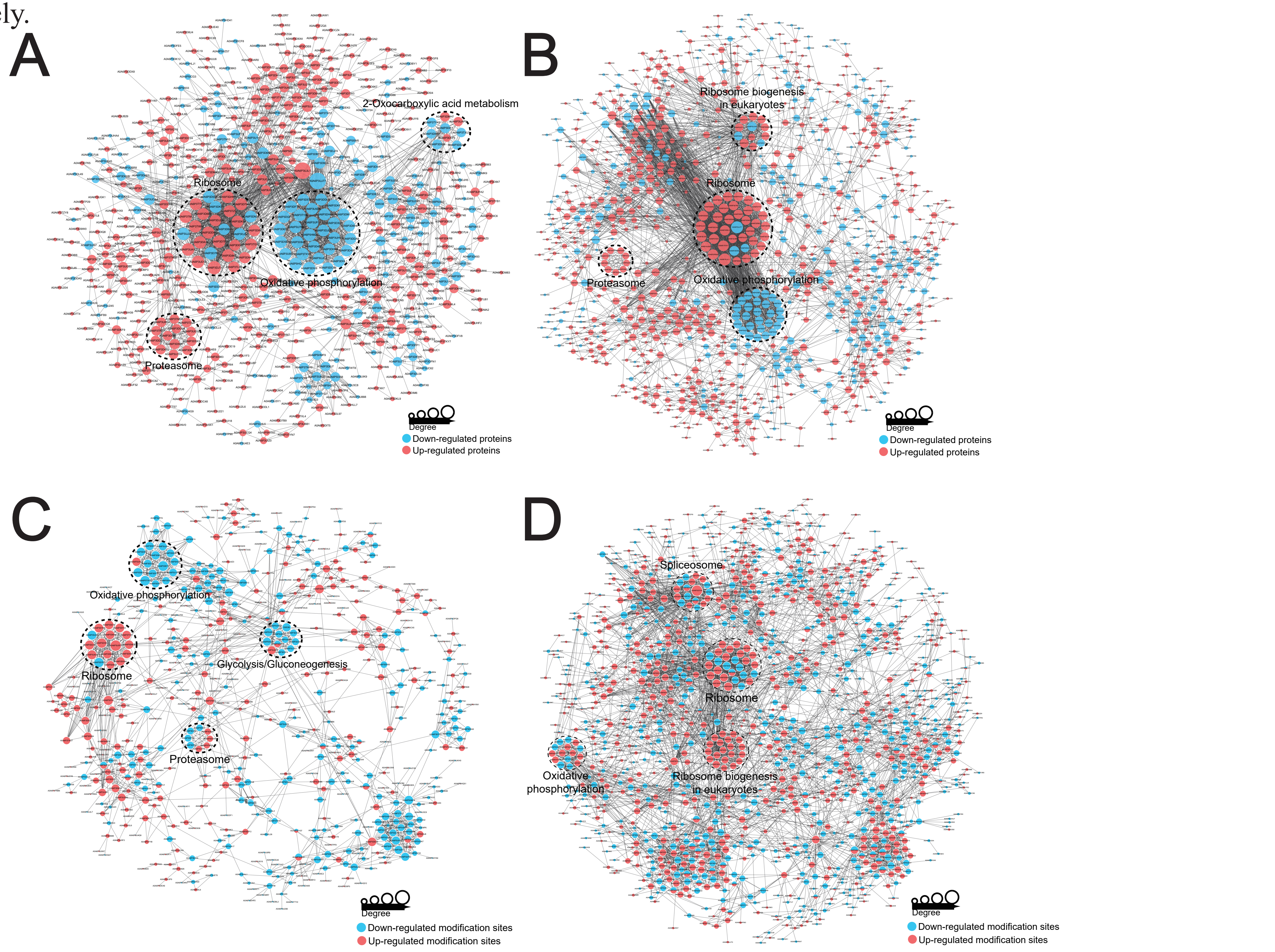

Supplement: Supplementary file 1 [file insects-13-00862-s001.zip › insects-1876268-supplementary/insects-1876268-proofed-supplementary/Figure S14.pdf]
