# Supplementary material for: Integrative Proteomic and Phosphoproteomic Analyses Revealed Complex Mechanisms Underlying Reproductive Diapause in Bombus terrestris Queens
Source: Insects. 2022 Sep 23;13(10):862. doi: 10.3390/insects13100862 (PMC9604461; doi:10.3390/insects13100862)

**Supplementary Figure S2:** Overview of proteomic analyses. (A) The distributions of raw MS/MS length and charge of peptides quantified from proteomic data. (B) The distribution of protein molecular weight. (C) Reproducibility analysis of the samples. (D) The amounts of DEPs in different comparable groups.

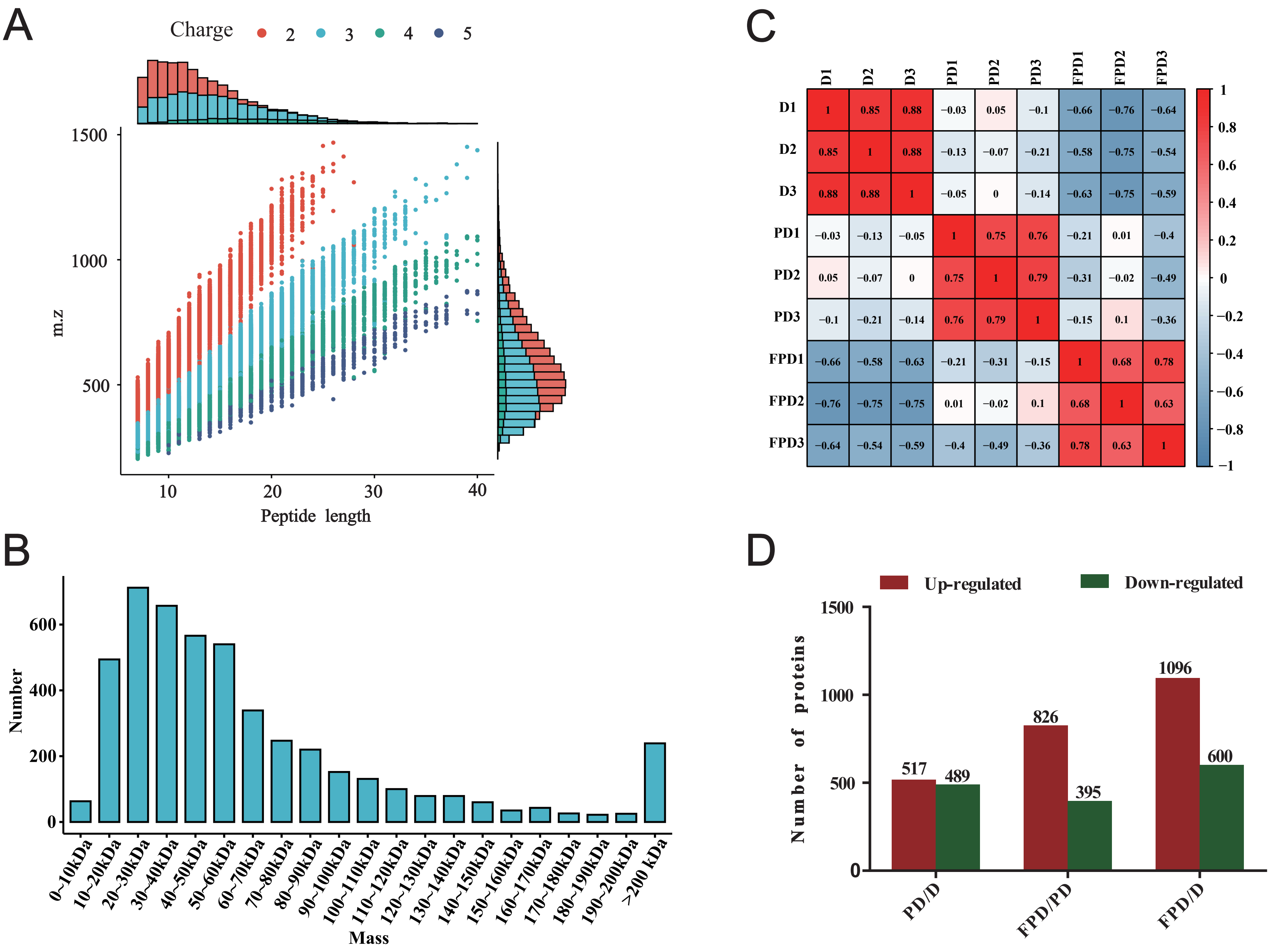

Supplement: Supplementary file 1 [file insects-13-00862-s001.zip › insects-1876268-supplementary/insects-1876268-proofed-supplementary/Figure S2.pdf]
