# Supplementary material for: Integrative Proteomic and Phosphoproteomic Analyses Revealed Complex Mechanisms Underlying Reproductive Diapause in Bombus terrestris Queens
Source: Insects. 2022 Sep 23;13(10):862. doi: 10.3390/insects13100862 (PMC9604461; doi:10.3390/insects13100862)
Supplement: Supplementary file 1 [file insects-13-00862-s001.zip › insects-1876268-supplementary/insects-1876268-proofed-supplementary/Figure S3.pdf]

Supplementary Figure S3: KEGG analysis of DEPs in each comparable group.

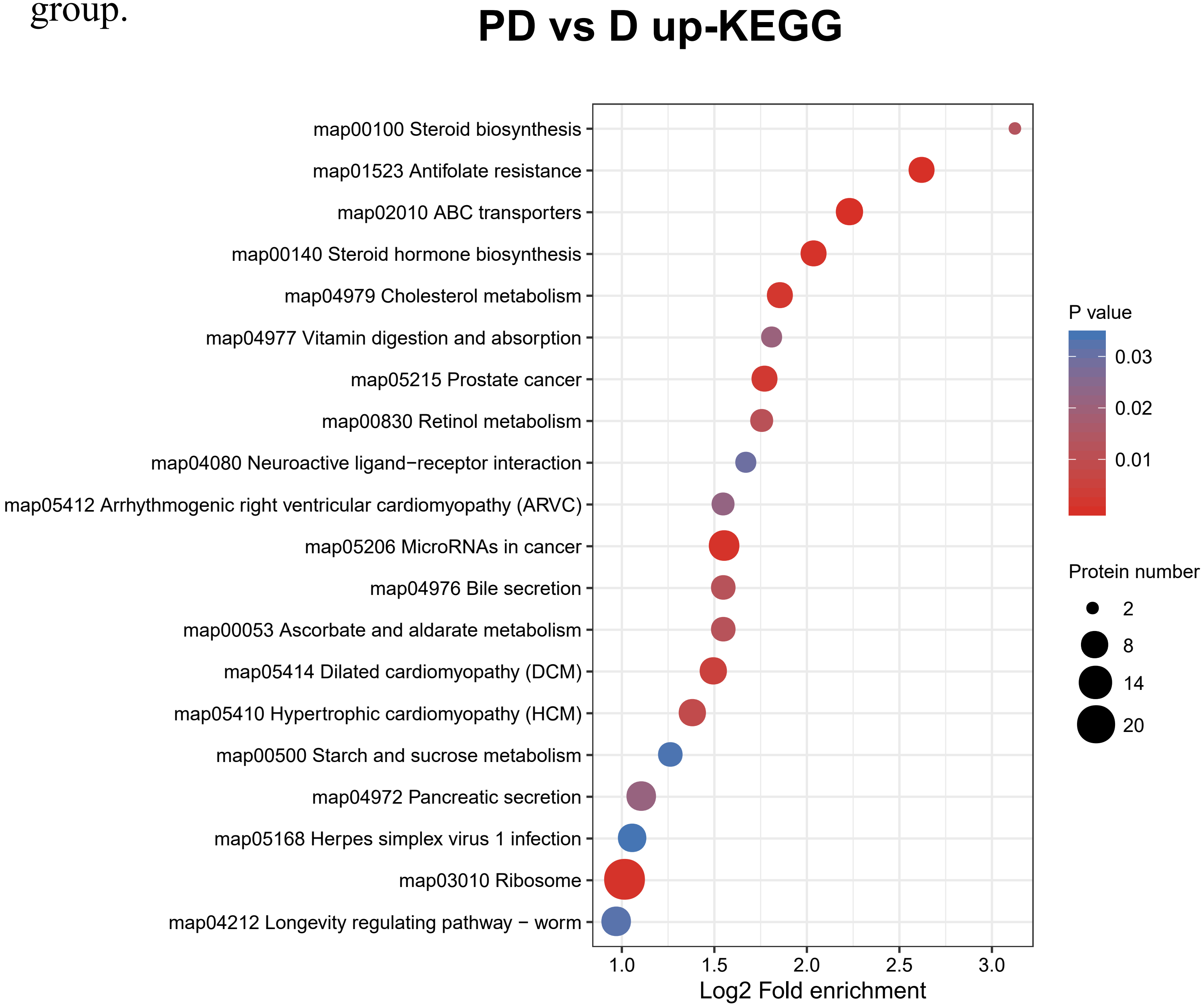

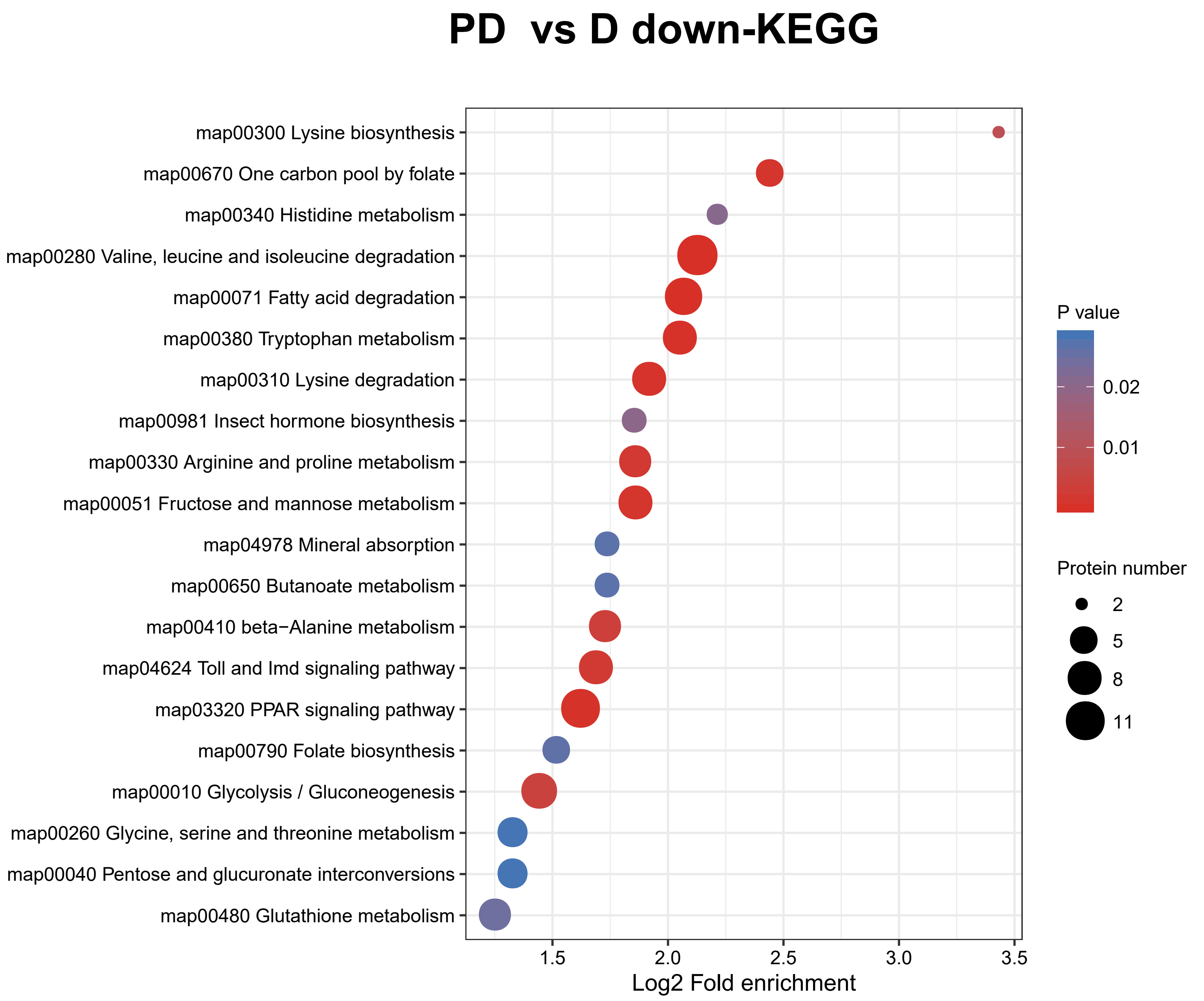
PD vs D down-KEGG
map00300 Lysine biosynthesis
map00670 One carbon pool by folate
map00340 Histidine metabolism
map00280 Valine, leucine and isoleucine degradation
map00071 Fatty acid degradation
map00380 Tryptophan metabolism
map00310 Lysine degradation
map00981 Insect hormone biosynthesis
map00330 Arginine and proline metabolism
map00051 Fructose and mannose metabolism
map04978 Mineral absorption
map00650 Butanoate metabolism
map00410 beta−Alanine metabolism
map04624 Toll and Imd signaling pathway
map03320 PPAR signaling pathway
map00790 Folate biosynthesis
map00010 Glycolysis / Gluconeogenesis
map00260 Glycine, serine and threonine metabolism
map00040 Pentose and glucuronate interconversions
map00480 Glutathione metabolism
1.5
2.0
2.5
3.0
3.5
Log2 Fold enrichment
P value
0.02
0.01
Protein number
2
5
8
11

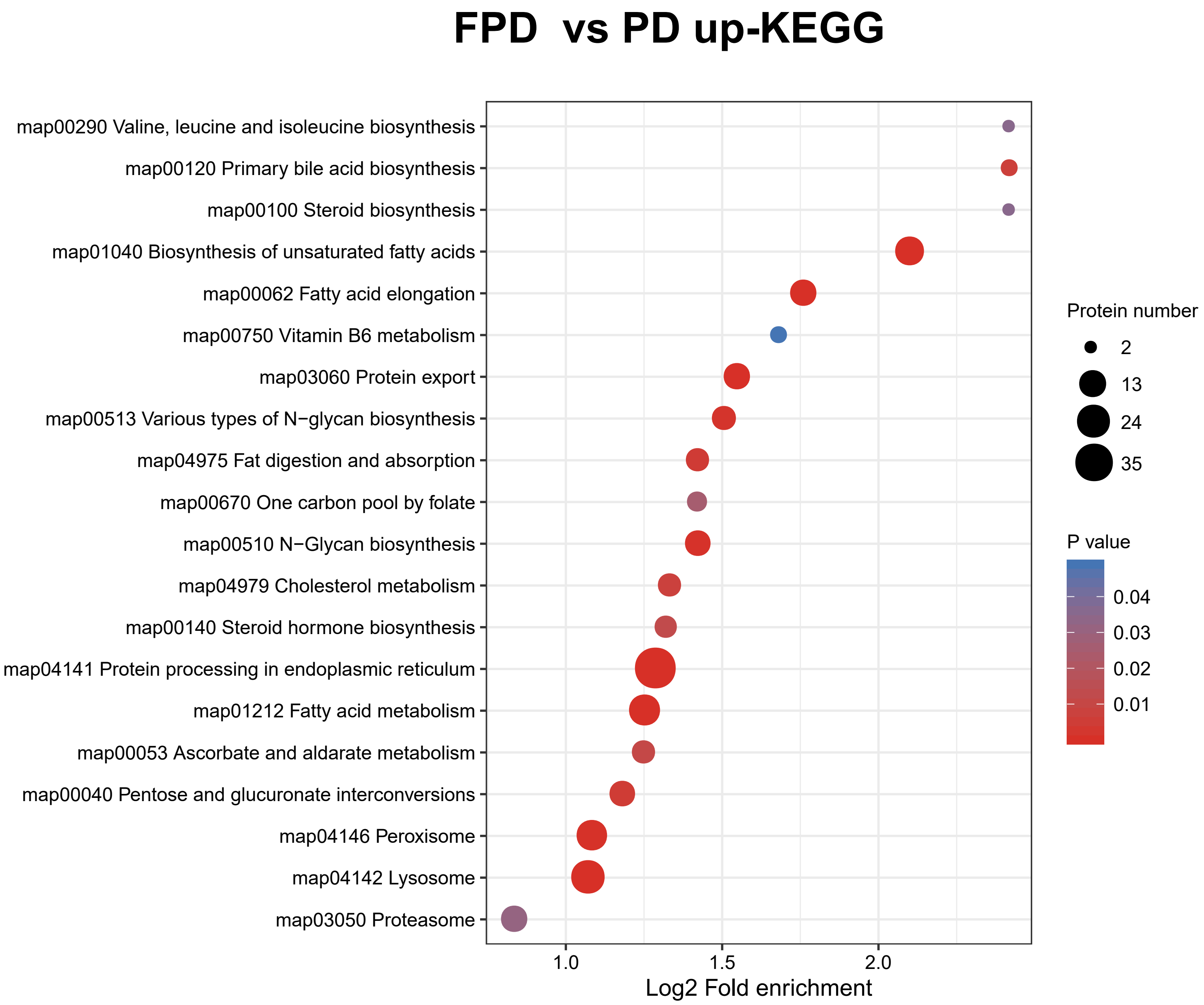
FPD vs PD up-KEGG
map00290 Valine, leucine and isoleucine biosynthesis
map00120 Primary bile acid biosynthesis
map00100 Steroid biosynthesis
map01040 Biosynthesis of unsaturated fatty acids
map00062 Fatty acid elongation
map00750 Vitamin B6 metabolism
map03060 Protein export
map00513 Various types of N−glycan biosynthesis
map04975 Fat digestion and absorption
map00670 One carbon pool by folate
map00510 N−Glycan biosynthesis
map04979 Cholesterol metabolism
map00140 Steroid hormone biosynthesis
map04141 Protein processing in endoplasmic reticulum
map01212 Fatty acid metabolism
map00053 Ascorbate and aldarate metabolism
map00040 Pentose and glucuronate interconversions
map04146 Peroxisome
map04142 Lysosome
map03050 Proteasome
1.0
1.5
2.0
Log2 Fold enrichment
Protein number
2
13
24
35
P value
0.04
0.03
0.02
0.01

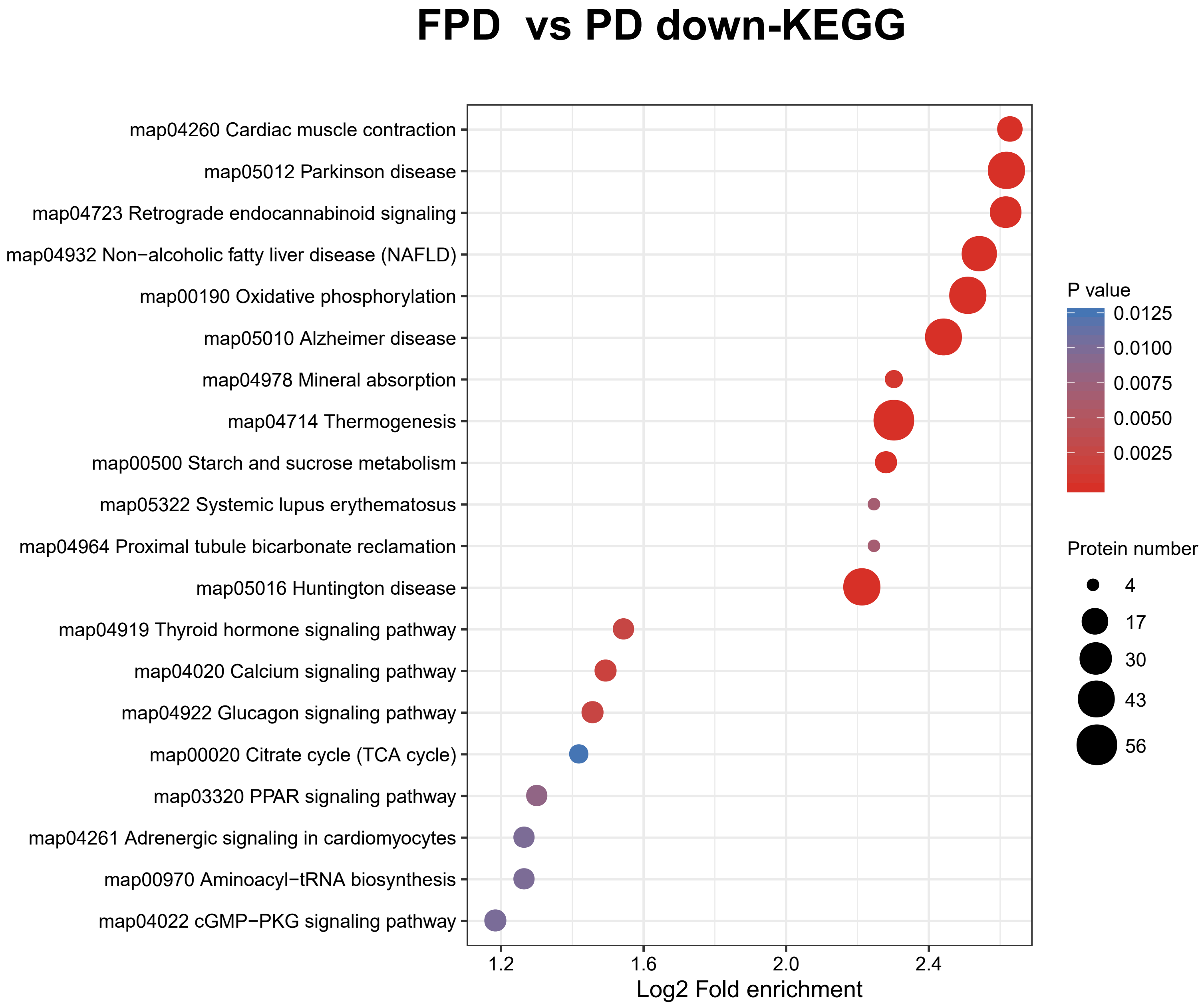

FPD vs PD down-KEGG
map04260 Cardiac muscle contraction
map05012 Parkinson disease
map04723 Retrograde endocannabinoid signaling
map04932 Non−alcoholic fatty liver disease (NAFLD)
map00190 Oxidative phosphorylation
map05010 Alzheimer disease
map04978 Mineral absorption
map04714 Thermogenesis
map00500 Starch and sucrose metabolism
map05322 Systemic lupus erythematosus
map04964 Proximal tubule bicarbonate reclamation
map05016 Huntington disease
map04919 Thyroid hormone signaling pathway
map04020 Calcium signaling pathway
map04922 Glucagon signaling pathway
map00020 Citrate cycle (TCA cycle)
map03320 PPAR signaling pathway
map04261 Adrenergic signaling in cardiomyocytes
map00970 Aminoacyl−tRNA biosynthesis
map04022 cGMP−PKG signaling pathway
1.2
1.6
2.0
2.4
Log2 Fold enrichment
P value
0.0125
0.0100
0.0075
0.0050
0.0025
Protein number
4
17
30
43
56

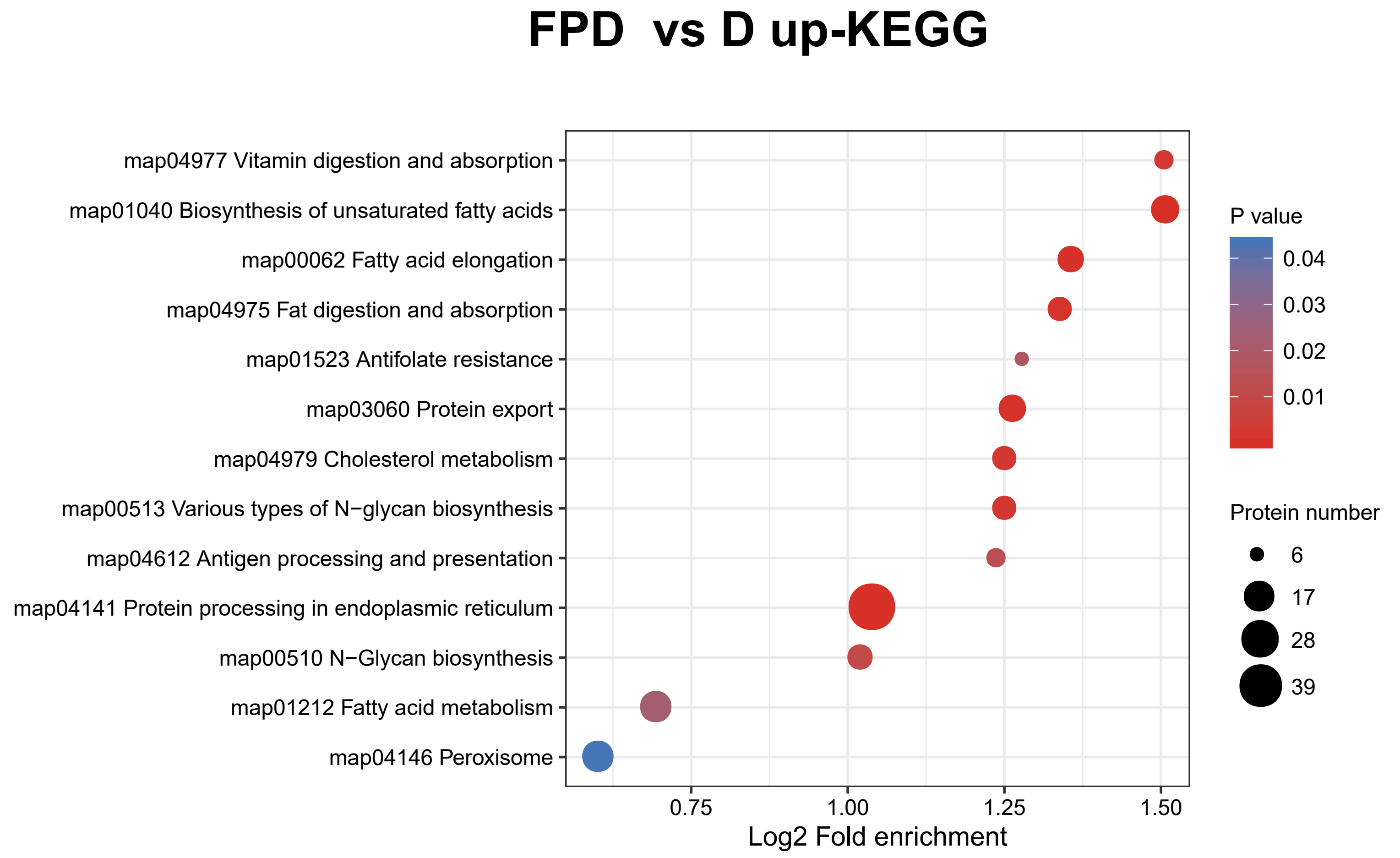
FPD vs D up-KEGG
map04977 Vitamin digestion and absorption
map01040 Biosynthesis of unsaturated fatty acids
map00062 Fatty acid elongation
map04975 Fat digestion and absorption
map01523 Antifolate resistance
map03060 Protein export
map04979 Cholesterol metabolism
map00513 Various types of N−glycan biosynthesis
map04612 Antigen processing and presentation
map04141 Protein processing in endoplasmic reticulum
map00510 N−Glycan biosynthesis
map01212 Fatty acid metabolism
map04146 Peroxisome
0.75
1.00
1.25
1.50
Log2 Fold enrichment
P value
0.04
0.03
0.02
0.01
Protein number
6
17
28
39

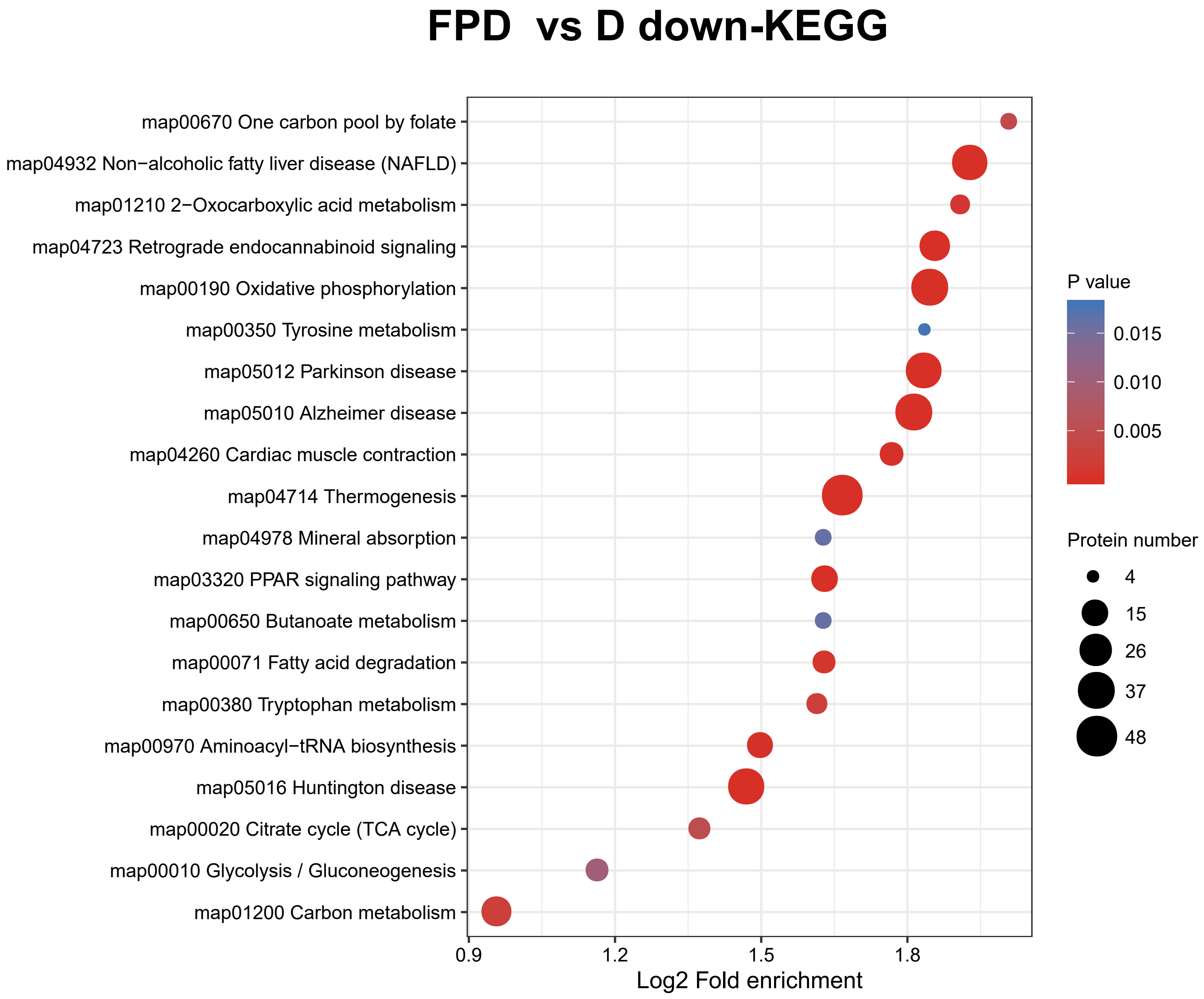

FPD vs D down-KEGG
map00670 One carbon pool by folate
map04932 Non−alcoholic fatty liver disease (NAFLD)
map01210 2−Oxocarboxylic acid metabolism
map04723 Retrograde endocannabinoid signaling
map00190 Oxidative phosphorylation
map00350 Tyrosine metabolism
map05012 Parkinson disease
map05010 Alzheimer disease
map04260 Cardiac muscle contraction
map04714 Thermogenesis
map04978 Mineral absorption
map03320 PPAR signaling pathway
map00650 Butanoate metabolism
map00071 Fatty acid degradation
map00380 Tryptophan metabolism
map00970 Aminoacyl−tRNA biosynthesis
map05016 Huntington disease
map00020 Citrate cycle (TCA cycle)
map00010 Glycolysis / Gluconeogenesis
map01200 Carbon metabolism
0.9
1.2
1.5
1.8
Log2 Fold enrichment
P value
0.015
0.010
0.005
Protein number
4
15
26
37
48
