# Supplementary material for: Integrative Proteomic and Phosphoproteomic Analyses Revealed Complex Mechanisms Underlying Reproductive Diapause in Bombus terrestris Queens
Source: Insects. 2022 Sep 23;13(10):862. doi: 10.3390/insects13100862 (PMC9604461; doi:10.3390/insects13100862)
Supplement: Supplementary file 1 [file insects-13-00862-s001.zip › insects-1876268-supplementary/insects-1876268-proofed-supplementary/Figure S4.pdf]

Supplementary Figure S4: Protein domain analysis of DEPs in each comparable group.

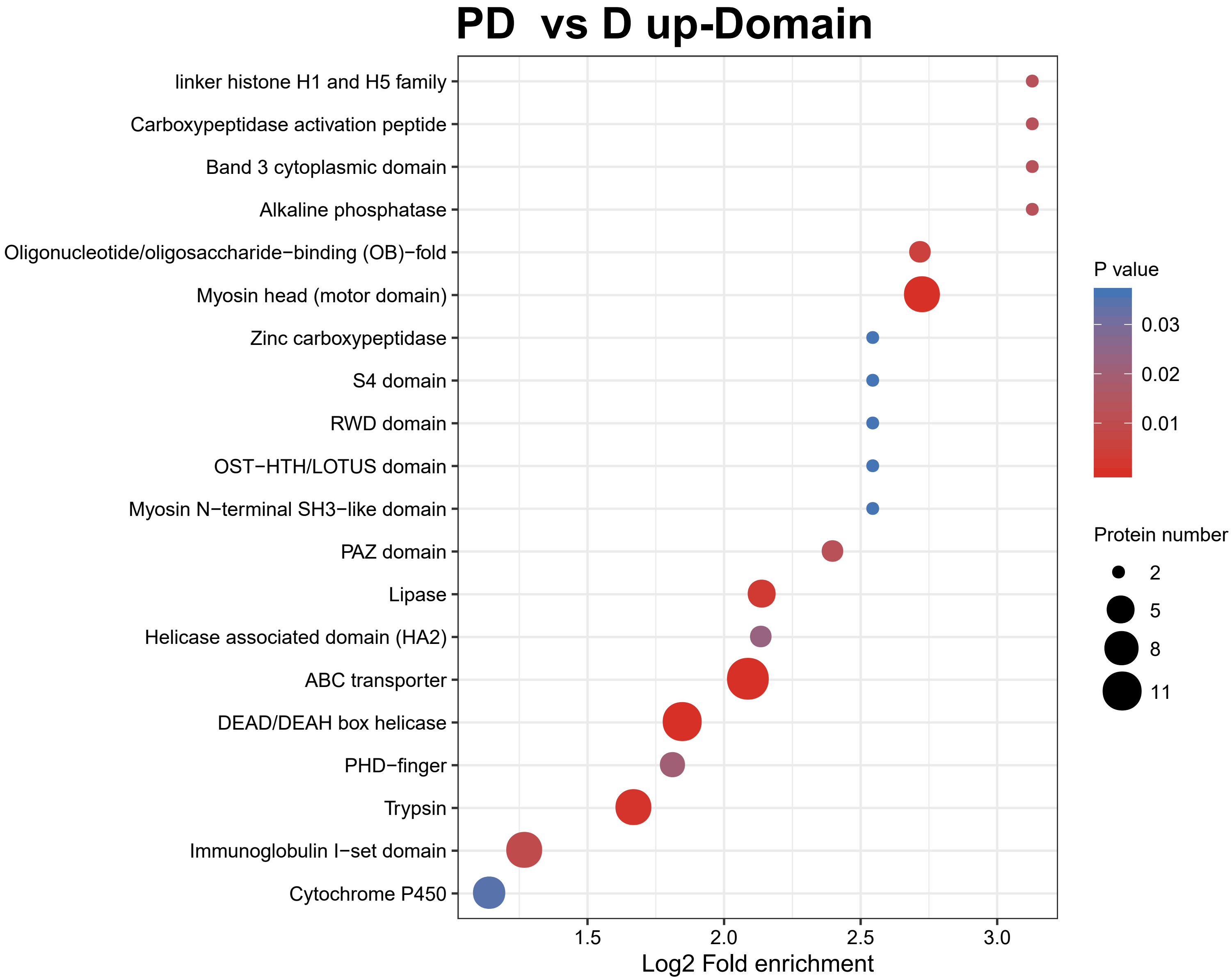

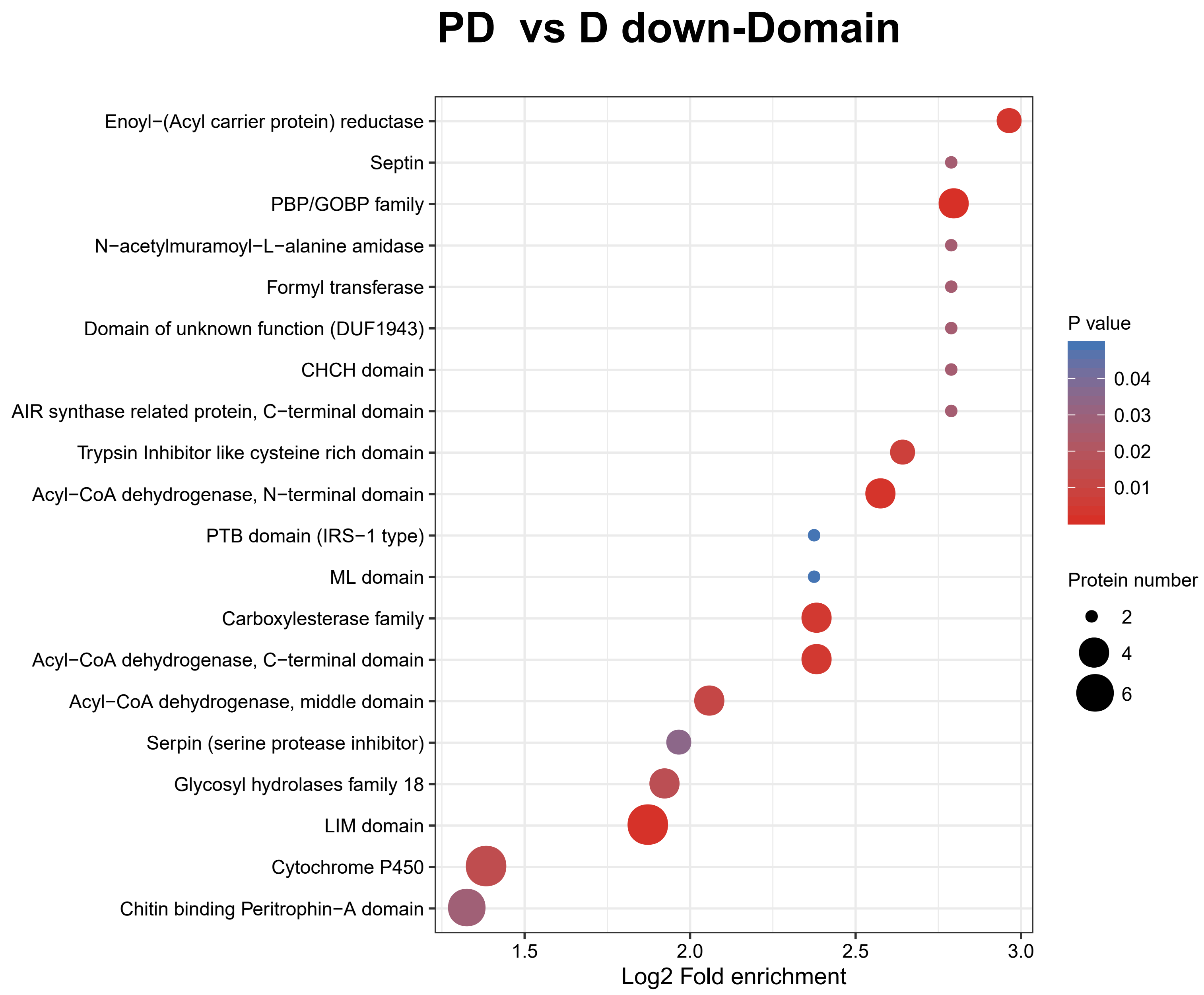
PD vs D down-Domain
Enoyl−(Acyl carrier protein) reductase
Septin
PBP/GOBP family
N−acetylmuramoyl−L−alanine amidase
Formyl transferase
Domain of unknown function (DUF1943)
CHCH domain
AIR synthase related protein, C−terminal domain
Trypsin Inhibitor like cysteine rich domain
Acyl−CoA dehydrogenase, N−terminal domain
PTB domain (IRS−1 type)
ML domain
Carboxylesterase family
Acyl−CoA dehydrogenase, C−terminal domain
Acyl−CoA dehydrogenase, middle domain
Serpin (serine protease inhibitor)
Glycosyl hydrolases family 18
LIM domain
Cytochrome P450
Chitin binding Peritrophin−A domain
1.5
2.0
2.5
3.0
Log2 Fold enrichment
P value
0.04
0.03
0.02
0.01
Protein number
2
4
6

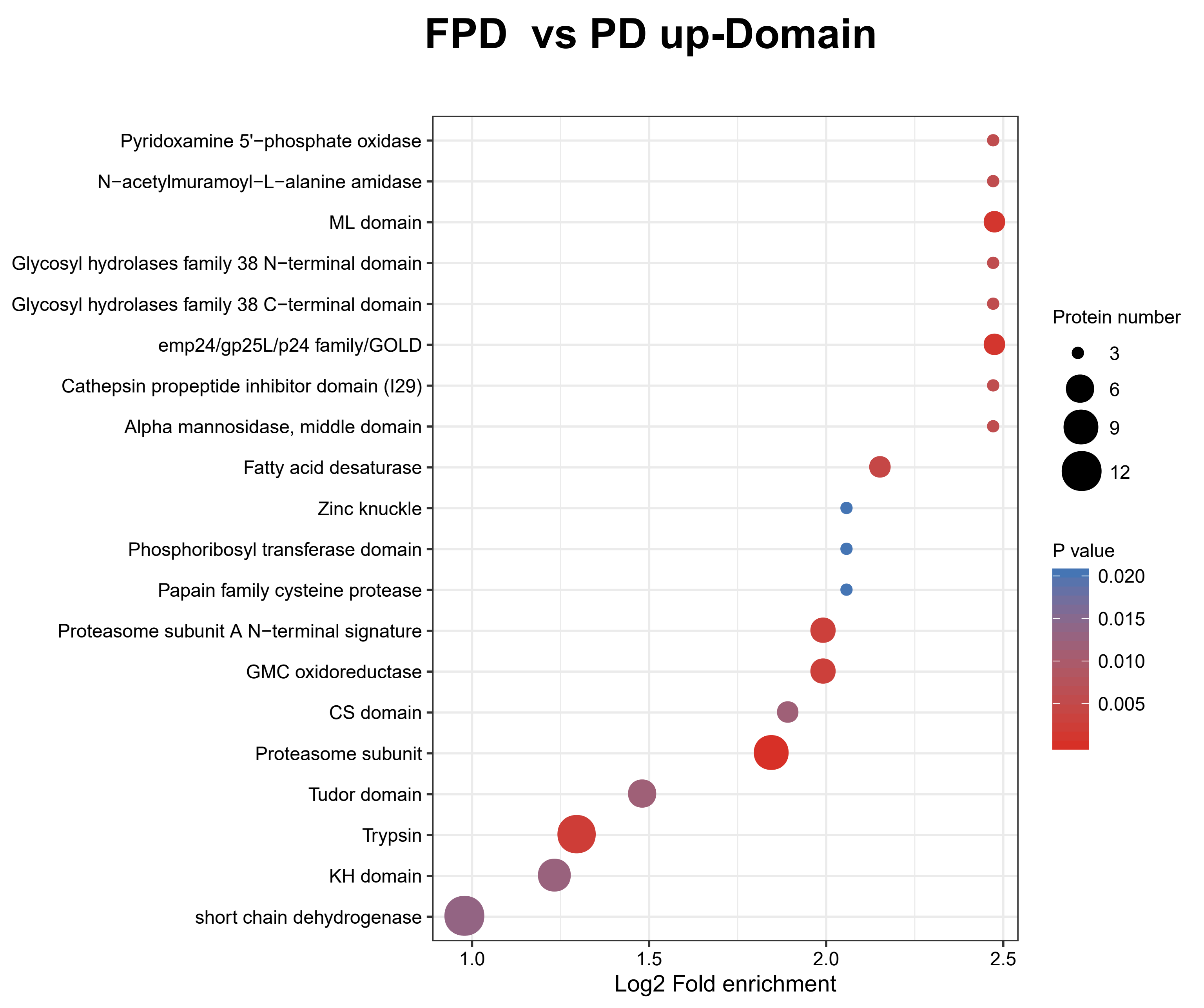
FPD vs PD up-Domain
Pyridoxamine 5'−phosphate oxidase
N−acetylmuramoyl−L−alanine amidase
ML domain
Glycosyl hydrolases family 38 N−terminal domain
Glycosyl hydrolases family 38 C−terminal domain
emp24/gp25L/p24 family/GOLD
Cathepsin propeptide inhibitor domain (I29)
Alpha mannosidase, middle domain
Fatty acid desaturase
Zinc knuckle
Phosphoribosyl transferase domain
Papain family cysteine protease
Proteasome subunit A N−terminal signature
GMC oxidoreductase
CS domain
Proteasome subunit
Tudor domain
Trypsin
KH domain
short chain dehydrogenase
1.0
1.5
2.0
2.5
Log2 Fold enrichment
Protein number
3
6
9
12
P value
0.020
0.015
0.010
0.005

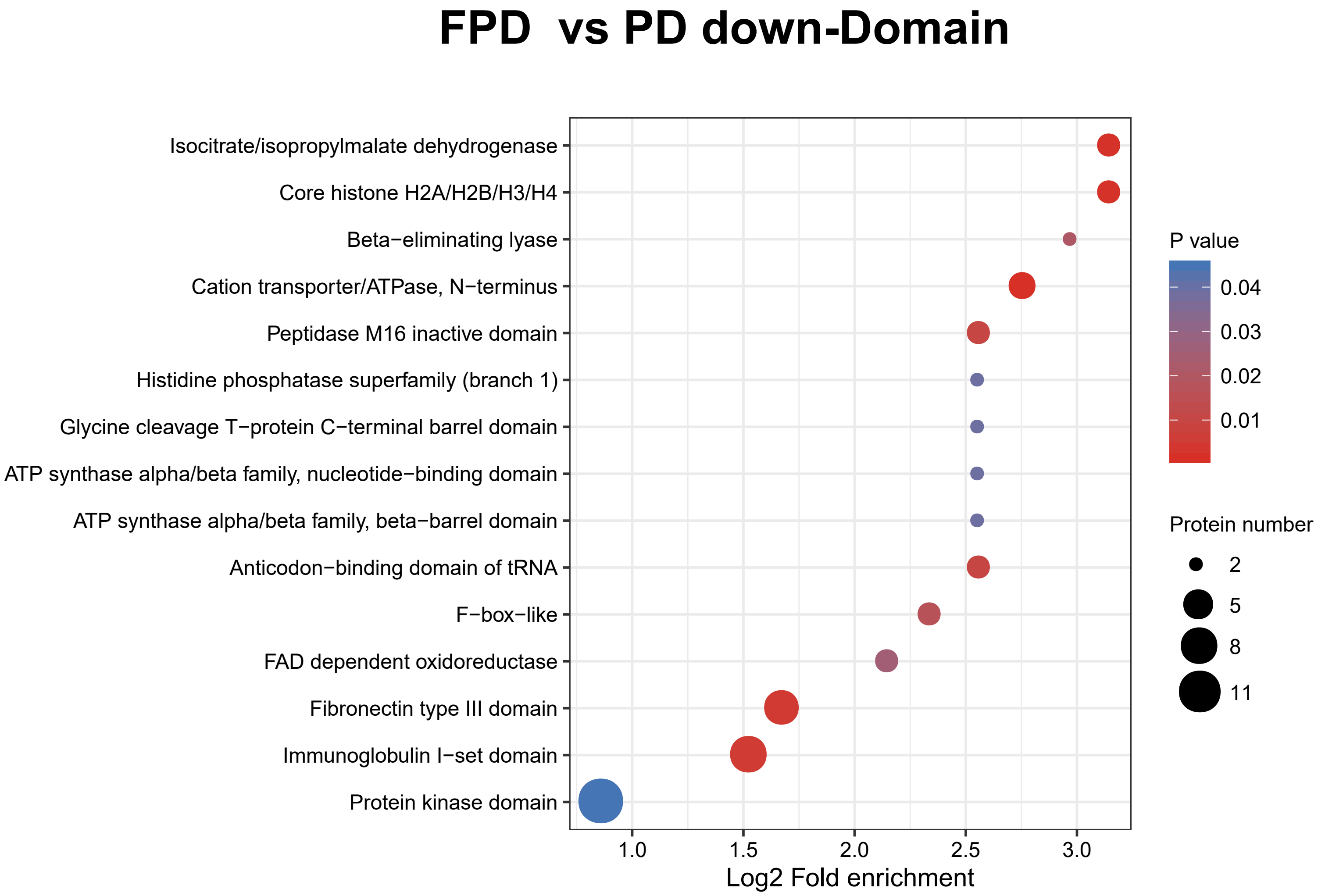
FPD vs PD down-Domain
Isocitrate/isopropylmalate dehydrogenase
Core histone H2A/H2B/H3/H4
Beta−eliminating lyase
Cation transporter/ATPase, N−terminus
Peptidase M16 inactive domain
Histidine phosphatase superfamily (branch 1)
Glycine cleavage T−protein C−terminal barrel domain
ATP synthase alpha/beta family, nucleotide−binding domain
ATP synthase alpha/beta family, beta−barrel domain
Anticodon−binding domain of tRNA
F−box−like
FAD dependent oxidoreductase
Fibronectin type III domain
Immunoglobulin I−set domain
Protein kinase domain
1.0
1.5
2.0
2.5
3.0
Log2 Fold enrichment
P value
0.04
0.03
0.02
0.01
Protein number
2
5
8
11

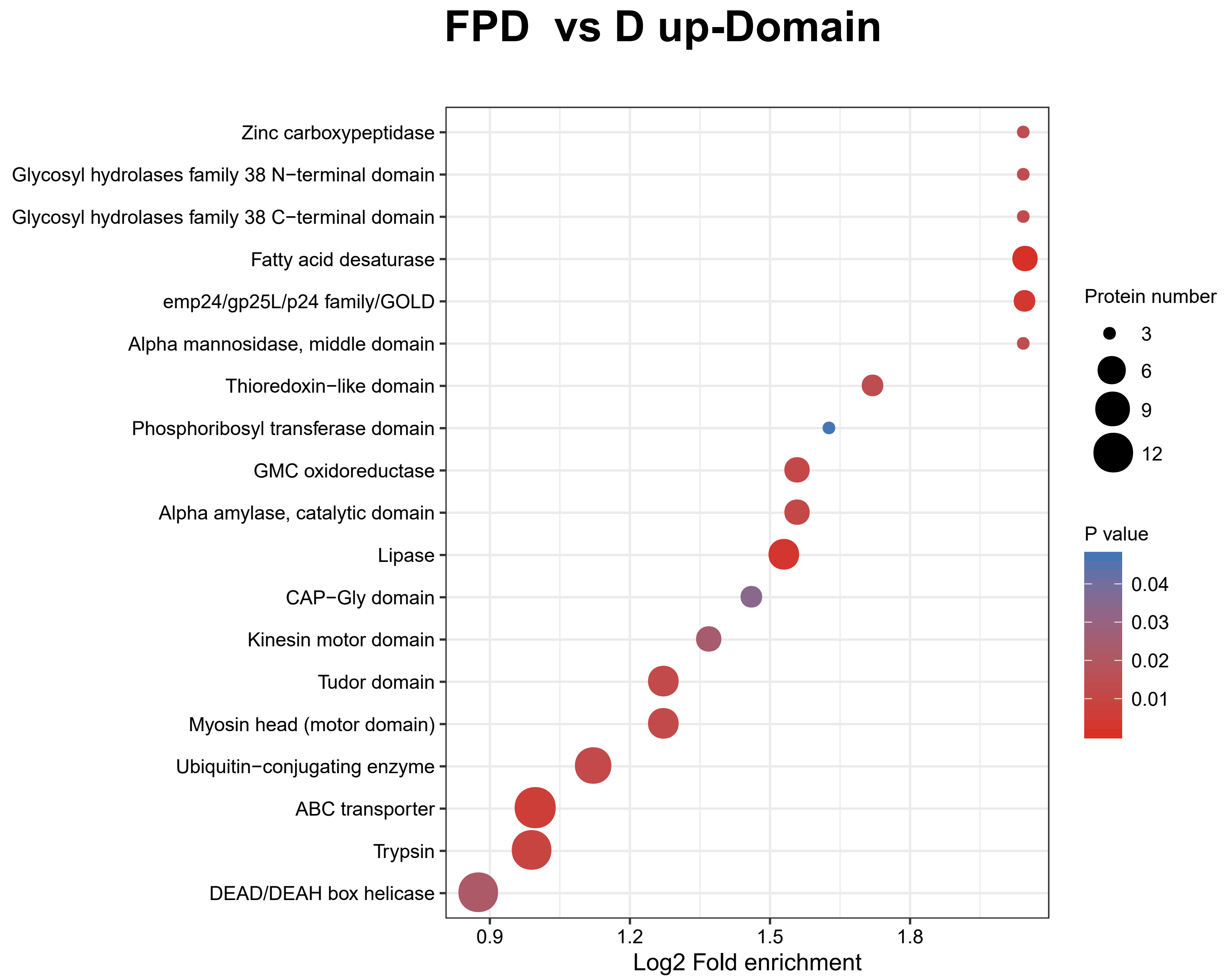
FPD vs D up-Domain
Zinc carboxypeptidase
Glycosyl hydrolases family 38 N−terminal domain
Glycosyl hydrolases family 38 C−terminal domain
Fatty acid desaturase
emp24/gp25L/p24 family/GOLD
Alpha mannosidase, middle domain
Thioredoxin−like domain
Phosphoribosyl transferase domain
GMC oxidoreductase
Alpha amylase, catalytic domain
Lipase
CAP−Gly domain
Kinesin motor domain
Tudor domain
Myosin head (motor domain)
Ubiquitin−conjugating enzyme
ABC transporter
Trypsin
DEAD/DEAH box helicase
0.9
1.2
1.5
1.8
Log2 Fold enrichment
Protein number
3
6
9
12
P value
0.04
0.03
0.02
0.01

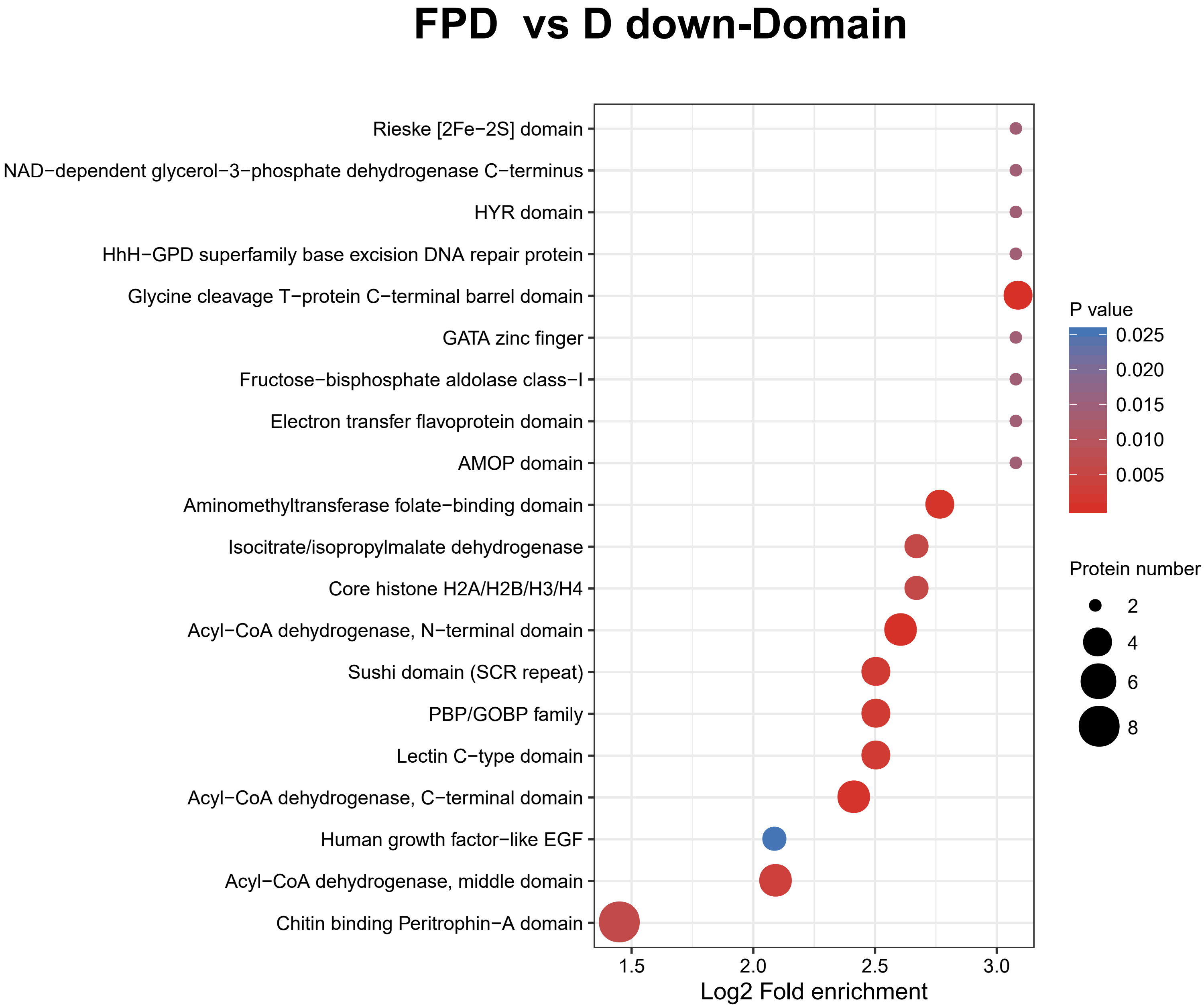
FPD vs D down-Domain
Rieske [2Fe−2S] domain
NAD−dependent glycerol−3−phosphate dehydrogenase C−terminus
HYR domain
HhH−GPD superfamily base excision DNA repair protein
Glycine cleavage T−protein C−terminal barrel domain
GATA zinc finger
Fructose−bisphosphate aldolase class−I
Electron transfer flavoprotein domain
AMOP domain
Aminomethyltransferase folate−binding domain
Isocitrate/isopropylmalate dehydrogenase
Core histone H2A/H2B/H3/H4
Acyl−CoA dehydrogenase, N−terminal domain
Sushi domain (SCR repeat)
PBP/GOBP family
Lectin C−type domain
Acyl−CoA dehydrogenase, C−terminal domain
Human growth factor−like EGF
Acyl−CoA dehydrogenase, middle domain
Chitin binding Peritrophin−A domain
1.5
2.0
2.5
3.0
Log2 Fold enrichment
P value
0.025
0.020
0.015
0.010
0.005
Protein number
2
4
6
8
