# Supplementary material for: Integrative Proteomic and Phosphoproteomic Analyses Revealed Complex Mechanisms Underlying Reproductive Diapause in Bombus terrestris Queens
Source: Insects. 2022 Sep 23;13(10):862. doi: 10.3390/insects13100862 (PMC9604461; doi:10.3390/insects13100862)
Supplement: Supplementary file 1 [file insects-13-00862-s001.zip › insects-1876268-supplementary/insects-1876268-proofed-supplementary/Figure S5.pdf]

Supplementary Figure S5: GO analysis of DEPs in each comparable group.

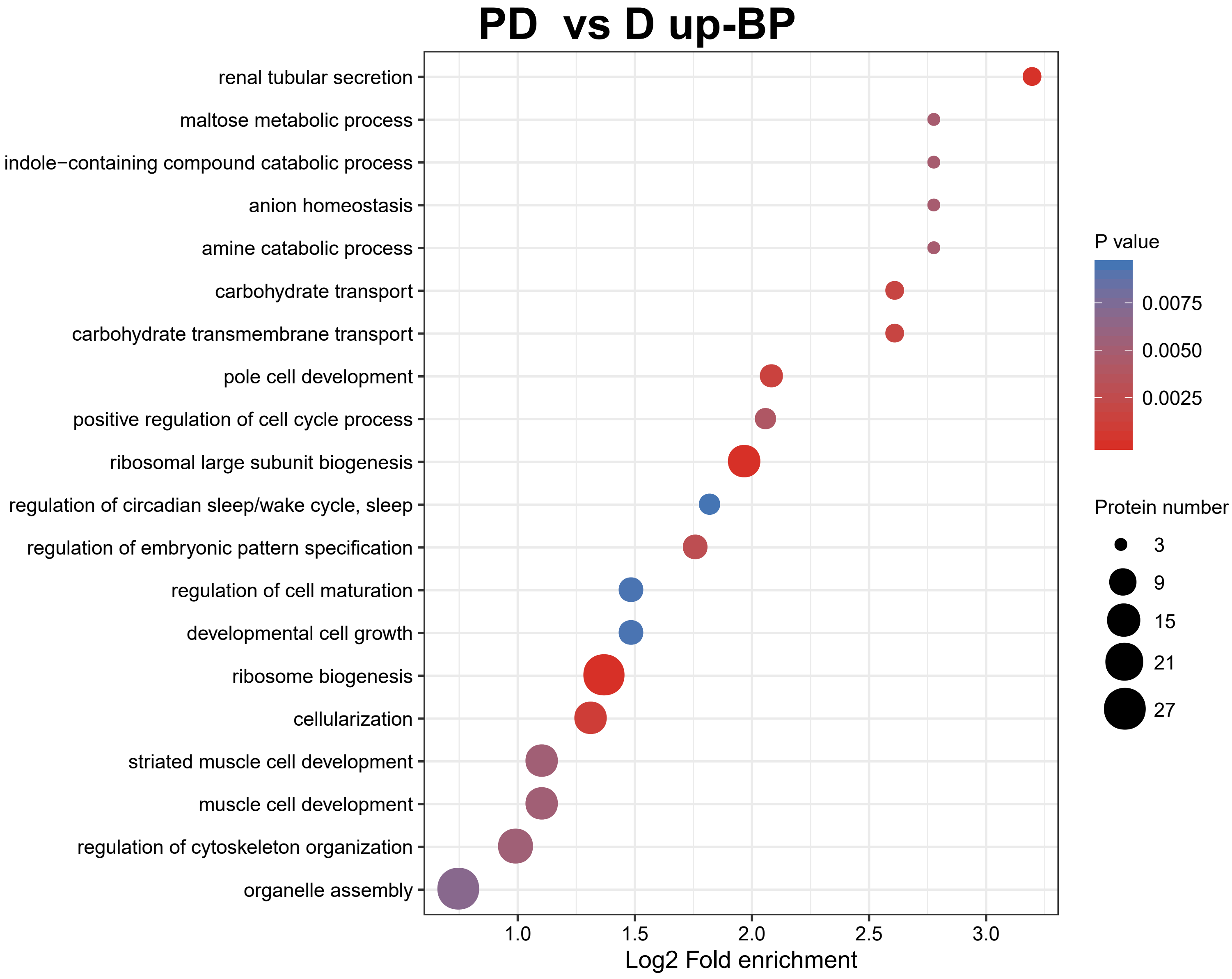

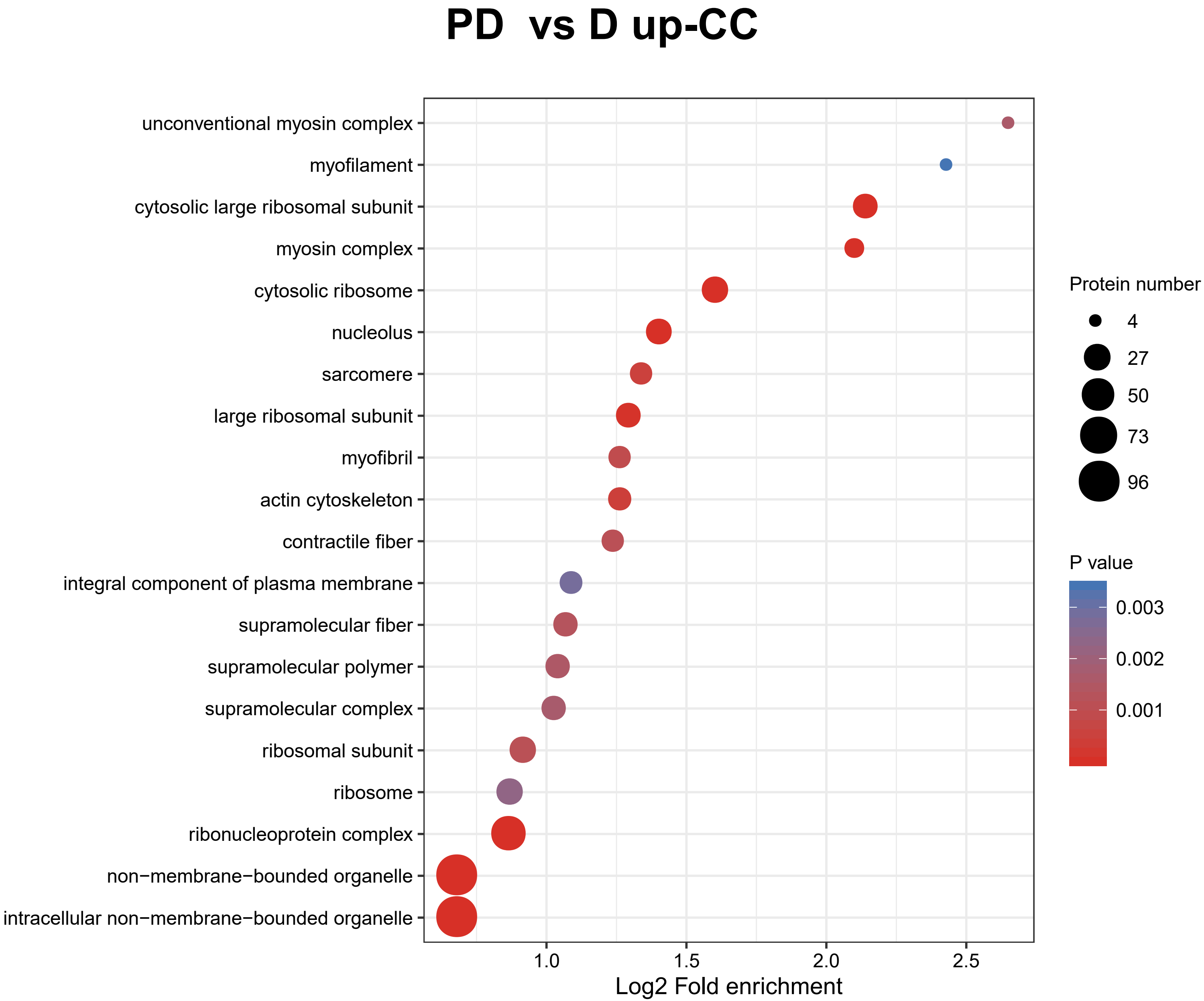
PD vs D up-CC
unconventional myosin complex
myofilament
cytosolic large ribosomal subunit
myosin complex
cytosolic ribosome
nucleolus
sarcomere
large ribosomal subunit
myofibril
actin cytoskeleton
contractile fiber
integral component of plasma membrane
supramolecular fiber
supramolecular polymer
supramolecular complex
ribosomal subunit
ribosome
ribonucleoprotein complex
non−membrane−bounded organelle
intracellular non−membrane−bounded organelle
1.0
1.5
2.0
2.5
Log2 Fold enrichment
Protein number
4
27
50
73
96
P value
0.003
0.002
0.001

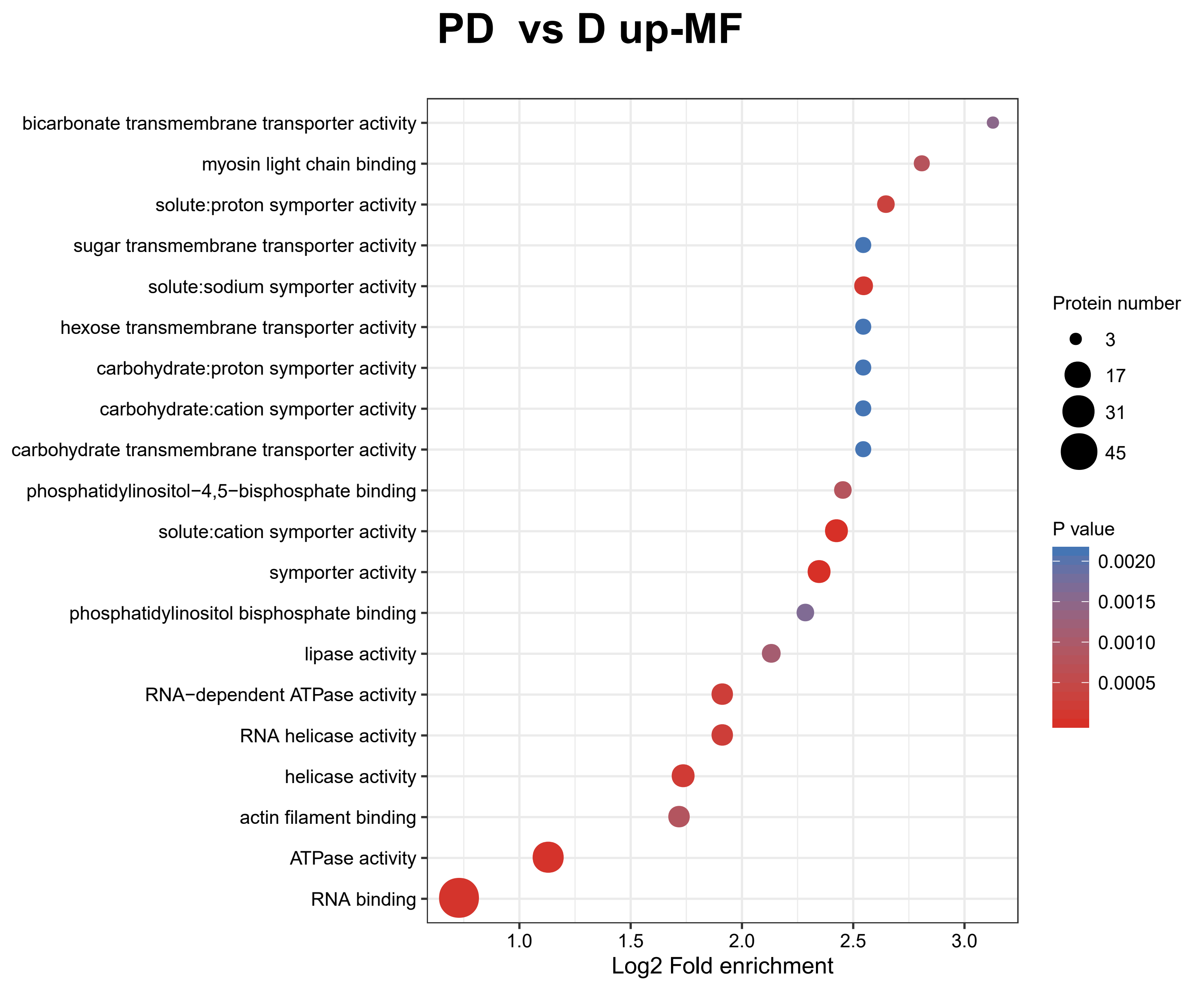
PD vs D up-MF
bicarbonate transmembrane transporter activity
myosin light chain binding
solute:proton symporter activity
sugar transmembrane transporter activity
solute:sodium symporter activity
hexose transmembrane transporter activity
carbohydrate:proton symporter activity
carbohydrate:cation symporter activity
carbohydrate transmembrane transporter activity
phosphatidylinositol−4,5−bisphosphate binding
solute:cation symporter activity
symporter activity
phosphatidylinositol bisphosphate binding
lipase activity
RNA−dependent ATPase activity
RNA helicase activity
helicase activity
actin filament binding
ATPase activity
RNA binding
1.0
1.5
2.0
2.5
3.0
Log2 Fold enrichment
Protein number
3
17
31
45
P value
0.0020
0.0015
0.0010
0.0005

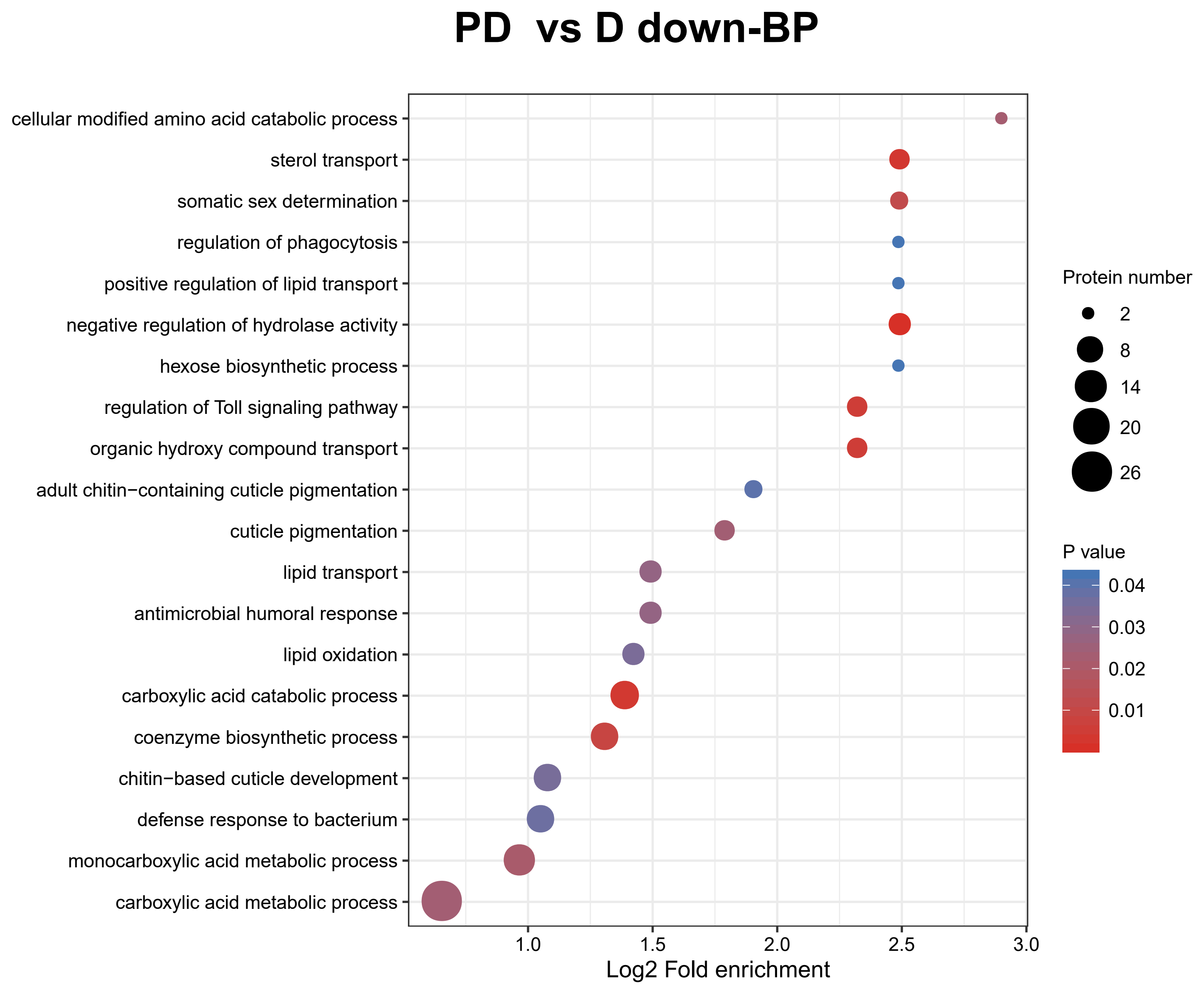
PD vs D down-BP
cellular modified amino acid catabolic process
sterol transport
somatic sex determination
regulation of phagocytosis
positive regulation of lipid transport
negative regulation of hydrolase activity
hexose biosynthetic process
regulation of Toll signaling pathway
organic hydroxy compound transport
adult chitin−containing cuticle pigmentation
cuticle pigmentation
lipid transport
antimicrobial humoral response
lipid oxidation
carboxylic acid catabolic process
coenzyme biosynthetic process
chitin−based cuticle development
defense response to bacterium
monocarboxylic acid metabolic process
carboxylic acid metabolic process
1.0
1.5
2.0
2.5
3.0
Log2 Fold enrichment
Protein number
2
8
14
20
26
P value
0.04
0.03
0.02
0.01

PD vs D down-CC

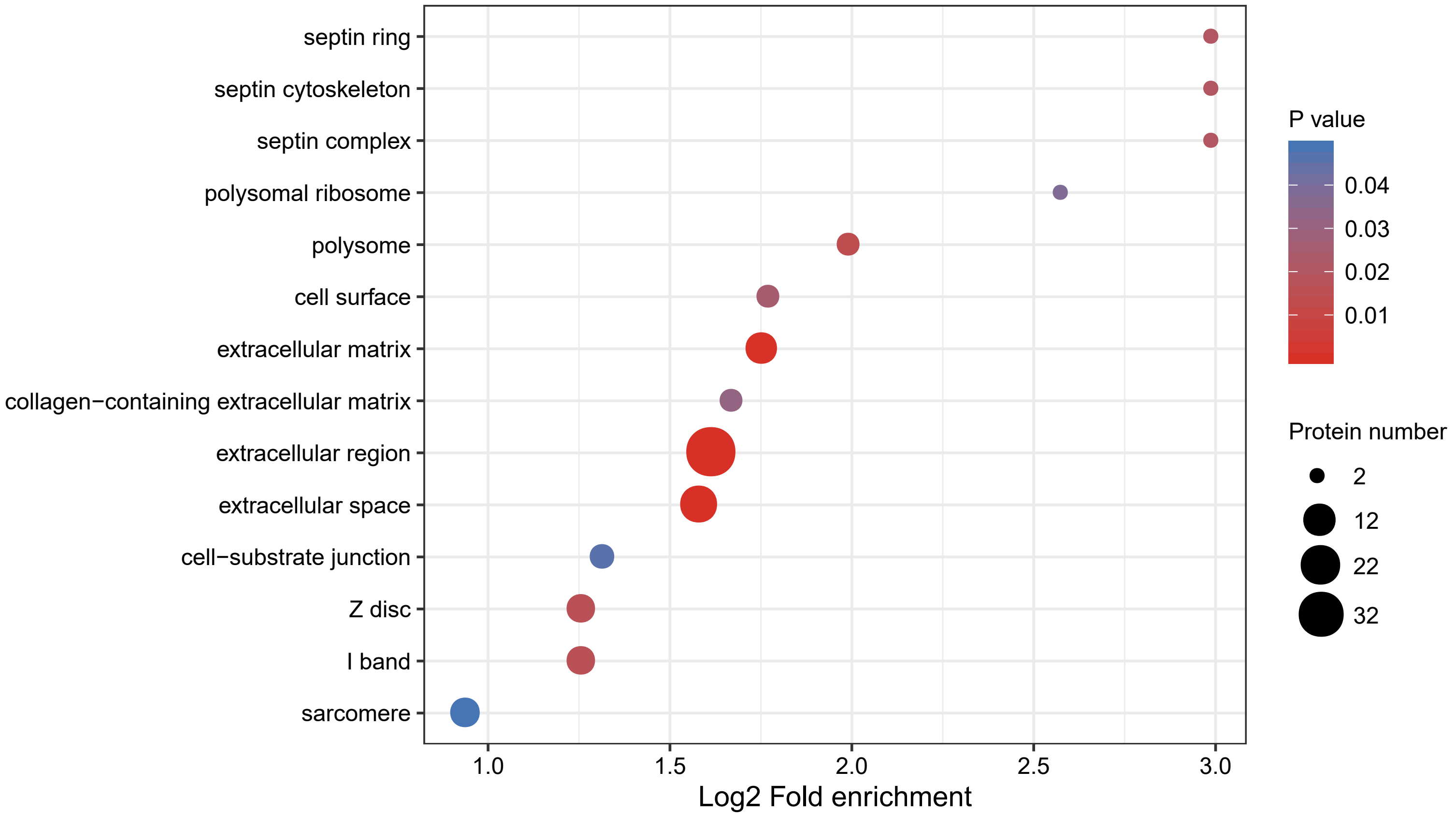

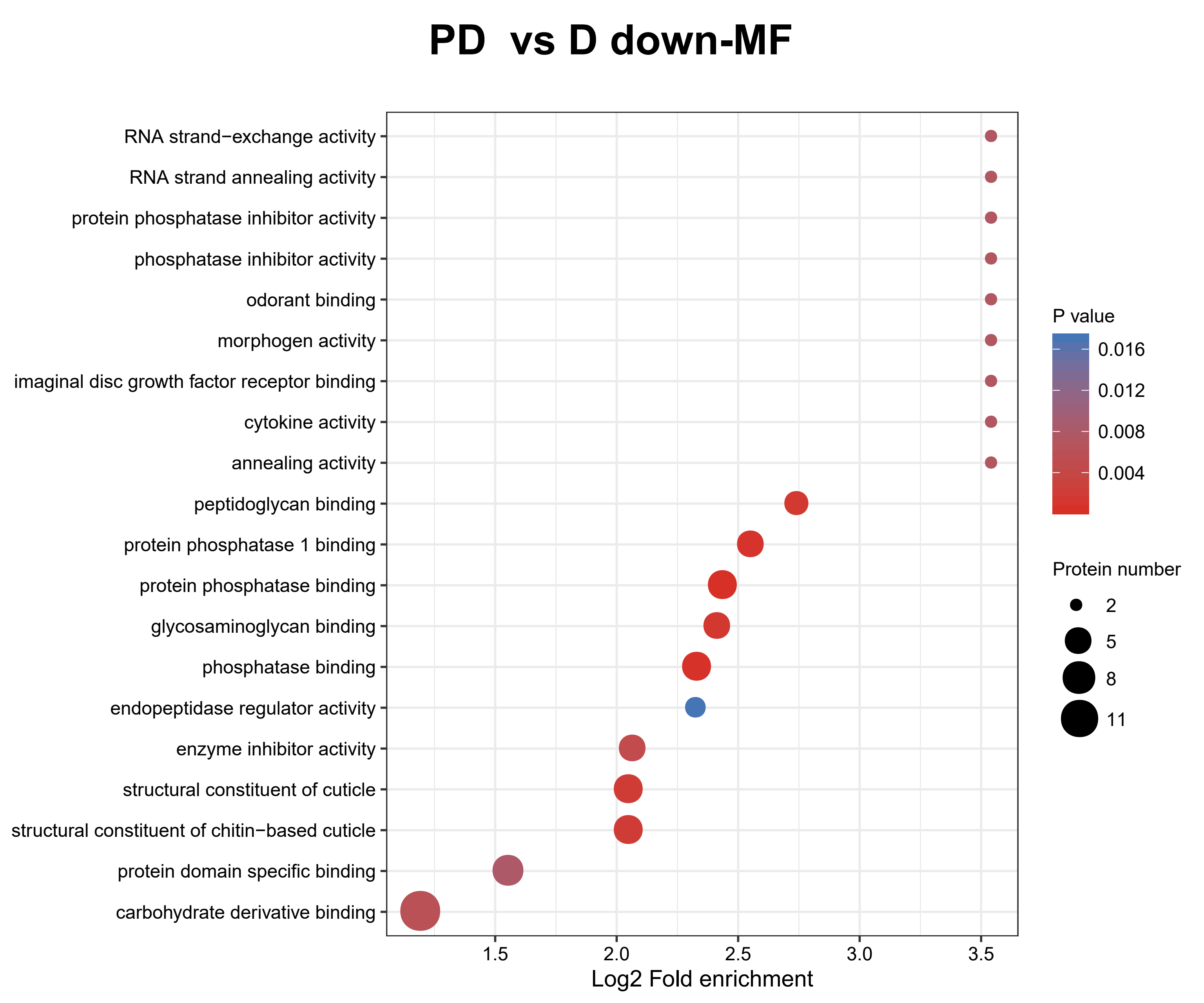

PD vs D down-MF
RNA strand−exchange activity
RNA strand annealing activity
protein phosphatase inhibitor activity
phosphatase inhibitor activity
odorant binding
morphogen activity
imaginal disc growth factor receptor binding
cytokine activity
annealing activity
peptidoglycan binding
protein phosphatase 1 binding
protein phosphatase binding
glycosaminoglycan binding
phosphatase binding
endopeptidase regulator activity
enzyme inhibitor activity
structural constituent of cuticle
structural constituent of chitin−based cuticle
protein domain specific binding
carbohydrate derivative binding
1.5
2.0
2.5
3.0
3.5
Log2 Fold enrichment
P value
0.016
0.012
0.008
0.004
Protein number
2
5
8
11

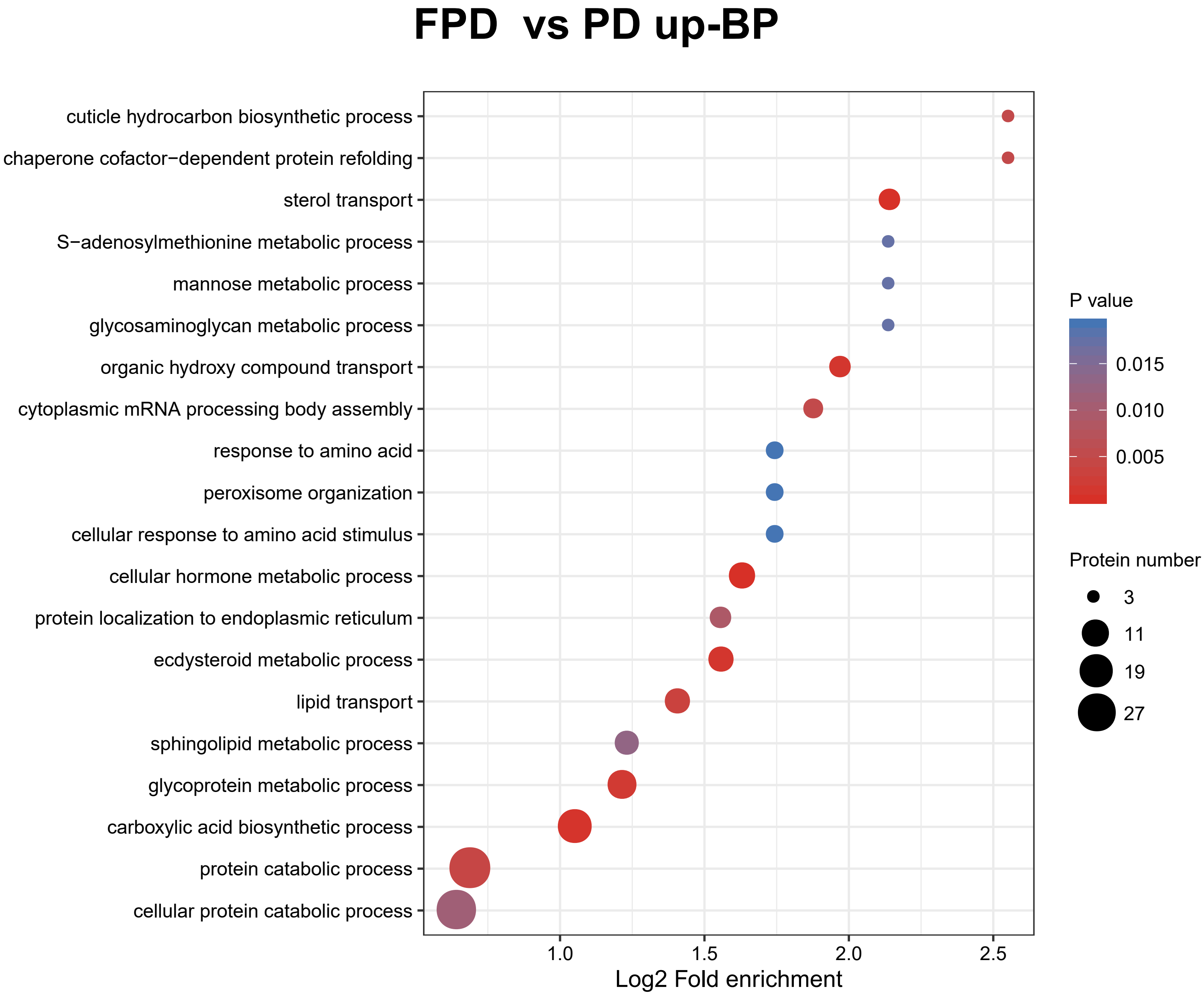
FPD vs PD up-BP
cuticle hydrocarbon biosynthetic process
chaperone cofactor−dependent protein refolding
sterol transport
S−adenosylmethionine metabolic process
mannose metabolic process
glycosaminoglycan metabolic process
organic hydroxy compound transport
cytoplasmic mRNA processing body assembly
response to amino acid
peroxisome organization
cellular response to amino acid stimulus
cellular hormone metabolic process
protein localization to endoplasmic reticulum
ecdysteroid metabolic process
lipid transport
sphingolipid metabolic process
glycoprotein metabolic process
carboxylic acid biosynthetic process
protein catabolic process
cellular protein catabolic process
1.0
1.5
2.0
2.5
Log2 Fold enrichment
P value
0.015
0.010
0.005
Protein number
3
11
19
27

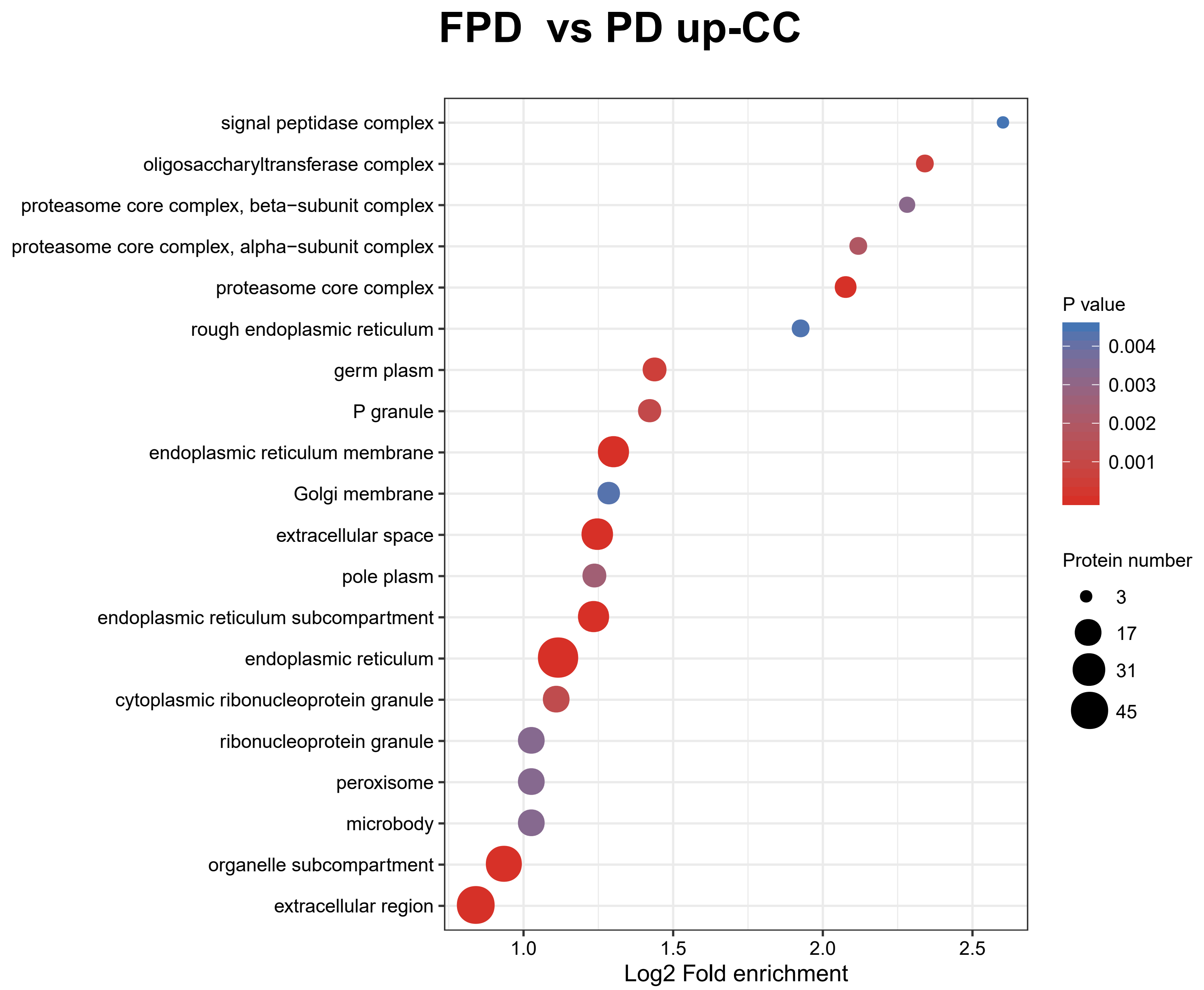

FPD vs PD up-CC
signal peptidase complex
oligosaccharyltransferase complex
proteasome core complex, beta−subunit complex
proteasome core complex, alpha−subunit complex
proteasome core complex
rough endoplasmic reticulum
germ plasm
P granule
endoplasmic reticulum membrane
Golgi membrane
extracellular space
pole plasm
endoplasmic reticulum subcompartment
endoplasmic reticulum
cytoplasmic ribonucleoprotein granule
ribonucleoprotein granule
peroxisome
microbody
organelle subcompartment
extracellular region
1.0
1.5
2.0
2.5
Log2 Fold enrichment
P value
0.004
0.003
0.002
0.001
Protein number
3
17
31
45

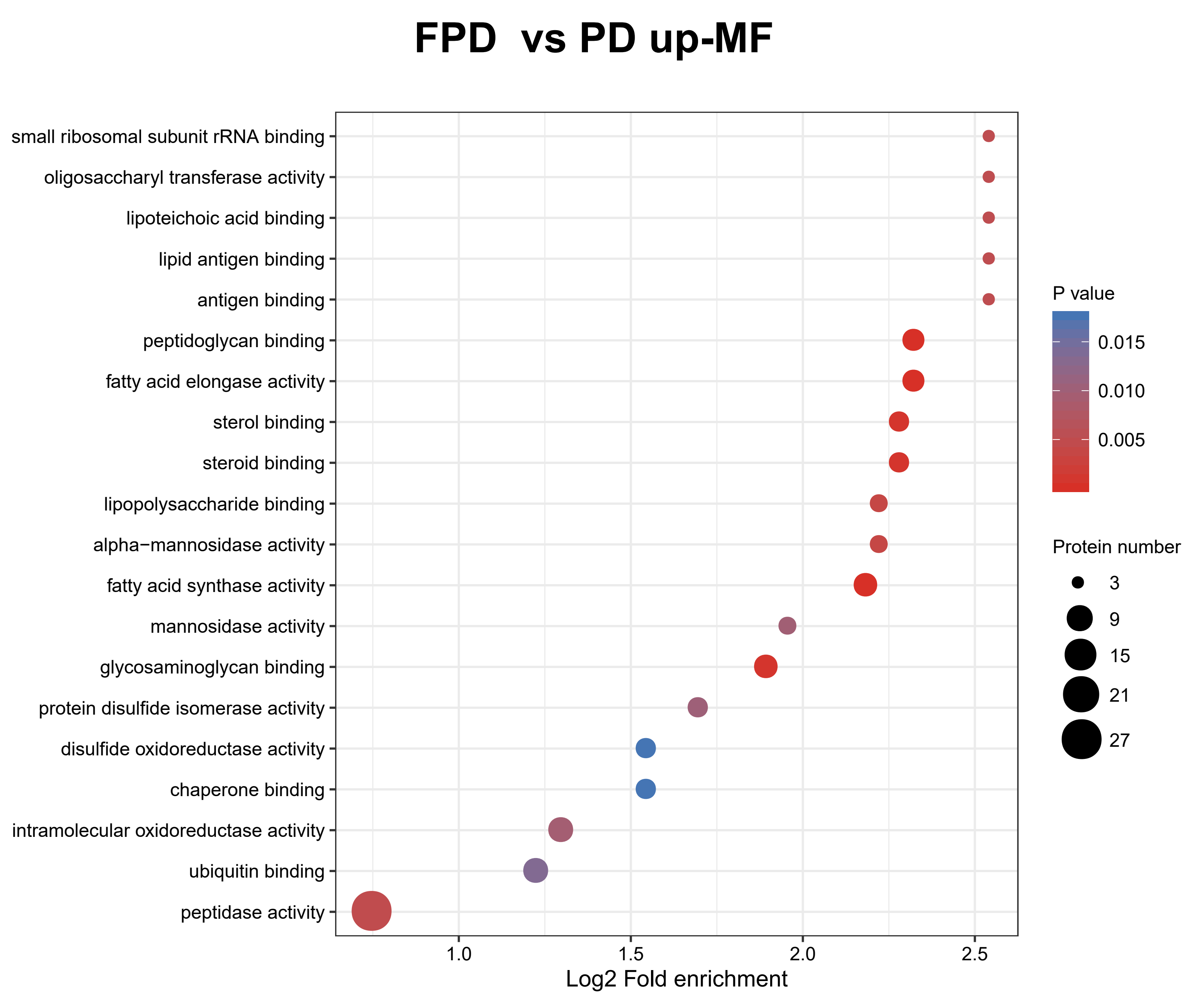

FPD vs PD up-MF
small ribosomal subunit rRNA binding
oligosaccharyl transferase activity
lipoteichoic acid binding
lipid antigen binding
antigen binding
peptidoglycan binding
fatty acid elongase activity
sterol binding
steroid binding
lipopolysaccharide binding
alpha−mannosidase activity
fatty acid synthase activity
mannosidase activity
glycosaminoglycan binding
protein disulfide isomerase activity
disulfide oxidoreductase activity
chaperone binding
intramolecular oxidoreductase activity
ubiquitin binding
peptidase activity
1.0
1.5
2.0
2.5
Log2 Fold enrichment
P value
0.015
0.010
0.005
Protein number
3
9
15
21
27

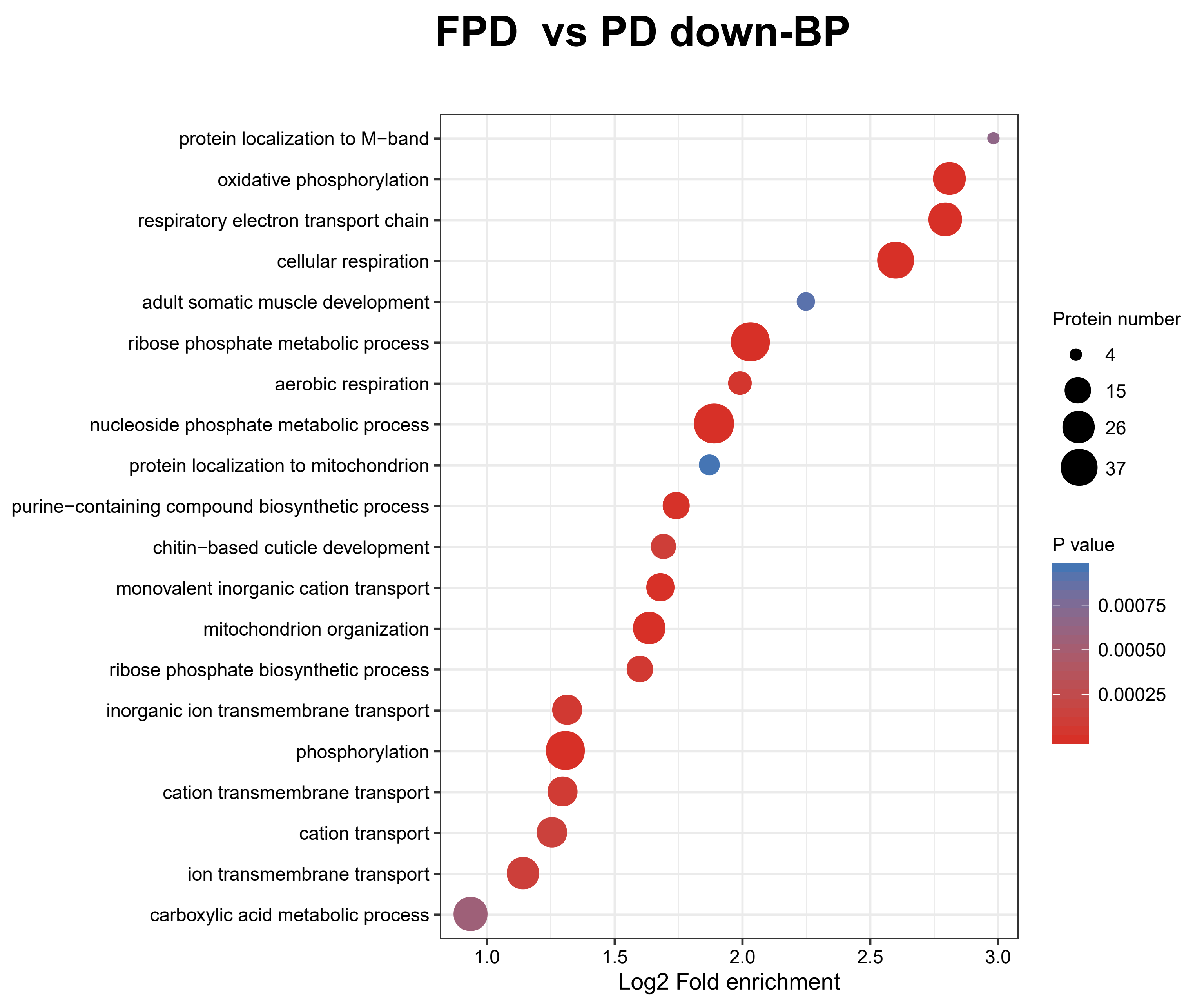
FPD vs PD down-BP
protein localization to M−band
oxidative phosphorylation
respiratory electron transport chain
cellular respiration
adult somatic muscle development
ribose phosphate metabolic process
aerobic respiration
nucleoside phosphate metabolic process
protein localization to mitochondrion
purine−containing compound biosynthetic process
chitin−based cuticle development
monovalent inorganic cation transport
mitochondrion organization
ribose phosphate biosynthetic process
inorganic ion transmembrane transport
phosphorylation
cation transmembrane transport
cation transport
ion transmembrane transport
carboxylic acid metabolic process
1.0
1.5
2.0
2.5
3.0
Log2 Fold enrichment
Protein number
4
15
26
37
P value
0.00075
0.00050
0.00025

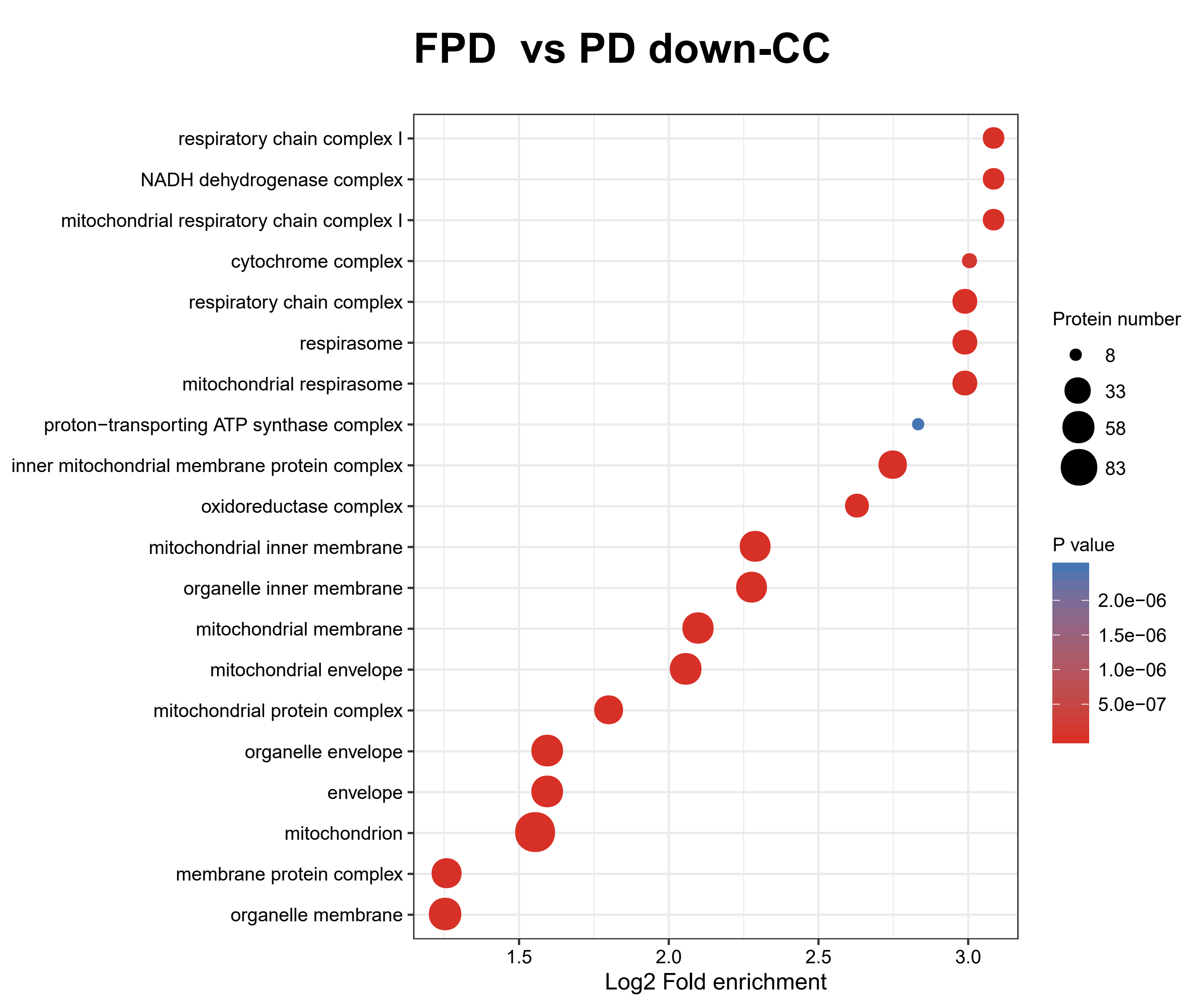
FPD vs PD down-CC
respiratory chain complex I
NADH dehydrogenase complex
mitochondrial respiratory chain complex I
cytochrome complex
respiratory chain complex
respirasome
mitochondrial respirasome
proton−transporting ATP synthase complex
inner mitochondrial membrane protein complex
oxidoreductase complex
mitochondrial inner membrane
organelle inner membrane
mitochondrial membrane
mitochondrial envelope
mitochondrial protein complex
organelle envelope
envelope
mitochondrion
membrane protein complex
organelle membrane
1.5
2.0
2.5
3.0
Log2 Fold enrichment
Protein number
8
33
58
83
P value
2.0e−06
1.5e−06
1.0e−06
5.0e−07

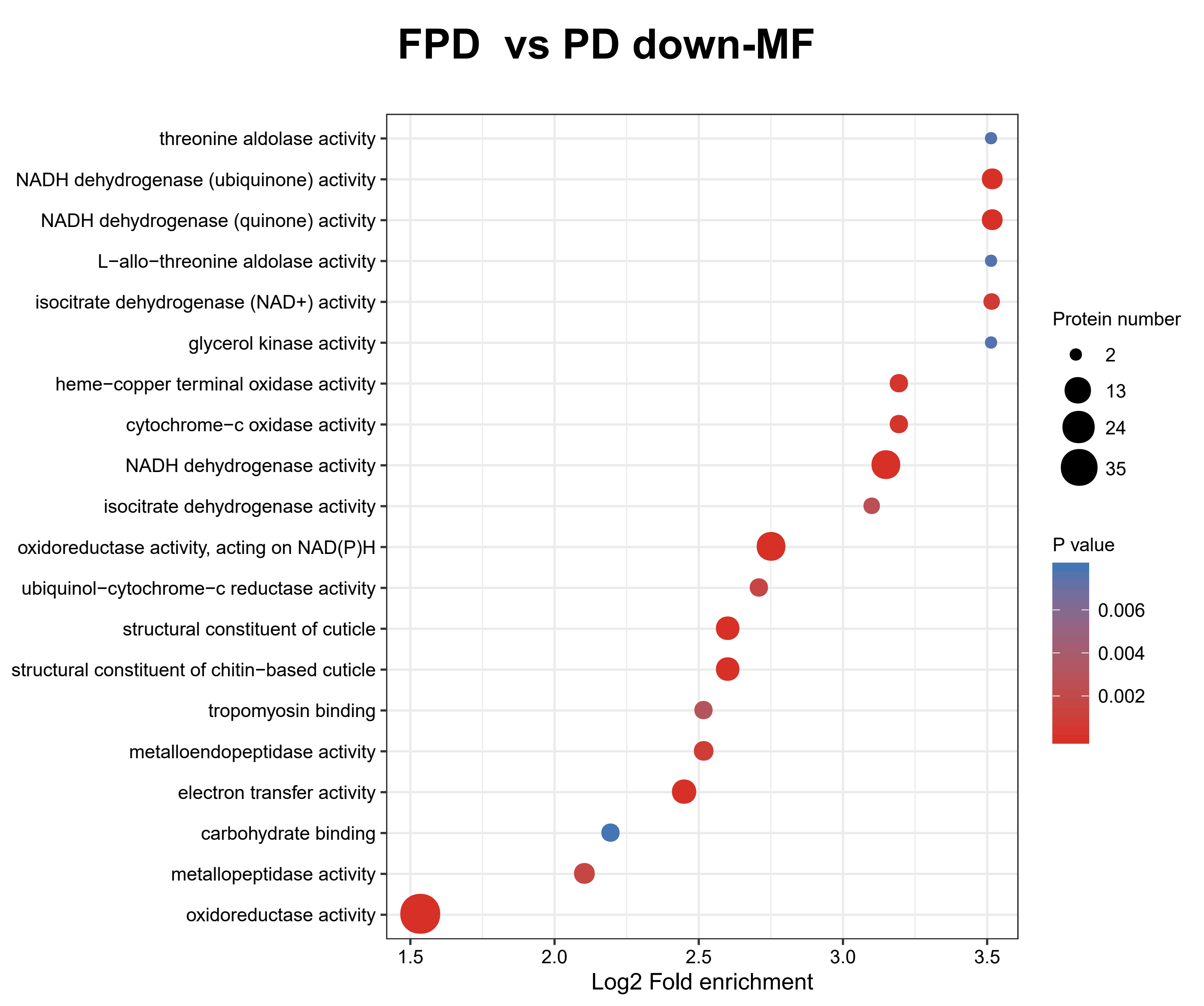
FPD vs PD down-MF
threonine aldolase activity
NADH dehydrogenase (ubiquinone) activity
NADH dehydrogenase (quinone) activity
L−allo−threonine aldolase activity
isocitrate dehydrogenase (NAD+) activity
glycerol kinase activity
heme−copper terminal oxidase activity
cytochrome−c oxidase activity
NADH dehydrogenase activity
isocitrate dehydrogenase activity
oxidoreductase activity, acting on NAD(P)H
ubiquinol−cytochrome−c reductase activity
structural constituent of cuticle
structural constituent of chitin−based cuticle
tropomyosin binding
metalloendopeptidase activity
electron transfer activity
carbohydrate binding
metallopeptidase activity
oxidoreductase activity
1.5
2.0
2.5
3.0
3.5
Log2 Fold enrichment
Protein number
2
13
24
35
P value
0.006
0.004
0.002

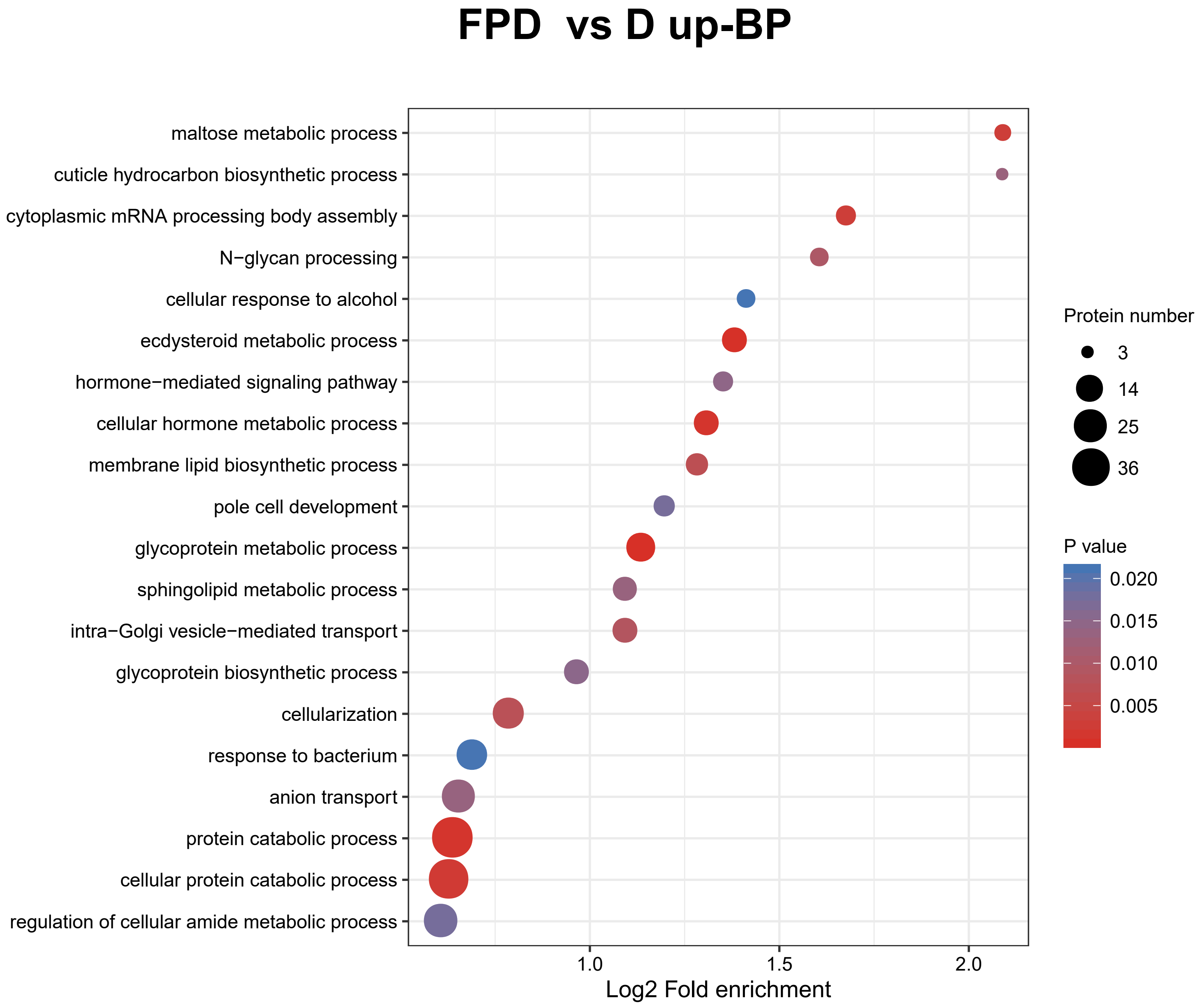
FPD vs D up-BP
maltose metabolic process
cuticle hydrocarbon biosynthetic process
cytoplasmic mRNA processing body assembly
N−glycan processing
cellular response to alcohol
ecdysteroid metabolic process
hormone−mediated signaling pathway
cellular hormone metabolic process
membrane lipid biosynthetic process
pole cell development
glycoprotein metabolic process
sphingolipid metabolic process
intra−Golgi vesicle−mediated transport
glycoprotein biosynthetic process
cellularization
response to bacterium
anion transport
protein catabolic process
cellular protein catabolic process
regulation of cellular amide metabolic process
1.0
1.5
2.0
Log2 Fold enrichment
Protein number
3
14
25
36
P value
0.020
0.015
0.010
0.005

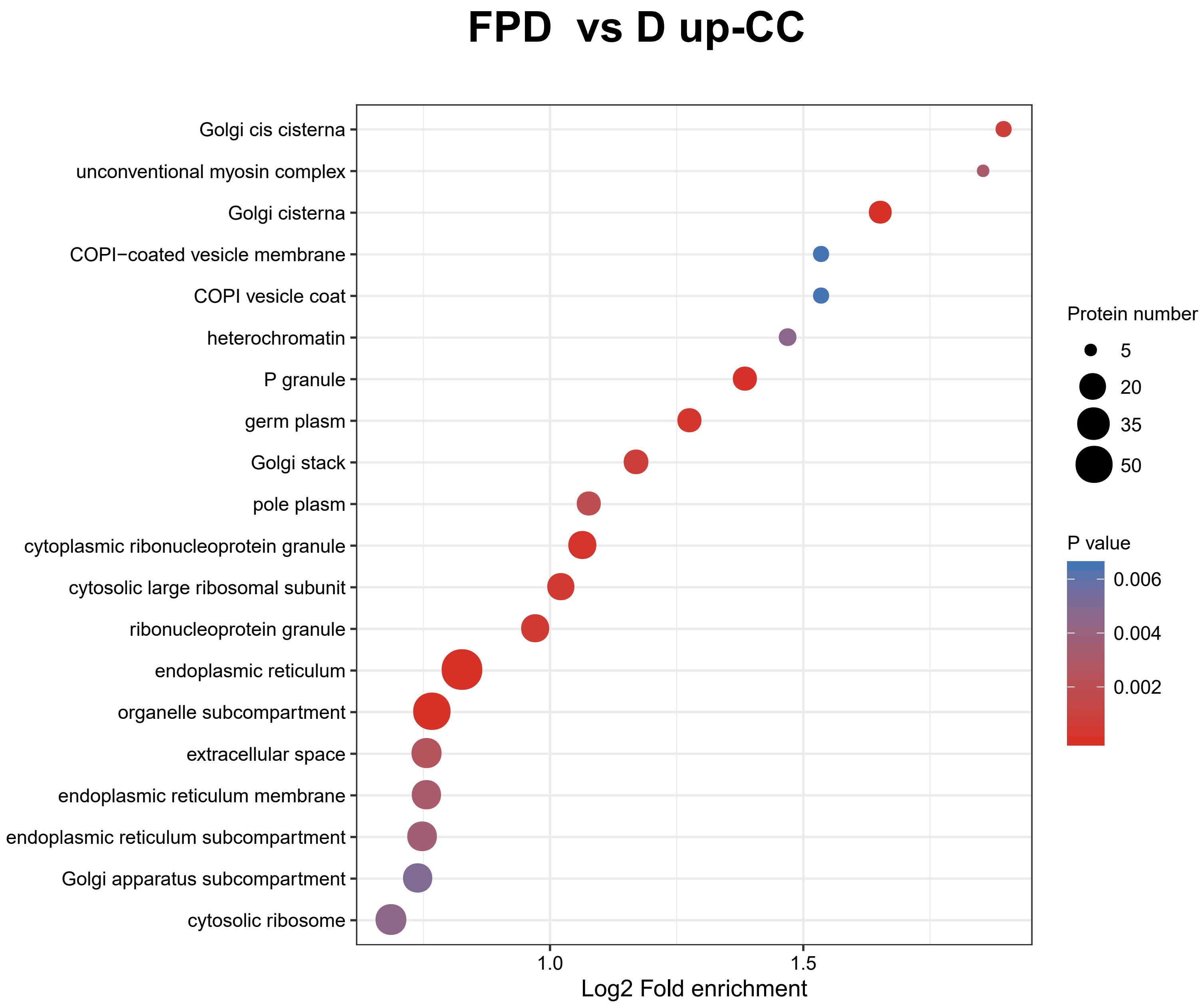

FPD vs D up-CC
Golgi cis cisterna
unconventional myosin complex
Golgi cisterna
COPI−coated vesicle membrane
COPI vesicle coat
heterochromatin
P granule
germ plasm
Golgi stack
pole plasm
cytoplasmic ribonucleoprotein granule
cytosolic large ribosomal subunit
ribonucleoprotein granule
endoplasmic reticulum
organelle subcompartment
extracellular space
endoplasmic reticulum membrane
endoplasmic reticulum subcompartment
Golgi apparatus subcompartment
cytosolic ribosome
1.0
1.5
Log2 Fold enrichment
Protein number
5
20
35
50
P value
0.006
0.004
0.002

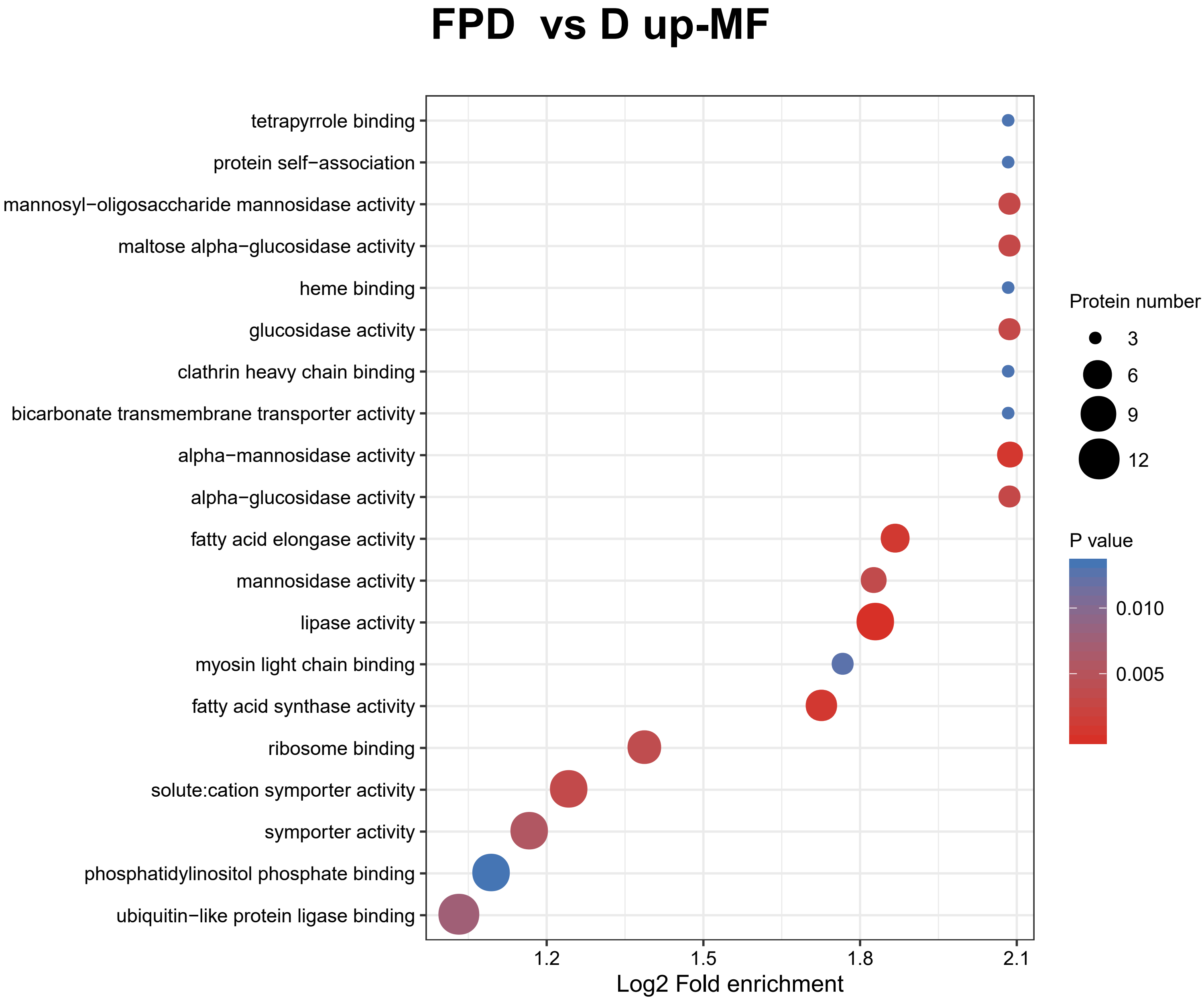

FPD vs D up-MF
tetrapyrrole binding
protein self−association
mannosyl−oligosaccharide mannosidase activity
maltose alpha−glucosidase activity
heme binding
glucosidase activity
clathrin heavy chain binding
bicarbonate transmembrane transporter activity
alpha−mannosidase activity
alpha−glucosidase activity
fatty acid elongase activity
mannosidase activity
lipase activity
myosin light chain binding
fatty acid synthase activity
ribosome binding
solute:cation symporter activity
symporter activity
phosphatidylinositol phosphate binding
ubiquitin−like protein ligase binding
1.2
1.5
1.8
2.1
Log2 Fold enrichment
Protein number
3
6
9
12
P value
0.010
0.005

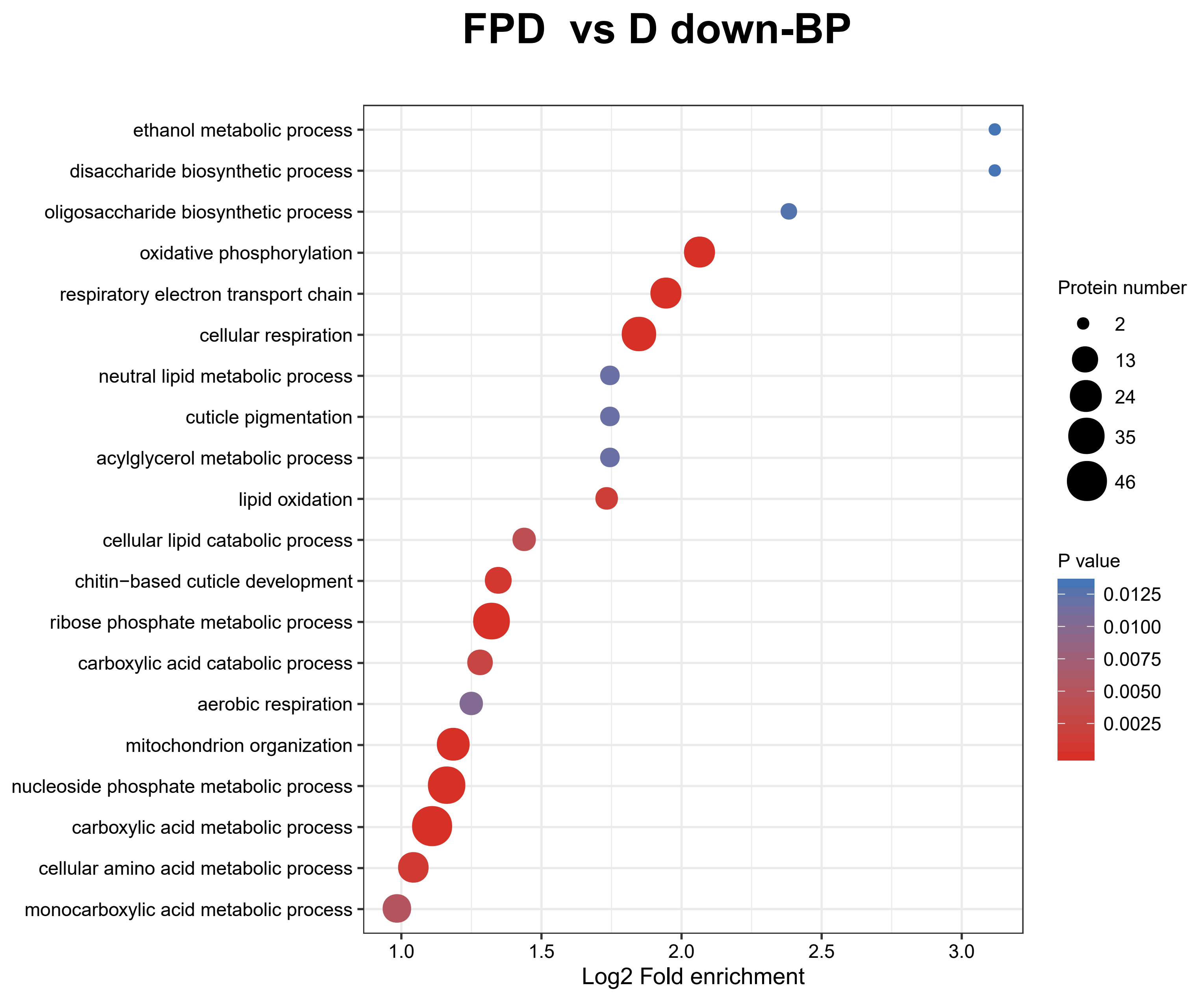

FPD vs D down-BP
ethanol metabolic process
disaccharide biosynthetic process
oligosaccharide biosynthetic process
oxidative phosphorylation
respiratory electron transport chain
cellular respiration
neutral lipid metabolic process
cuticle pigmentation
acylglycerol metabolic process
lipid oxidation
cellular lipid catabolic process
chitin−based cuticle development
ribose phosphate metabolic process
carboxylic acid catabolic process
aerobic respiration
mitochondrion organization
nucleoside phosphate metabolic process
carboxylic acid metabolic process
cellular amino acid metabolic process
monocarboxylic acid metabolic process
1.0
1.5
2.0
2.5
3.0
Log2 Fold enrichment
Protein number
2
13
24
35
46
P value
0.0125
0.0100
0.0075
0.0050
0.0025

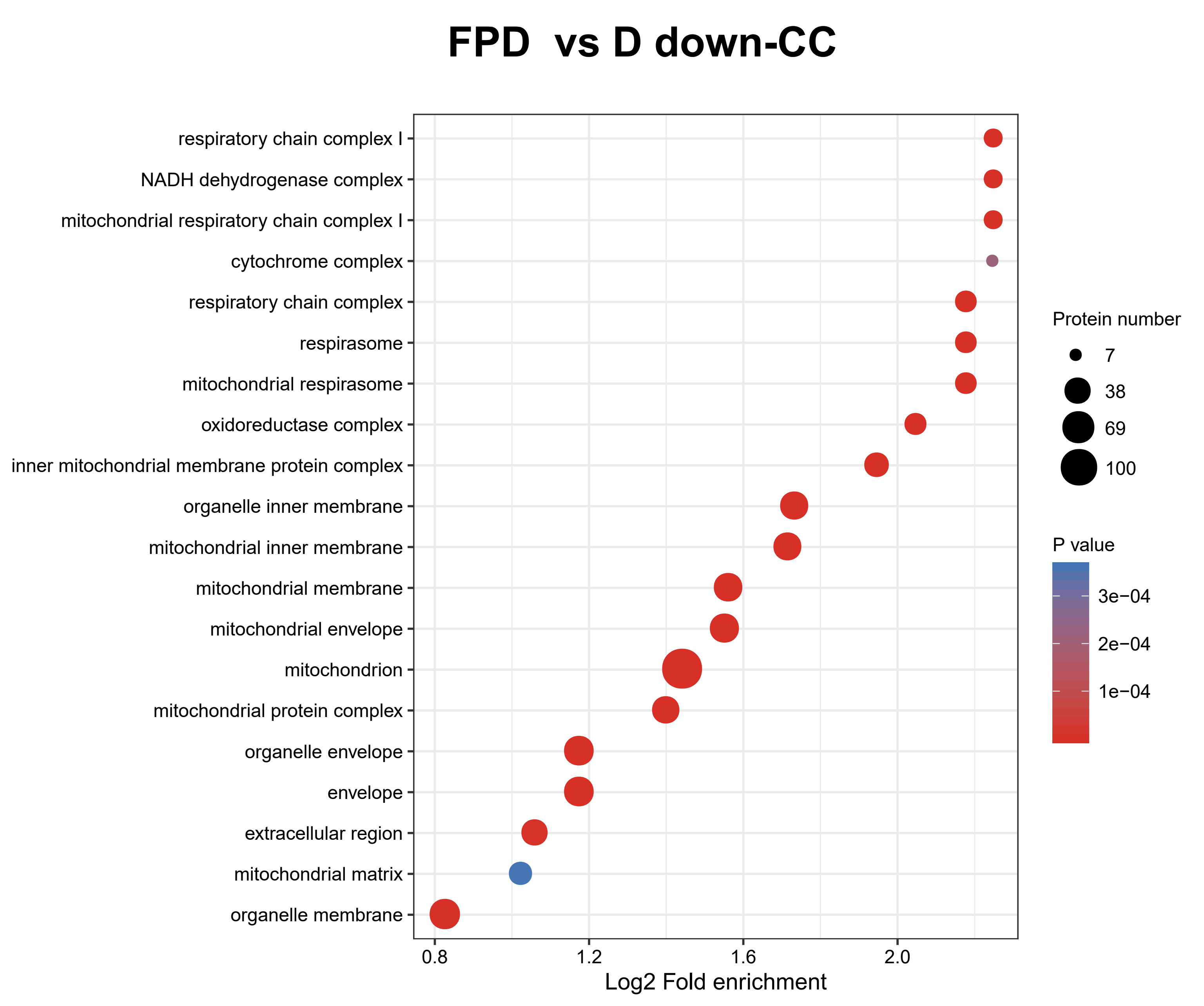
FPD vs D down-CC
respiratory chain complex I
NADH dehydrogenase complex
mitochondrial respiratory chain complex I
cytochrome complex
respiratory chain complex
respirasome
mitochondrial respirasome
oxidoreductase complex
inner mitochondrial membrane protein complex
organelle inner membrane
mitochondrial inner membrane
mitochondrial membrane
mitochondrial envelope
mitochondrion
mitochondrial protein complex
organelle envelope
envelope
extracellular region
mitochondrial matrix
organelle membrane
0.8
1.2
1.6
2.0
Log2 Fold enrichment
Protein number
7
38
69
100
P value
3e−04
2e−04
1e−04

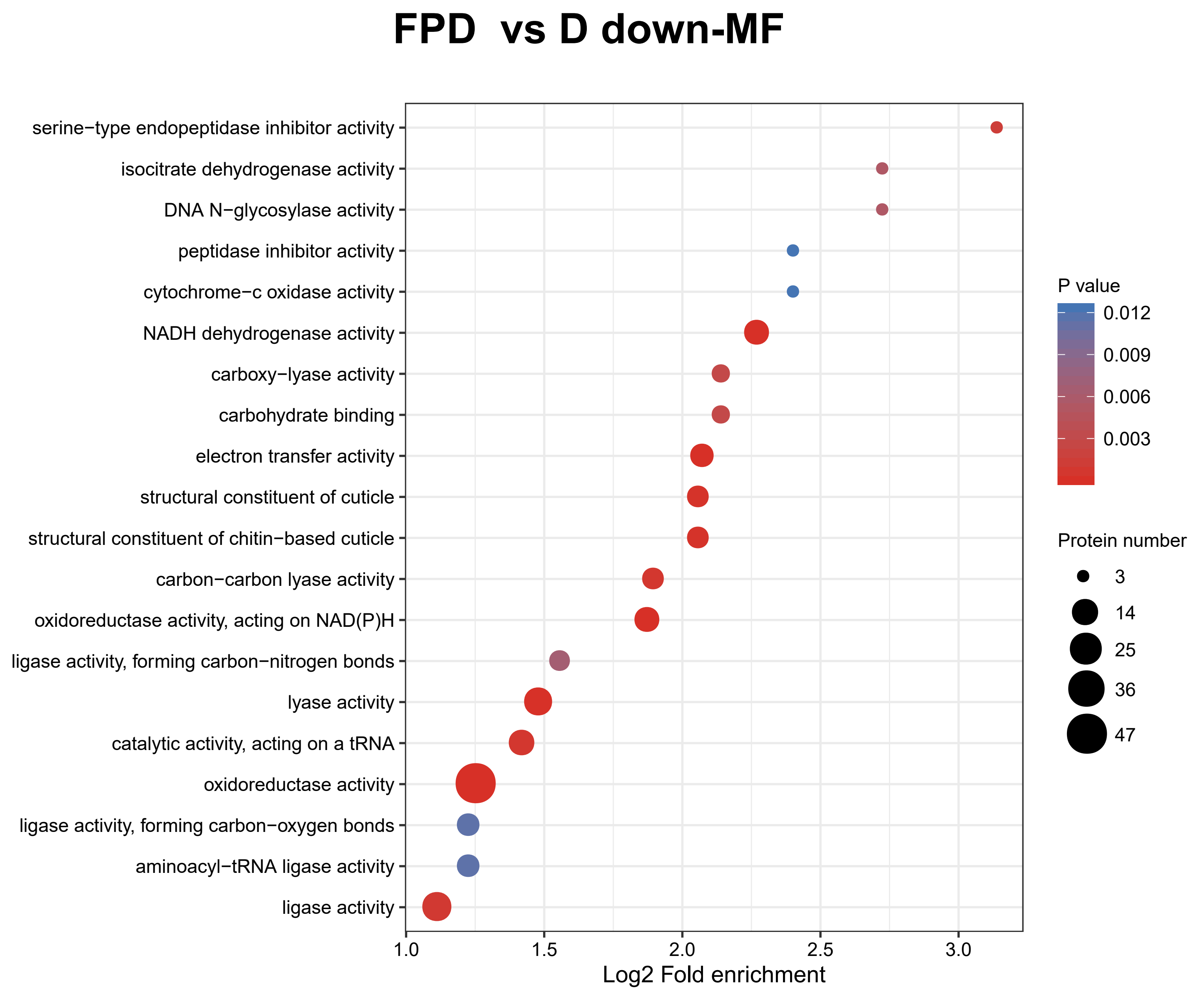
FPD vs D down-MF
serine−type endopeptidase inhibitor activity
isocitrate dehydrogenase activity
DNA N−glycosylase activity
peptidase inhibitor activity
cytochrome−c oxidase activity
NADH dehydrogenase activity
carboxy−lyase activity
carbohydrate binding
electron transfer activity
structural constituent of cuticle
structural constituent of chitin−based cuticle
carbon−carbon lyase activity
oxidoreductase activity, acting on NAD(P)H
ligase activity, forming carbon−nitrogen bonds
lyase activity
catalytic activity, acting on a tRNA
oxidoreductase activity
ligase activity, forming carbon−oxygen bonds
aminoacyl−tRNA ligase activity
ligase activity
1.0
1.5
2.0
2.5
3.0
Log2 Fold enrichment
P value
0.012
0.009
0.006
0.003
Protein number
3
14
25
36
47
