# Supplementary material for: Integrative Proteomic and Phosphoproteomic Analyses Revealed Complex Mechanisms Underlying Reproductive Diapause in Bombus terrestris Queens
Source: Insects. 2022 Sep 23;13(10):862. doi: 10.3390/insects13100862 (PMC9604461; doi:10.3390/insects13100862)

Supplementary Figure S7: Overview of phosphoproteomic analyses. (A) The distributions of raw MS/MS length and charge of peptides quantified from proteomic data. (B) Reproducibility analysis of the samples. (C) The amounts of differentially quantified phosphosites and phosphoproteins in different comparable groups.

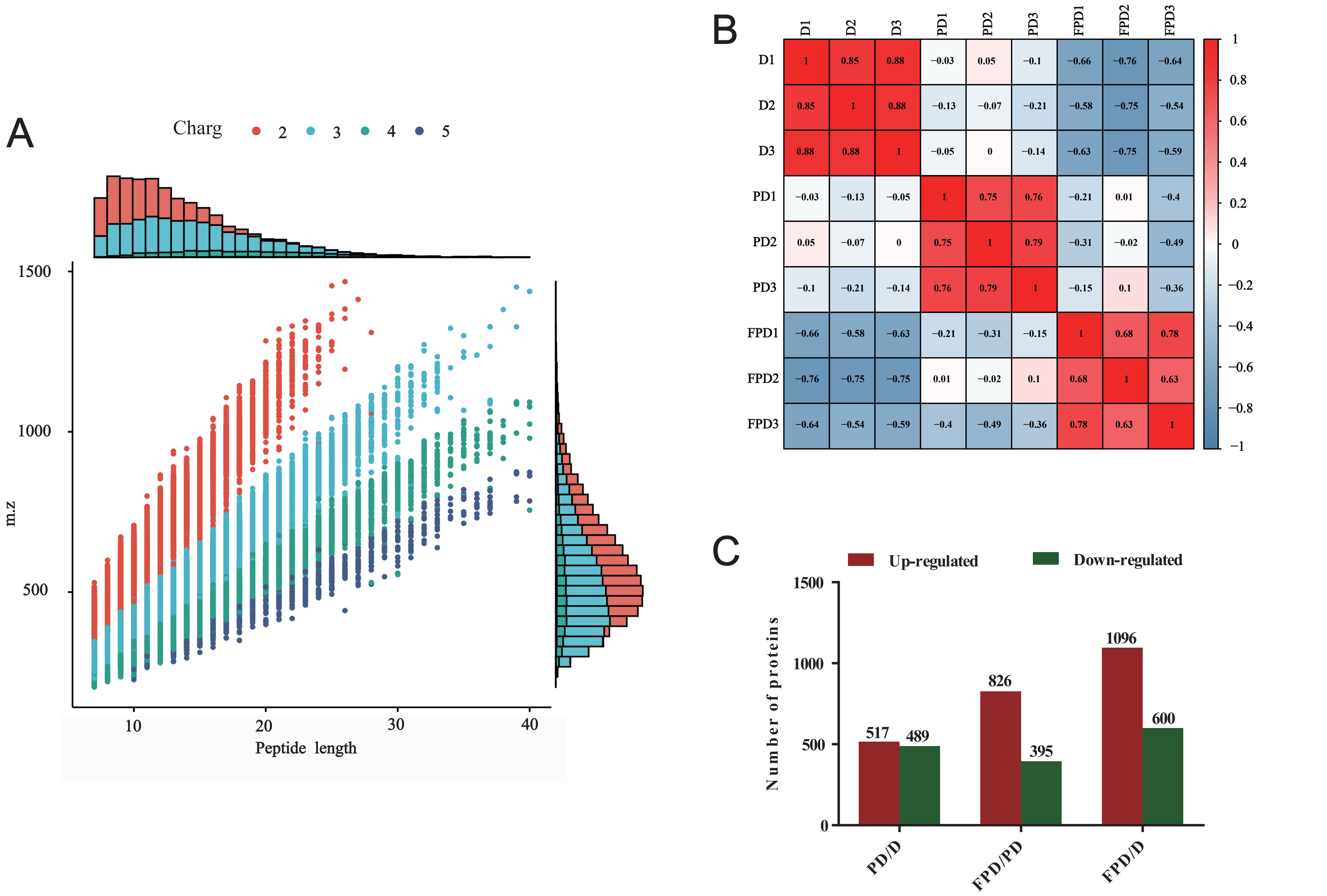

Supplement: Supplementary file 1 [file insects-13-00862-s001.zip › insects-1876268-supplementary/insects-1876268-proofed-supplementary/Figure S7.pdf]
