# Supplementary material for: Integrative Proteomic and Phosphoproteomic Analyses Revealed Complex Mechanisms Underlying Reproductive Diapause in Bombus terrestris Queens
Source: Insects. 2022 Sep 23;13(10):862. doi: 10.3390/insects13100862 (PMC9604461; doi:10.3390/insects13100862)
Supplement: Supplementary file 1 [file insects-13-00862-s001.zip › insects-1876268-supplementary/insects-1876268-proofed-supplementary/Figure S8.pdf]

Supplementary Figure S8: KEGG analysis of DEPPs in each comparable group.

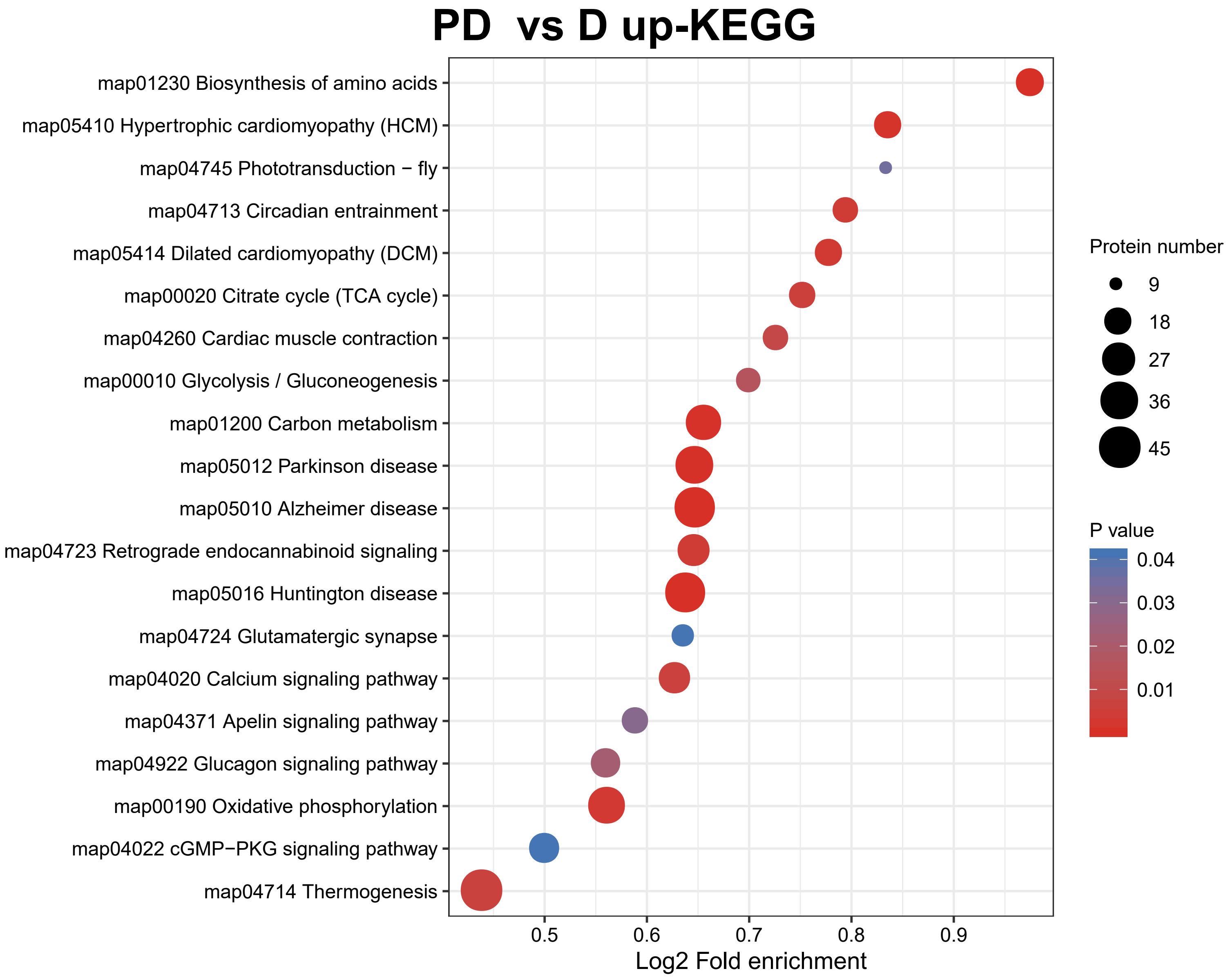

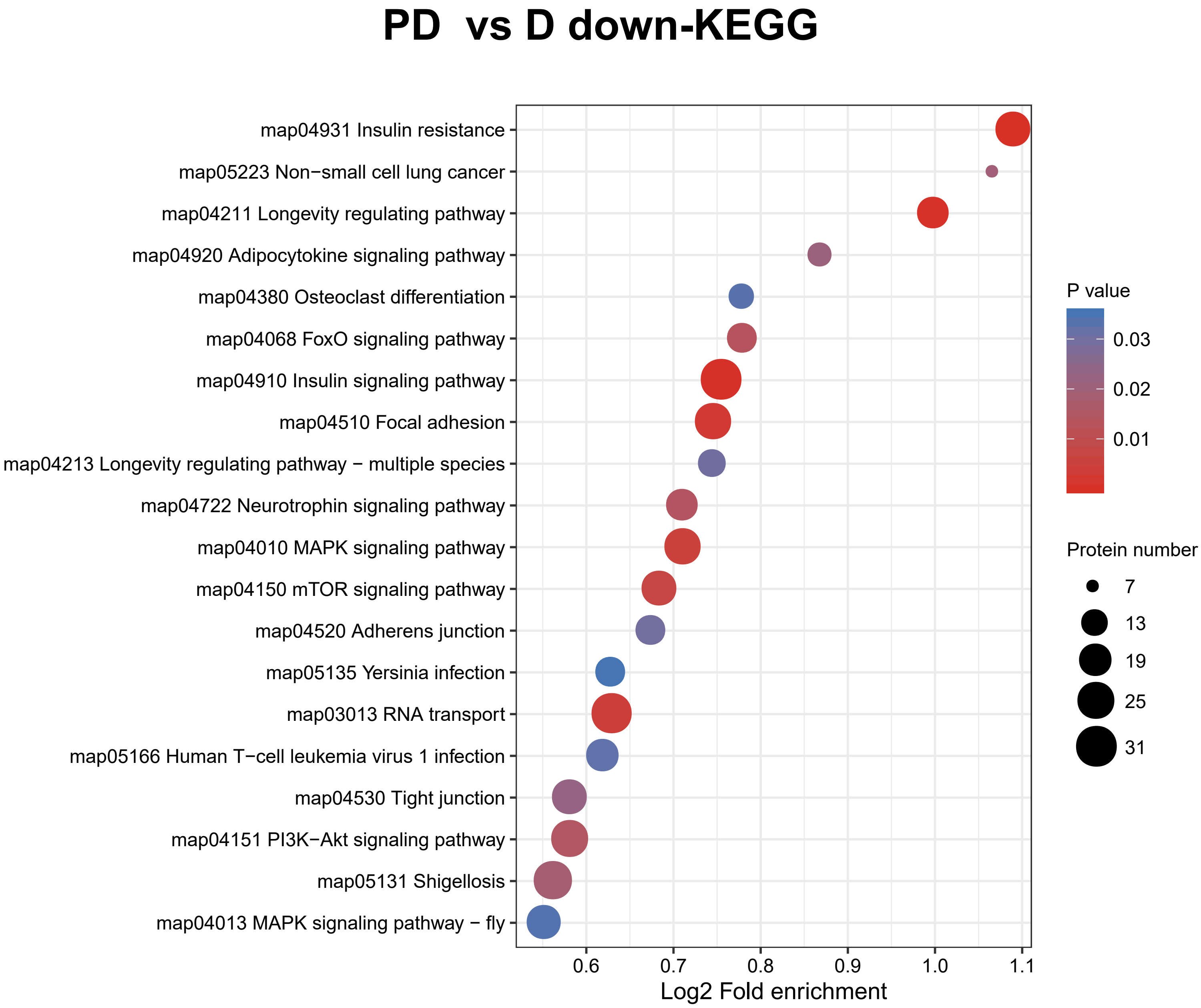
PD vs D down-KEGG
map04931 Insulin resistance
map05223 Non−small cell lung cancer
map04211 Longevity regulating pathway
map04920 Adipocytokine signaling pathway
map04380 Osteoclast differentiation
map04068 FoxO signaling pathway
map04910 Insulin signaling pathway
map04510 Focal adhesion
map04213 Longevity regulating pathway − multiple species
map04722 Neurotrophin signaling pathway
map04010 MAPK signaling pathway
map04150 mTOR signaling pathway
map04520 Adherens junction
map05135 Yersinia infection
map03013 RNA transport
map05166 Human T−cell leukemia virus 1 infection
map04530 Tight junction
map04151 PI3K−Akt signaling pathway
map05131 Shigellosis
map04013 MAPK signaling pathway − fly
0.6
0.7
0.8
0.9
1.0
1.1
Log2 Fold enrichment
P value
0.03
0.02
0.01
Protein number
7
13
19
25
31

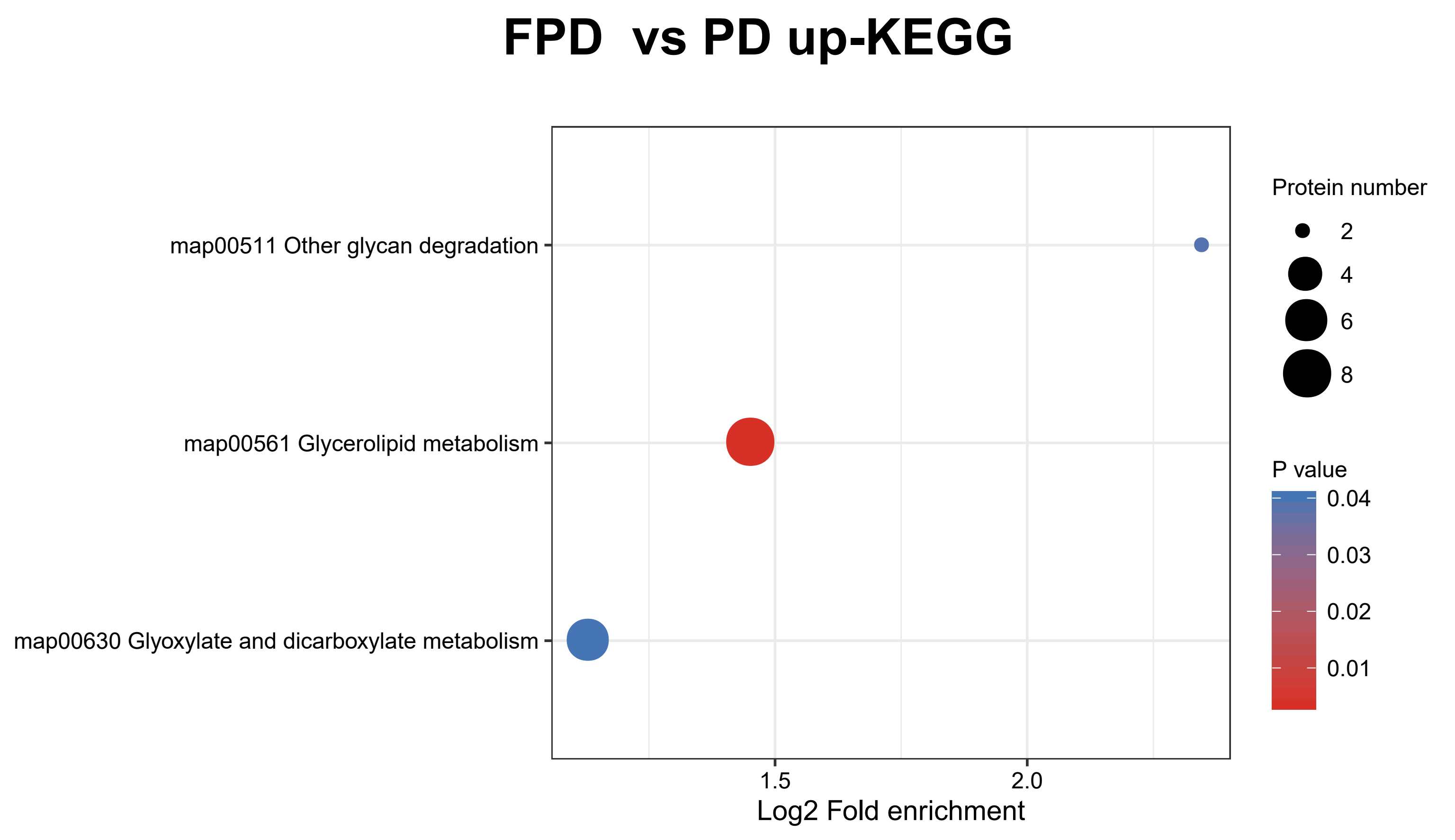
FPD vs PD up-KEGG
map00511 Other glycan degradation
map00561 Glycerolipid metabolism
map00630 Glyoxylate and dicarboxylate metabolism
1.5
2.0
Log2 Fold enrichment
Protein number
2
4
6
8
P value
0.04
0.03
0.02
0.01

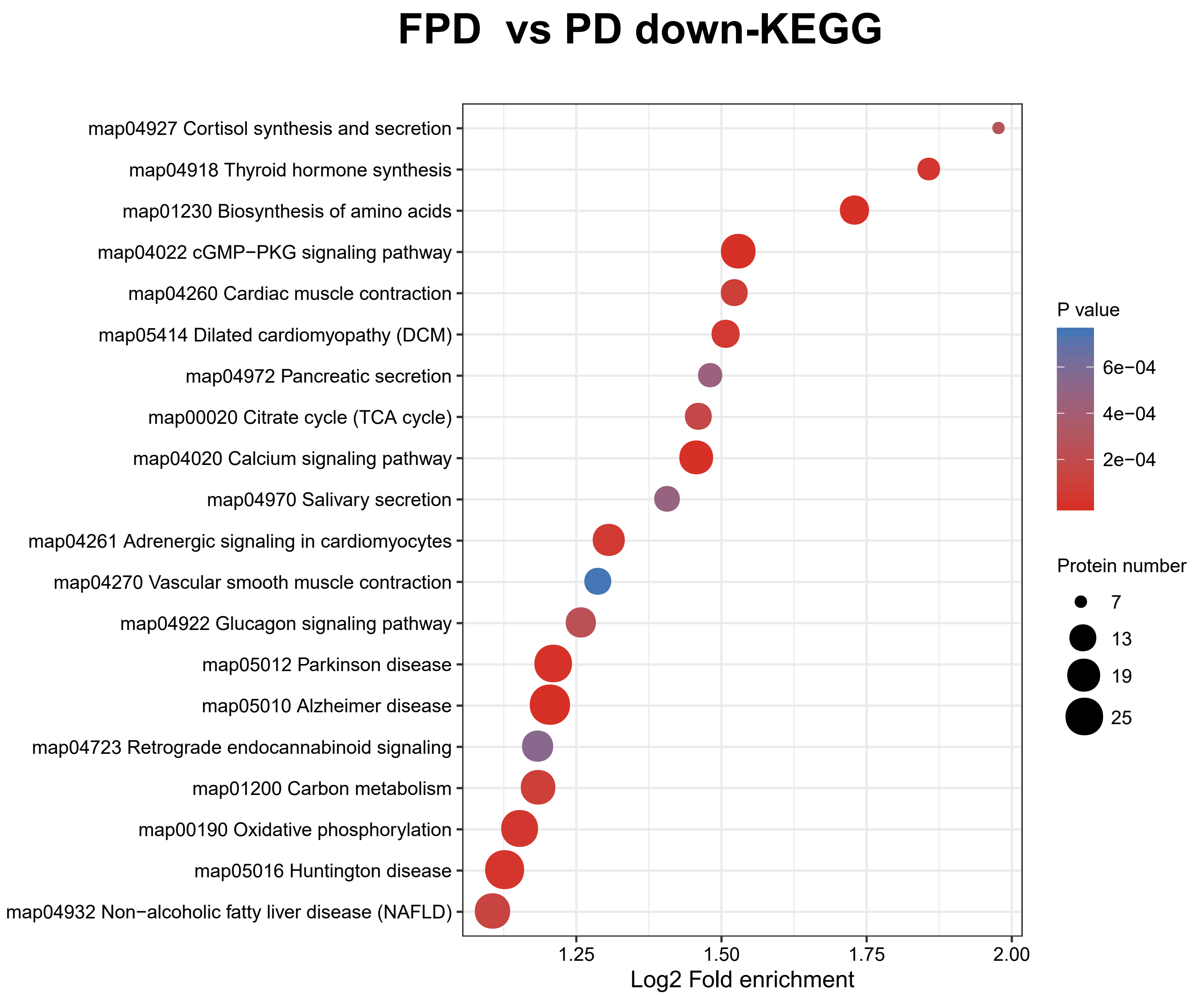
FPD vs PD down-KEGG
map04927 Cortisol synthesis and secretion
map04918 Thyroid hormone synthesis
map01230 Biosynthesis of amino acids
map04022 cGMP−PKG signaling pathway
map04260 Cardiac muscle contraction
map05414 Dilated cardiomyopathy (DCM)
map04972 Pancreatic secretion
map00020 Citrate cycle (TCA cycle)
map04020 Calcium signaling pathway
map04970 Salivary secretion
map04261 Adrenergic signaling in cardiomyocytes
map04270 Vascular smooth muscle contraction
map04922 Glucagon signaling pathway
map05012 Parkinson disease
map05010 Alzheimer disease
map04723 Retrograde endocannabinoid signaling
map01200 Carbon metabolism
map00190 Oxidative phosphorylation
map05016 Huntington disease
map04932 Non−alcoholic fatty liver disease (NAFLD)
1.25
1.50
1.75
2.00
Log2 Fold enrichment
P value
6e−04
4e−04
2e−04
Protein number
7
13
19
25

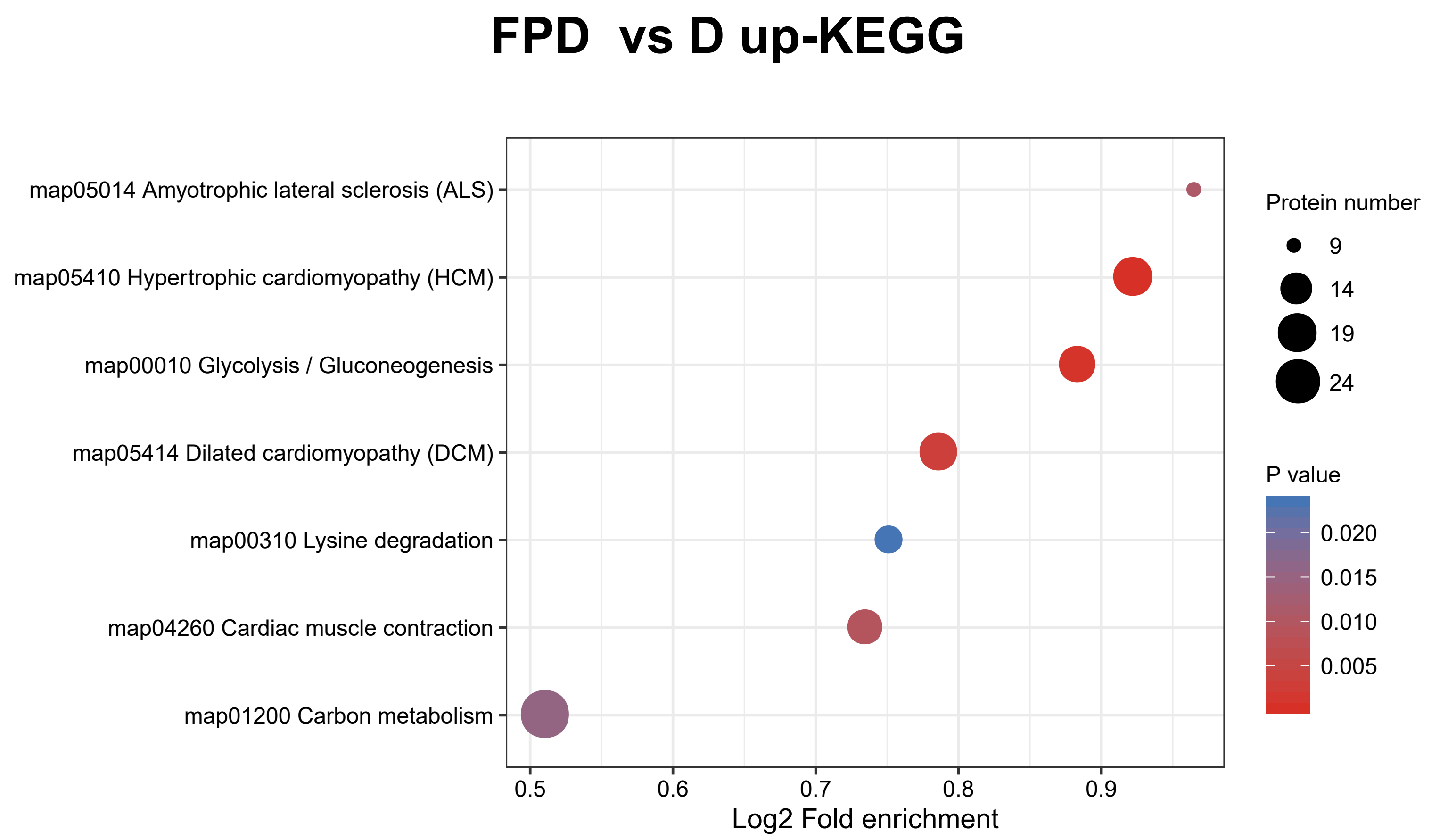
FPD vs D up-KEGG
map05014 Amyotrophic lateral sclerosis (ALS)
map05410 Hypertrophic cardiomyopathy (HCM)
map00010 Glycolysis / Gluconeogenesis
map05414 Dilated cardiomyopathy (DCM)
map00310 Lysine degradation
map04260 Cardiac muscle contraction
map01200 Carbon metabolism
0.5
0.6
0.7
0.8
0.9
Log2 Fold enrichment
Protein number
9
14
19
24
P value
0.020
0.015
0.010
0.005

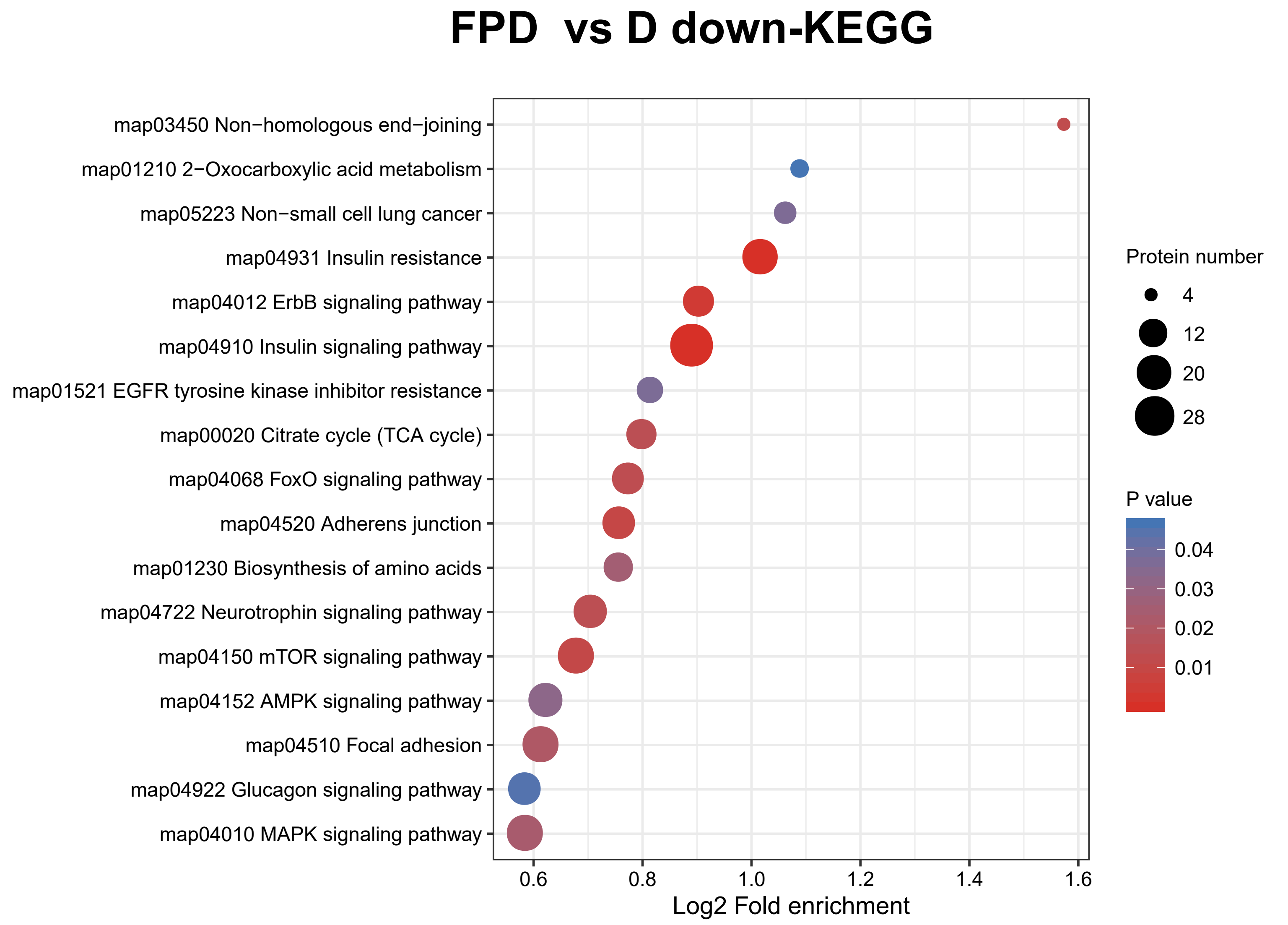
FPD vs D down-KEGG
map03450 Non−homologous end−joining
map01210 2−Oxocarboxylic acid metabolism
map05223 Non−small cell lung cancer
map04931 Insulin resistance
map04012 ErbB signaling pathway
map04910 Insulin signaling pathway
map01521 EGFR tyrosine kinase inhibitor resistance
map00020 Citrate cycle (TCA cycle)
map04068 FoxO signaling pathway
map04520 Adherens junction
map01230 Biosynthesis of amino acids
map04722 Neurotrophin signaling pathway
map04150 mTOR signaling pathway
map04152 AMPK signaling pathway
map04510 Focal adhesion
map04922 Glucagon signaling pathway
map04010 MAPK signaling pathway
0.6
0.8
1.0
1.2
1.4
1.6
Log2 Fold enrichment
Protein number
4
12
20
28
P value
0.04
0.03
0.02
0.01
