# Supplementary material for: Integrative Proteomic and Phosphoproteomic Analyses Revealed Complex Mechanisms Underlying Reproductive Diapause in Bombus terrestris Queens
Source: Insects. 2022 Sep 23;13(10):862. doi: 10.3390/insects13100862 (PMC9604461; doi:10.3390/insects13100862)
Supplement: Supplementary file 1 [file insects-13-00862-s001.zip › insects-1876268-supplementary/insects-1876268-proofed-supplementary/Figure S9.pdf]

Supplementary Figure S9: Protein domain analysis of DEPPs in each comparable group.

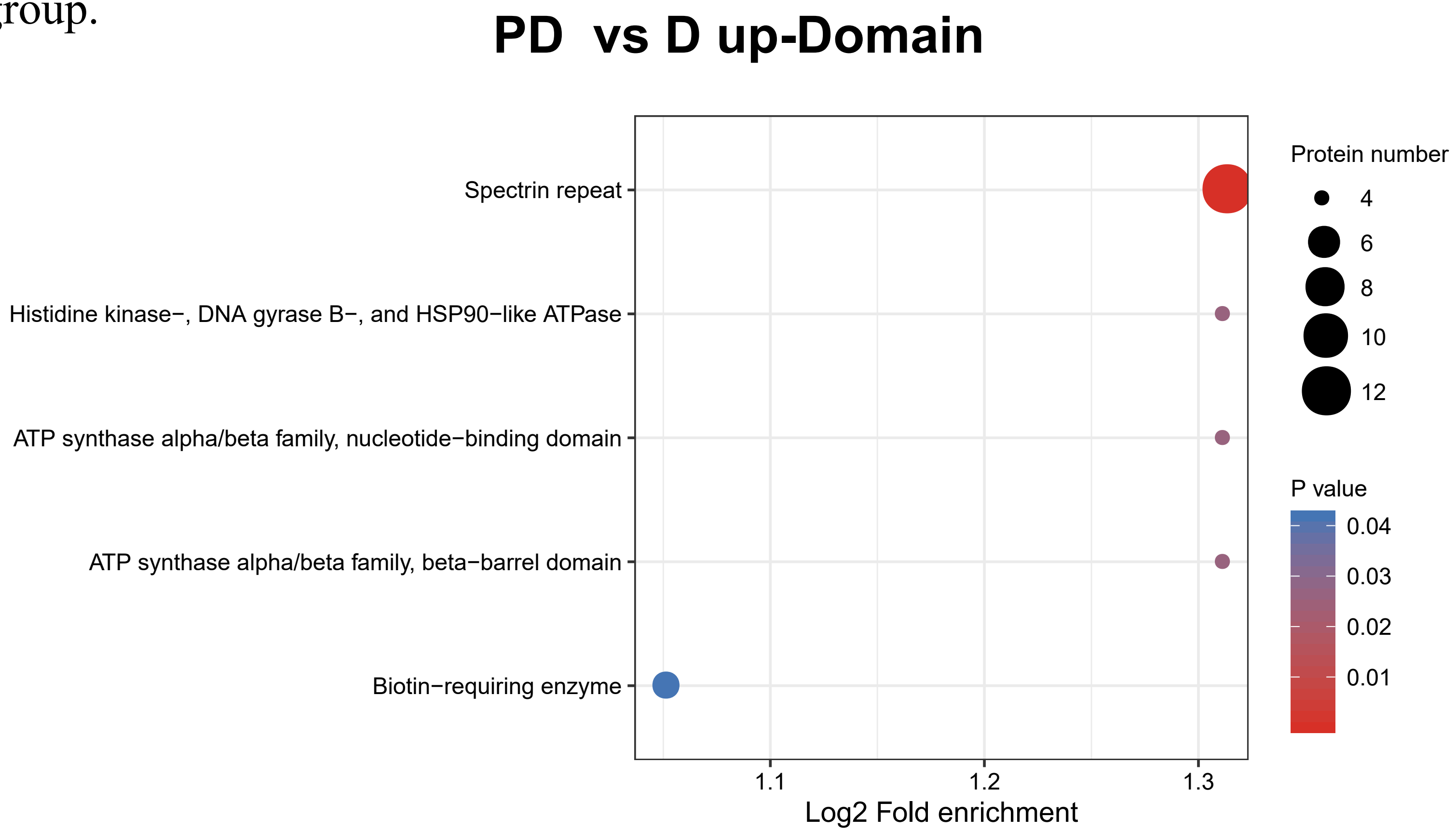

PD vs D down-Domain

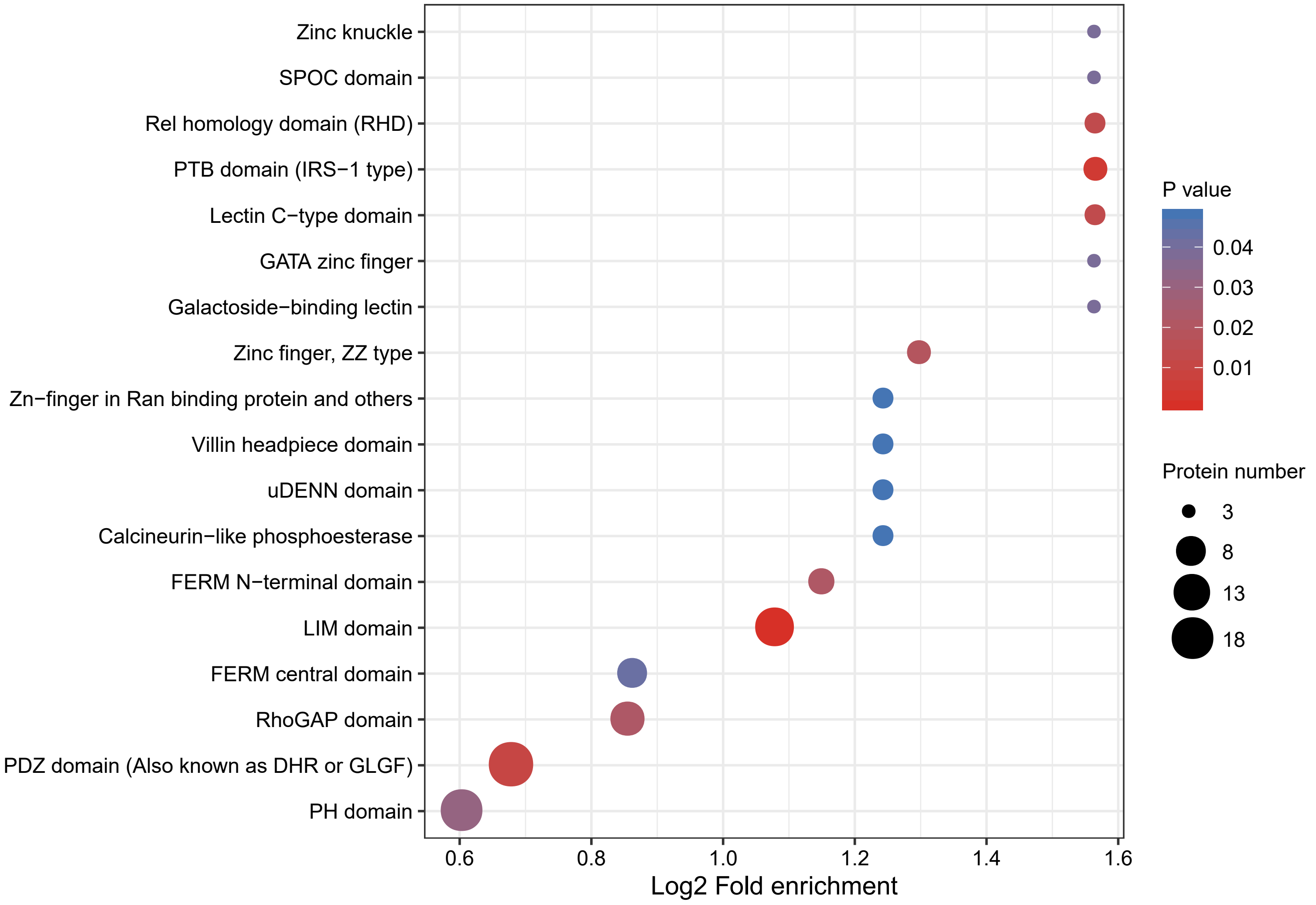

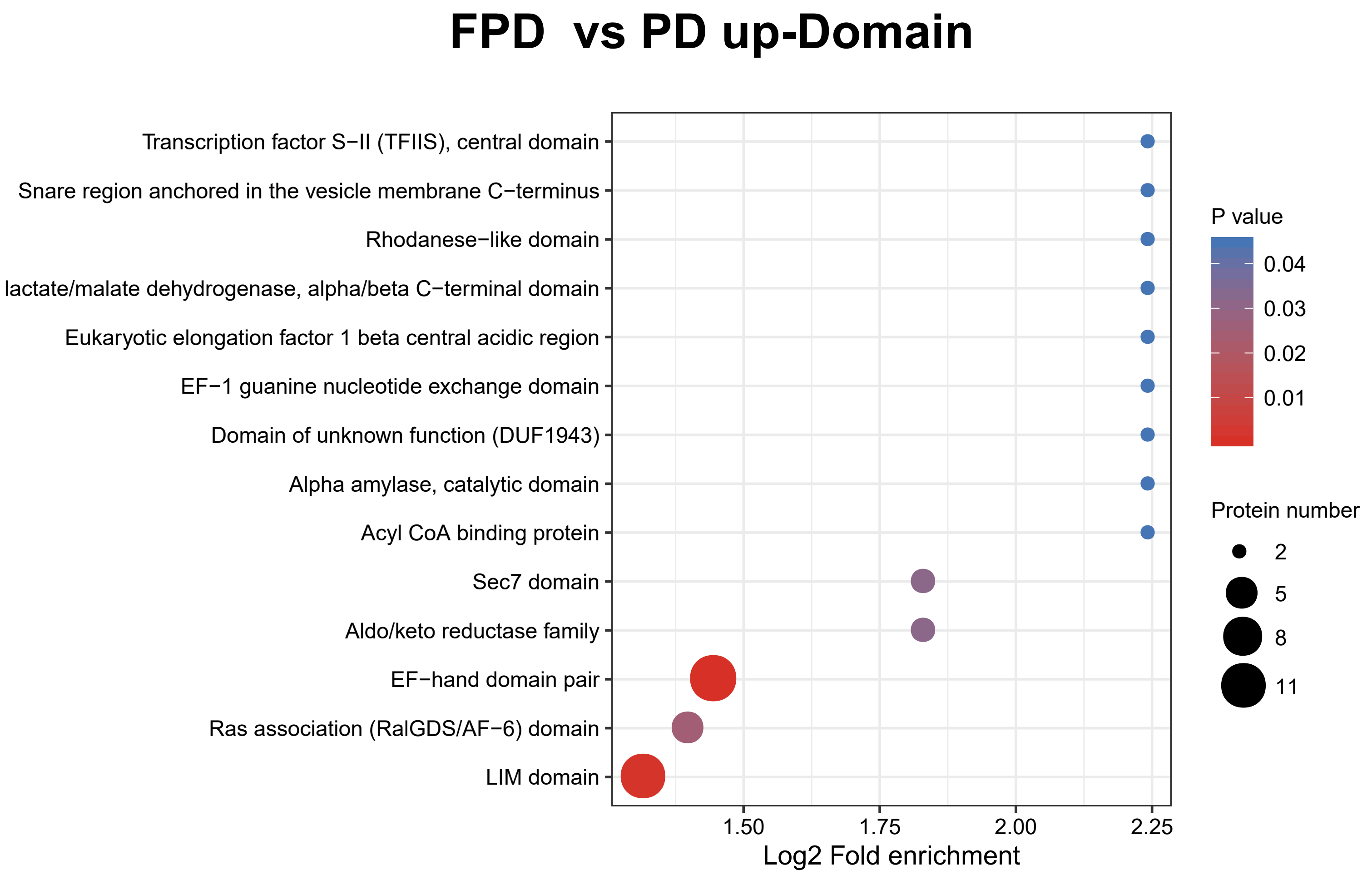

FPD vs PD up-Domain
Transcription factor S−II (TFIIS), central domain
Snare region anchored in the vesicle membrane C−terminus
Rhodanese−like domain
lactate/malate dehydrogenase, alpha/beta C−terminal domain
Eukaryotic elongation factor 1 beta central acidic region
EF−1 guanine nucleotide exchange domain
Domain of unknown function (DUF1943)
Alpha amylase, catalytic domain
Acyl CoA binding protein
Sec7 domain
Aldo/keto reductase family
EF−hand domain pair
Ras association (RalGDS/AF−6) domain
LIM domain
1.50
1.75
2.00
2.25
Log2 Fold enrichment
P value
0.04
0.03
0.02
0.01
Protein number
2
5
8
11

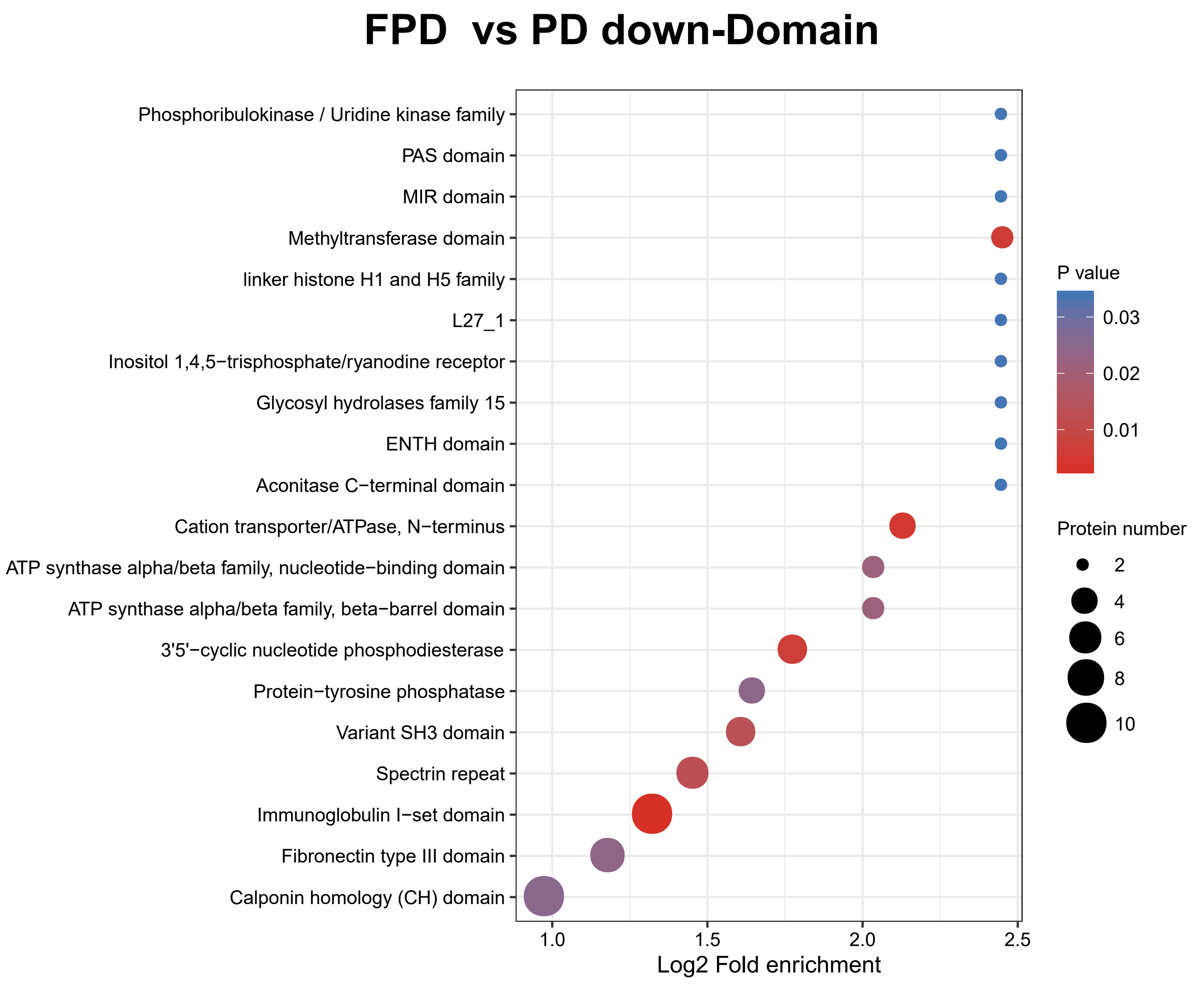

FPD vs PD down-Domain
Phosphoribulokinase / Uridine kinase family
PAS domain
MIR domain
Methyltransferase domain
linker histone H1 and H5 family
L27_1
Inositol 1,4,5−trisphosphate/ryanodine receptor
Glycosyl hydrolases family 15
ENTH domain
Aconitase C−terminal domain
Cation transporter/ATPase, N−terminus
ATP synthase alpha/beta family, nucleotide−binding domain
ATP synthase alpha/beta family, beta−barrel domain
3'5'−cyclic nucleotide phosphodiesterase
Protein−tyrosine phosphatase
Variant SH3 domain
Spectrin repeat
Immunoglobulin I−set domain
Fibronectin type III domain
Calponin homology (CH) domain
1.0
1.5
2.0
2.5
Log2 Fold enrichment
P value
0.03
0.02
0.01
Protein number
2
4
6
8
10

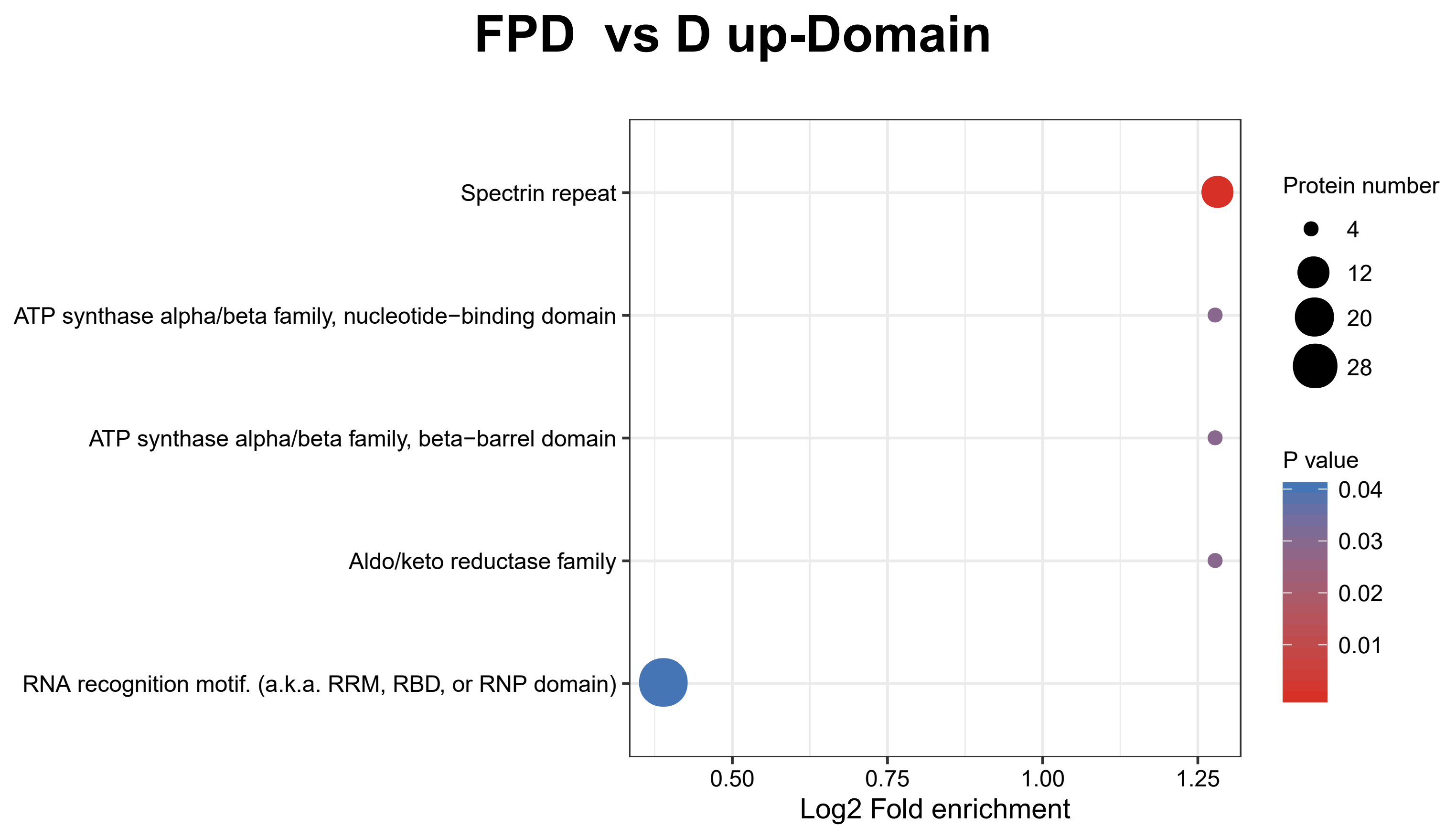

FPD vs D up-Domain
Spectrin repeat
ATP synthase alpha/beta family, nucleotide−binding domain
ATP synthase alpha/beta family, beta−barrel domain
Aldo/keto reductase family
RNA recognition motif. (a.k.a. RRM, RBD, or RNP domain)
0.50
0.75
1.00
1.25
Log2 Fold enrichment
Protein number
4
12
20
28
P value
0.04
0.03
0.02
0.01

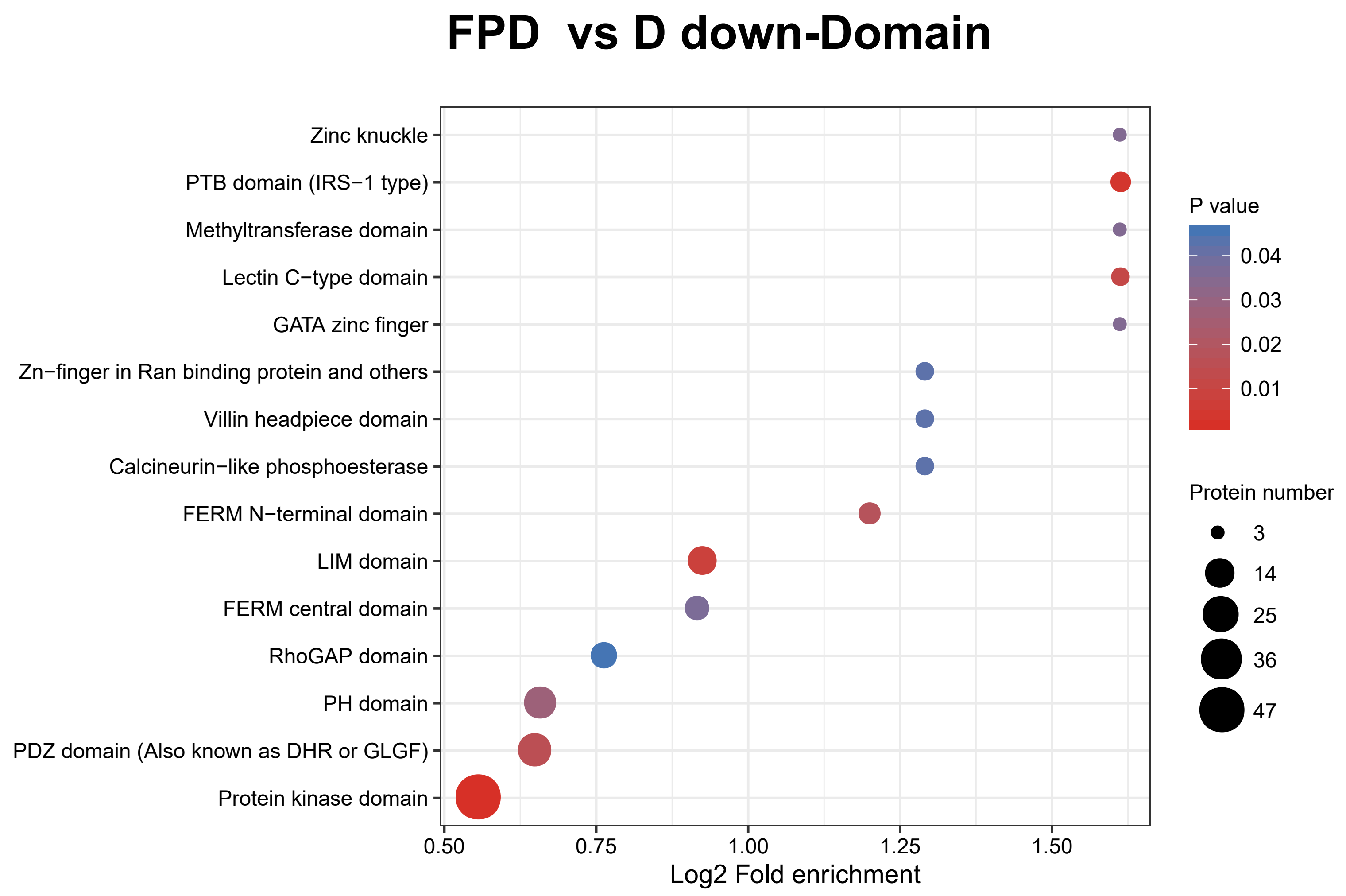

FPD vs D down-Domain
Zinc knuckle
PTB domain (IRS−1 type)
Methyltransferase domain
Lectin C−type domain
GATA zinc finger
Zn−finger in Ran binding protein and others
Villin headpiece domain
Calcineurin−like phosphoesterase
FERM N−terminal domain
LIM domain
FERM central domain
RhoGAP domain
PH domain
PDZ domain (Also known as DHR or GLGF)
Protein kinase domain
0.50
0.75
1.00
1.25
1.50
Log2 Fold enrichment
P value
0.04
0.03
0.02
0.01
Protein number
3
14
25
36
47
